# DYZ1 arrays show sequence variation between the monozygotic males

**DOI:** 10.1186/1471-2156-15-19

**Published:** 2014-02-04

**Authors:** Sandeep Kumar Yadav, Anju Kumari, Saleem Javed, Sher Ali

**Affiliations:** 1 Molecular Genetics Laboratory, National Institute of Immunology, Aruna Asaf Ali Marg, New Delhi 110067, India; 2 Jamia Hamdard, Hamdard Nagar, New Delhi 110062, India

**Keywords:** Monozygotic twins, Y chromosome, DYZ1

## Abstract

**Background:**

Monozygotic twins (MZT) are an important resource for genetical studies in the context of normal and diseased genomes. In the present study we used DYZ1, a satellite fraction present in the form of tandem arrays on the long arm of the human Y chromosome, as a tool to uncover sequence variations between the monozygotic males.

**Results:**

We detected copy number variation, frequent insertions and deletions within the sequences of DYZ1 arrays amongst all the three sets of twins used in the present study. MZT1b showed loss of 35 bp compared to that in 1a, whereas 2a showed loss of 31 bp compared to that in 2b. Similarly, 3b showed 10 bp insertion compared to that in 3a. MZT1a germline DNA showed loss of 5 bp and 1b blood DNA showed loss of 26 bp compared to that of 1a blood and 1b germline DNA, respectively. Of the 69 restriction sites detected in DYZ1 arrays, *Mbo*II, *Bsr*I, *Tsp*EI and *Taq*I enzymes showed frequent loss and or gain amongst all the 3 pairs studied. MZT1 pair showed loss/gain of *Vsp*I, *Bsr*DI, *Ags*I, *Ple*I, *Tsp*DTI, *Tsp*EI, *Tfi*I and *Taq*I restriction sites in both blood and germline DNA. All the three sets of MZT showed differences in the number of DYZ1 copies. FISH signals reflected somatic mosaicism of the DYZ1 copies across the cells.

**Conclusions:**

DYZ1 showed both sequence and copy number variation between the MZT males. Sequence variation was also noticed between germline and blood DNA samples of the same individual as we observed at least in one set of sample. The result suggests that DYZ1 faithfully records all the genetical changes occurring after the twining which may be ascribed to the environmental factors.

## Background

The diverse role of nature and nurture has been addressed on the basis of studies on twins which are like natural clones. It is believed that the differences between twins are largely due to the influence of environmental factors. Theoretically, identical twins must be identical because they arise from a single fertilized egg (zygote). However, recent studies have shown that the identical twins are not truly identical as they show discernible variation in their genotypes [1-3]. The genetic differences between MZ twins represent an example of somatic mosaicism [4,5]. On the same token; one may expect similar mosaicism in the germline samples also. However, this has not been demonstrated unequivocally. During the early stages of life; it is difficult to uncover differences in any of the biological attributes of twins. However, as twins age, genetic and epigenetic changes accumulate which cause the differential expression of genes in twins [6,7]. Twins have been reported to show copy number variation for a number of genes [1]. Therefore, the term monozygotic twins (MZT) is more appropriate rather than the use of identical twins as there are no identical twins in true sense.

The reason of monozygotic twinning in human is not clear. MZ twins result when a fertilized egg or zygote splits into two embryos. This remarkable event takes place during the first week after fertilization and can happen at different times such as at the two cell stage on days 1 to 3, at the early blastocyst stage on days 4 to 6 or in the late blastocyst stage on days 7 to 9 [8]. The frequency of monozygotic twinning increases 2 to 5 times with *in vitro* fertilization [9,10]. In case of female monozygotic twinning, one suggested mechanism is the preferential inactivation of the normal X in one of the twins [11,12]. Twinning occurs spontaneously at the rate of about 1 in 80 live births [8,13]. However, monozygotic twinning spontaneously occurs at the rate of about 1 in 250 live births [8,14]. The rate of spontaneous twinning is highest (1 in 11) in Nigeria and lowest (1 in 250) in Japan. The occurrence is about 6 per 1000 in Asia, 10–20 per 1000 in Europe and USA and about 40 per 1000 in Africa [8,15].

Mammalian Y chromosome originated from an ancestral autosome about 300 million years ago is a degenerated X-chromosome [16]. The human Y chromosome is male specific, constitutively haploid and largely escapes meiotic recombination. Lack of recombination was thought to be responsible for the degeneration of the human Y chromosome and loss of Y linked genes, but a recent study showed that during the past 25 million years, the human Y chromosome lost only 1 gene [17]. Thus, crucial genes seem to have been retained by the Y chromosome.

Approximately, 95% (60 Mb) of the human Y chromosome represents a male specific region of the Y (MSY). Similarly, 5% (3 Mb) of the human Y chromosome comprises of pseudo-autosomal region (PAR) necessary for the pairing with the human X chromosome. The human Y chromosome has a high proportion of repeat elements. The satellite sequence DYZ1 constitutes approximately 20% of the total Y chromosome [18]. Based on the *Hae*III digestion of the human genomic DNA, DYZ1 was identified as a 3.4 Kb band in the males [19], which was found to largely contain a pentameric repeat ‘’TTCCA” [20]. A normal human Y chromosome contains approximately 3000–4300 copies of the DYZ1 arrays [21]. Since DYZ1 copies do not participate in recombination, it was deduced to have no functional or evolutionary advantage [16]. However, even the most repetitive stretches of DNA have significance in the genome as the same are envisaged to absorb undue mutational load. DYZ1 is now reported to play a crucial role in chromatin folding and maintenance of the structural integrity of the Y chromosome, thus having some functional attributes [21].

The major part of human genome is heterochromatic and environmentally triggered genomic changes are generally absorbed by this region. It is largely expected that, no major change takes place in the arrays of DYZ1 because it does not undergo recombination. However, Since DYZ1 represents heterochromatic region of the human Y chromosome, any change taking place between the two males of MZT after twining may in principle be detected. Mutations occurred during pre-twinning stage will be present in both the twins while, the ones acquired during the later stages in life will differentiate them from each other. With this premise, we undertook analysis of DYZ1 between the males of three sets of MZT. In one set, we analysed DYZ1 arrays in the DNA from the semen sample as well. In the present study blood DNA samples from three pairs of MZ twins were used. We also collected germline DNA samples from MZT1. We sequenced and virtually restriction mapped the 3564 bp unit of DYZ1 arrays. We also calculated the copy number of DYZ1 amongst the sets of these twins using Real Time PCR. The number of “TTCCA” repeats and its single, double, triple, four and five base pair derivatives per 3564 bp unit generate a profile for the respective arrays. Difference in the number of TTCCA repeats and its derivatives, copy number variation of DYZ1 arrays and loss/gain of the restriction enzyme sites were compared to uncover differences between MZT males. Similarly, comparison was also made between the blood DNA and germline DNA of the MZT1. Our result shows that DYZ1 indeed is capable of faithfully recording the sequence variation following the process of twining. These changes may or may not be exclusively due to environment, such correlation is possible to establish. This information is envisaged to be useful in the context of biology, medicine and forensic cases.

## Results

### 3564 bp unit of DYZ1 array

**Figure 1:**
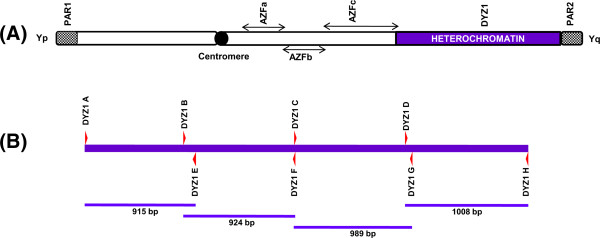
**Diagrammatic illustration showing PCR strategy for full length amplification of 3.56 Kb unit of DYZ1 array. (A)** Cartoon diagram of human Y chromosome with highlighted DYZ1 containing region in purple, **(B)** a single unit of DYZ1array is shown as a purple bar. Forward and reverse primers are shown above and below the bar. Similarly, amplification products and their corresponding sizes are shown below the bar. The amplified fragments were used for subsequent cloning and sequencing.

Four PCR amplified fragments (Figure 1, purple) were cloned and several positive clones were sequenced. We have taken the consensus of these sequences following the alignment as representative of the majority of the DYZ1 arrays. Further, we sequenced PCR amplified products directly and repeated the process twice and got variations each time. Keeping that in mind, we have relied on the sequences obtained from a cloned product over to that of amplified ones since cloned fragment ensures purity of the template having identical molecules. We have submitted sequences of all such cloned fragments to the GenBank and assigned accession numbers are given herein (MZT_1a Blood [KF941192], MZT_1a Germline [KF941193], MZT_1b Blood [KF941194], MZT_1b Germline [KF941195], MZT_2a [KF941196], MZT_2b [KF941197], MZT_3a [KF941198] and MZT_3b [KF941199]. The number of highly abundant “TTCCA” repeats and its single, double, triple, four and five base pair derivatives per 3564 bp unit (*Hae*III fragment) of DYZ1 array were counted. The differences in the number of TTCCA repeat unit and its derivatives between MZT are highlighted in **yellow, green and sky blue for MZT pairs 1, 2 and 3, respectively** (Table 1). Germline samples of both the individuals of MZ twin pair 1 were used. We also sequenced the 3.56 kb *Hae*III fragment of DYZ1 array originating from germline DNA of MZT1 pair, ascertained the number of TTCCA repeats and its derivatives per 3.56 kb unit of DYZ1 array and compared the differences between DNA of blood and germline origin with respect to DYZ1 array. The detailed result is shown in Table 2 and differences are highlighted in bold. DYZ1 array sequences with adjusted “TTCCA” reading frame are shown in Additional file 1.

**Table 1:** Number of “TTCCA” repeats and its single base pair derivatives along with double, triple, four and five base pair derivatives per 3.56 Kb unit of DYZ1 array are shown for all 3 MZT pairs

**Sr. no.**	**Repeat unit and derivatives**	**Number of TTCCA repeat units and its derivatives per 3564 bp *Hae*** **III unit of DYZ1 array**					
		**MZT1a**	**MZT1b**	**MZT2a**	**MZT2b**	**MZT3a**	**MZT3b**
1	TTCCA	**235**	**226**	**221**	**228**	**184**	**190**
2	1 bp derivatives	**290**	**289**	**286**	**295**	**245**	**238**
	ATCCA	9	9	9	9	7	7
	TACCA	3	3	3	3	2	2
	TTACA	16	16	**15**	**16**	**12**	**11**
	TTCAA	21	21	**22**	**23**	**21**	**19**
	TTTCA	19	19	**18**	**19**	**17**	**15**
	TTCTA	**25**	**26**	**24**	**25**	**24**	**23**
	TTCCT	33	33	**34**	**30**	**29**	**27**
	GTCCA	29	29	**30**	**31**	26	26
	TGCCA	**10**	**9**	9	9	8	8
	TTGCA	**23**	**22**	**24**	**25**	**16**	**18**
	TTCGA	60	60	**55**	**62**	**50**	**51**
	TTCCG	17	17	**19**	**18**	14	14
	CTCCA	14	14	**12**	**14**	10	10
	TCCCA	4	4	4	4	2	2
	TTCCC	7	7	8	7	7	5
3	2 bp derivatives	**142**	**140**	**154**	**139**	**116**	**120**
4	3 bp derivatives	**33**	**36**	**32**	**37**	28	28
5	4 bp derivatives	**9**	**11**	**9**	**8**	**9**	**8**
6	5 bp derivatives	1	1	1	1	1	1

The differences are highlighted in bold.

**Table 2:** Number of “TTCCA” repeats and it’s single base pair derivatives along with double, triple, four and five base pair derivatives per 3.56 Kb unit of DYZ1 array are shown for MZT1

**Sr. no.**	**Repeat unit and derivatives**	**Number of “TTCCA” repeat units and its derivatives per 3.56 Kb unit of DYZ1 array**			
		**MZT1a**	**MZT1a**	**MZT1b**	**MZT1b**
		**(Blood DNA)**	**(Germline DNA)**	**(Blood DNA)**	**(Germline DNA)**
1	TTCCA	**235**	**234**	**226**	**232**
2	1 base pair derivatives	290	290	**289**	**288**
	ATCCA	**9**	**10**	9	9
	TACCA	3	3	**3**	**4**
	TTACA	**16**	**15**	16	16
	TTCAA	**21**	**22**	**21**	**22**
	TTTCA	19	19	**19**	**18**
	TTCTA	25	25	**26**	**25**
	TTCCT	**33**	**32**	**33**	**32**
	GTCCA	**29**	**30**	**29**	**30**
	TGCCA	10	10	**9**	**10**
	TTGCA	**23**	**22**	22	22
	TTCGA	60	60	60	60
	TTCCG	17	17	**17**	**16**
	CTCCA	14	14	14	14
	TCCCA	4	4	4	4
	TTCCC	7	7	**7**	**6**
3	2 base pair derivatives	142	142	140	140
4	3 base pair derivatives	33	33	**36**	**35**
5	4 base pair derivatives	9	9	**11**	**9**
6	5 base pair derivatives	1	1	1	1

The differences are highlighted in bold.

### Multiple sequence alignment of 3564 bp unit of DYZ1 array

DYZ1 sequences of all the three twin pairs were aligned in pair wise combination using ClustalW software. Further, DYZ1 array sequence originating from blood and germline DNA of both the individuals of MZT1 were aligned with each other. Sequence variations (point mutations, deletions or insertions) are highlighted in yellow (Additional files 2 and 3). MZT1b have shown deletions of 20 bp and 15 bp between 390-411 bp and 1728–1744 bp positions, respectively with net loss of 35 bp when compared to MZT1a. Similarly, MZT2a showed deletions of 16 bp and 15 bp between 411–427 bp and 765–781 bp positions, respectively, with net loss of 31 bps when compared to MZT2b. However, MZT1b itself has 5 bp less than the standard 3564 bp length. Further, MZT3b also showed deletion of 1 bp and 5 bp between 1068–1070 bp and 1580–1586 bp positions, respectively, as compared to MZT3a. MZT3b also showed insertion of 1 bp and 15 bp between 1668–1669 and 2515–2516 respectively, compared with MZT3a. So, MZT3b showed net insertion of 10 bp as compared to MZT3a. When blood and germline DYZ1 sequence of MZT1 were compared, germline sample of MZT1a showed deletion of 5 bp between 410–416 bp positions as compared with that of MZT1a blood DNA. Similarly, MZT1b blood DYZ1 sequence showed deletion of 15 bp each between 390–406 bp and 1722–1738 bp positions and insertion of 1 bp between 844–845 bp, 3487–3488 bp, 3518–3519 bp and 3540–3549 bp positions as compared to MZT1b germline DYZ1 sequence. Thus, DYZ1 from MZT1b blood showed net loss of 26 bps.

### Restriction mapping of 3564 bp *Hae*III fragment of DYZ1 array

A 3564 bp unit of DYZ1 array was subjected to virtual restriction mapping for all the three sets of twins (Table 3). Twin pair 1 showed single loss/gain of *Mbo*II, *Bst*XI, *Bsr*DI, *Dsr*I, *Bsm*I and TaqI sites. Likewise, CspCI and *Tsp*EI showed loss/gain of these restriction sites twice. Twin pair 2 showed single loss/gain of *Dde*I, *Bse*MII, *Tat*I, *Mbo*II, *Bcc*I, *Sfa*NI, *Tst*I, *Mse*I, *Bsr*I, *Ags*I, *Ple*I, *Tsp*DTI, *Bsa*BI, *Nla*IV, *Btg*ZI, *Mae*II and double loss/gain of *Ars*I, *Alf*I, *Nla*III, *Bcg*I, *Bsr*DI, *Tsp*GWI, *Fai*I, *Tsp*EI, *Hin*FI, *Taq*II, *Mfe*I restriction sites while, *Tfi*I and *Taq*I showed loss/gain of three and six restriction sites, respectively. In twin pair 3, single loss/gain of *Cac*8I, *Eco*RI, *Set*I, *Asu*II, *Mbo*II, *Bcc*I, *Mae*I, *Rsa*I, *Xmn*I, *Nla*III, *Fai*I, *Ple*I, *Tsp*DTI, *Taq*I, *Hin*FI, *Btg*ZI, *Mae*III, *Tsp*45I, *Hph*I, *Nsp*I and *Sph*I restriction site were detected. Similarly, *Apo*I, *Bcg*I and *Bsr*I showed loss/gain of two restriction sites. A single enzyme *Tsp*EI showed loss of three sites. Out of 69 restriction enzymes sites detected in DYZ1, *Mbo*II, *Bsr*I, *Tsp*EI and *Taq*I showed frequent loss or gain of the restriction sites in all the three twin pairs studied. The germline DNA samples studied along with the blood DNA samples for MZT1a showed single loss/gain of *Vsp*I, *Set*I, *Asu*II, *Bsr*DI, *Bsm*I, *Ags*I, *Ple*I, *Tsp*DTI, *Tsp*EI, *Tfi*I and *Taq*I restriction sites. Likewise, *Csp*CI and *Bcg*I showed double loss/gain of the restriction sites. Further, MZT 1b showed single loss/gain of *Vsp*I, *Acy*I, *Hga*I, *Hpy*99I, *Dpn*I, *Fok*I, *Mbo*I, *Tsp*RI, *Sfa*NI, *Bst*XI, *Bsr*DI, *Bsr*I, *Ags*I, *Fai*I, *Ple*I, *Tsp*DTI, *Taq*I and *Hinf*I, double loss/gain of *Hae*IV and *Hin*4I and triple loss/gain of *Tsp*EI restriction sites. Out of 63 restriction sites detected in blood and germline DNA samples of MZT1, *Vsp*I ,*Bsr*DI, *Ags*I, *Ple*I, *Tsp*DTI, *Tsp*EI, *Tfi*I and *Taq*I showed loss/gain of restriction sites in both the individuals of MZT1. Detailed result of virtual restriction mapping for blood and germline DNA sample of MZT1 is given in the Table 4.

**Table 3:** Virtual restriction mapping of 3.56 Kb unit of DYZ1 array from twin pairs (blood DNA) using restriction mapper software

**Sr. no.**	**Restriction site**	**Sequence**	**Overhang**	**Restriction site frequency**					
				**MZT1a**	**MZT1b**	**MZT2a**	**MZT2b**	**MZT3a**	**MZT3b**
1	*Cac*8I	GCNNGC	5’	1	1	1	1	**2**	**1**
2	*Bse*YI	CCCAGC	5’	1	1	1	1	1	1
3	*Bsp*1407I	TGTACA	5’	1	1	1	1	0	0
4	*Bsp*HI	TCATGA	3’	1	1	1	1	0	0
5	*Cla*I	ATCGAT	3’	1	1	1	1	1	1
6	*Dde*I	CTNAG	blunt	1	1	**0**	**1**	0	0
7	*Eco*RI	GAATTC	5’	1	1	1	1	**2**	**1**
8	*Scr*FI	CCNGG	3’	1	1	1	1	1	1
9	*Vsp*I	ATTAAT	blunt	1	1	1	1	1	1
10	*Bse*MII	CTCAG	5’	1	1	**0**	**1**	0	0
11	*Bsg*I	GTGCAG	3’	1	1	1	1	0	0
12	*Bts*I	GCAGTG	3’	1	1	1	1	0	0
13	*Eco*57I	CTGAAG	3’	1	1	1	1	1	1
14	*Gsu*I	CTGGAG	3’	1	1	1	1	0	0
15	*Hpy*188I	TCNGA	3’	1	1	1	1	0	0
16	*Mn*lI	CCTC	3’	1	1	2	2	0	0
17	*Pfl*MI	CCANNNNNTGG	3’	1	1	1	1	1	1
18	*Sdu*I	GDGCHC	blunt	1	1	1	1	0	0
19	*Bsa*BI	GATNNNNATC	blunt	0	0	1	0	0	0
20	*Taq*II	GACCGA	3’	0	0	**2**	**0**	0	0
21	*Mfe*I	CAATTG	5’	0	0	**0**	**2**	0	0
22	*Dpn*I	GATC	5’	2	2	1	1	1	1
23	*Fok*I	GGATG	3’	2	2	2	2	2	2
24	*Mbo*I	GATC	5’	2	2	1	1	1	1
25	*Ars*I	GACNNNNNNTTYG	5’	2	2	**0**	**2**	2	2
26	*Bda*I	TGANNNNNNTCA	5’	2	2	2	2	2	2
27	*Csp*CI	CAANNNNNGTGG	5’	**2**	**0**	0	0	0	0
28	*Eco*57MI	CTGRAG	5’	2	2	2	2	1	1
29	*Set*I	ASST	5’	2	2	2	2	**2**	**1**
30	*Tsp*RI	CASTG	5’	2	2	2	2	1	1
31	*Apo*I	RAATTY	3’	3	3	3	3	**4**	**2**
32	*Asu*II	TTCGAA	3’	3	3	3	3	**3**	**4**
33	*Eco*31I	GGTCTC	blunt	3	3	3	3	3	3
34	*Tat*I	WGTACW	5’	3	3	**3**	**4**	1	1
35	*Mbo*II	GAAGA	3’	**3**	**4**	**4**	**3**	**3**	**4**
36	*Cvi*JI	RGCY	blunt	4	4	4	4	3	3
37	*Ms*lI	CAYNNNNRTG	5’	4	4	4	4	3	3
38	*Bcc*I	CCATC	3’	4	4	**5**	**4**	**4**	**5**
39	*Mae*I	CTAG	3’	4	4	4	4	**3**	**4**
40	*Sfa*NI	GCATC	3’	4	4	**3**	**4**	2	2
41	*Alf*I	GCANNNNNNTGC	3’	4	4	**2**	**4**	2	2
42	*Tst*I	CACNNNNNNTCC	3’	4	4	**4**	**5**	4	4
43	*Rsa*I	GTAC	3’	5	5	5	5	**2**	**3**
44	*Bsm*AI	GTCTC	3’	5	5	5	5	5	5
45	*Bst*XI	CCANNNNNNTGG	blunt	**5**	**4**	5	5	5	5
46	*Xmn*I	GAANNNNTTC	5’	6	6	6	6	**5**	**6**
47	*Mse*I	TTAA	3’	6	6	**6**	**5**	6	6
48	*Nla*III	CATG	5’	7	7	**9**	**7**	**5**	**4**
49	*Bcg*I	CGANNNNNNTGC	5’	14	14	**18**	**16**	**10**	**12**
50	*Bsr*DI	GCAATG	5’	**15**	**14**	**14**	**16**	11	11
51	*Bsr*I	ACTGG	5’	**19**	**18**	**19**	**18**	**12**	**14**
52	*Tsp*GWI	ACGGA	5’	19	19	**21**	**19**	16	16
53	*Bsm*I	GAATGC	5’	**21**	**20**	22	22	**13**	**17**
54	*Ags*I	TTSAA	5’	22	22	**24**	**25**	**24**	**20**
55	*Fai*I	YATR	3’	23	23	**25**	**23**	**17**	**16**
56	*Ple*I	GAGTC	3’	23	23	**24**	**23**	**19**	**18**
57	*Tsp*DTI	ATGAA	blunt	25	25	**23**	**24**	**18**	**17**
58	*Tsp*EI	AATT	5’	**34**	**32**	**36**	**34**	**32**	**29**
59	*Tfi*I	GAWTC	3’	53	53	**51**	**54**	48	48
60	*Taq*I	TCGA	blunt	**66**	**67**	**61**	**67**	**54**	**55**
61	*Hinf*I	GANTC	5’	76	76	**75**	77	**67**	**66**

The differences are highlighted in bold.

**Table 4:** Virtual restriction mapping of 3.56 kb unit of DYZ1 array from MZT1 (both blood and germline DNA) using Restriction Mapper software

**Sr. No.**	**Restriction Site**	**Sequence**	**Overhang**	**Restriction Enzyme Site Frequency**			
				**MZT1a (Blood)**	**MZT1a (Germline)**	**MZT1b (Blood)**	**MZT1b (Germline)**
1	*Cac*8I	GCNNGC	5’	1	1	1	1
2	*Bse*YI	CCCAGC	5’	1	1	1	1
3	*Bsp*1407I	TGTACA	5’	1	1	1	1
4	*Bsp*HI	TCATGA	3’	1	1	1	1
5	*Cla*I	ATCGAT	3’	1	1	1	1
6	*Dde*I	CTNAG	blunt	1	1	1	1
7	*Eco*RI	GAATTC	5’	1	1	1	1
8	*Scr*FI	CCNGG	3’	1	1	1	1
9	*Vsp*I	ATTAAT	blunt	**1**	**2**	**1**	**2**
10	*Bse*MII	CTCAG	5’	1	1	1	1
11	*Bsg*I	GTGCAG	3’	1	1	1	1
12	*Bts*I	GCAGTG	3’	1	1	1	1
13	*Eco*57I	CTGAAG	3’	1	1	1	1
14	*Gsu*I	CTGGAG	3’	1	1	1	1
15	*Hpy*188I	TCNGA	3’	1	1	1	1
16	*Mn*lI	CCTC	3’	1	1	1	1
17	*Pfl*MI	CCANNNNNTGG	3’	1	1	1	1
18	*Sdu*I	GDGCHC	blunt	1	1	1	1
19	*Acy*I	GRCGYC	5’	0	0	**0**	**1**
20	*Hga*I	GACGC	5’	0	0	**0**	**1**
21	*Hpy*99I	CGWCG	3’	0	0	**0**	**1**
22	*Hae*IV	GAYNNNNNRTC	3’	0	0	**0**	**2**
23	*Hin*4I	GAYNNNNNVTC	3’	0	0	**0**	**2**
24	*Dpn*I	GATC	5’	2	2	**2**	**3**
25	*Fok*I	GGATG	3’	2	2	**2**	**3**
26	*Mbo*I	GATC	5’	2	2	**2**	**3**
27	*Ars*I	GACNNNNNNTTYG	5’	2	2	2	2
28	*Bda*I	TGANNNNNNTCA	5’	2	2	2	2
29	*Csp*CI	CAANNNNNGTGG	5’	**2**	**0**	0	0
30	*Eco*57MI	CTGRAG	5’	2	2	2	2
31	*Set*I	ASST	5’	**2**	**3**	2	2
32	*Tsp*RI	CASTG	5’	2	2	**2**	**3**
33	*Apo*I	RAATTY	3’	3	3	3	3
34	*Asu*II	TTCGAA	3’	**3**	**4**	3	3
35	*Eco*31I	GGTCTC	blunt	3	3	3	3
36	*Tat*I	WGTACW	5’	3	3	3	3
37	*Mbo*II	GAAGA	3’	3	3	4	4
38	*Cvi*JI	RGCY	blunt	4	4	4	4
39	*Ms*lI	CAYNNNNRTG	5’	4	4	4	4
40	*Bcc*I	CCATC	3’	4	4	4	4
41	*Mae*I	CTAG	3’	4	4	4	3
42	*Sfa*NI	GCATC	3’	4	4	**4**	**5**
43	*Alf*I	GCANNNNNNTGC	3’	4	4	4	4
44	*Tst*I	CACNNNNNNTCC	3’	4	4	4	4
45	*Rsa*I	GTAC	3’	5	5	5	5
46	*Bsm*AI	GTCTC	3’	5	5	5	5
47	*Bst*XI	CCANNNNNNTGG	blunt	5	5	**4**	**5**
48	*Xmn*I	GAANNNNTTC	5’	6	6	6	6
49	*Mse*I	TTAA	3’	6	6	6	6
50	*Nla*III	CATG	5’	7	7	7	7
51	*Bcg*I	CGANNNNNNTGC	5’	**14**	**12**	14	14
52	*Bsr*DI	GCAATG	5’	**15**	**14**	**14**	**15**
53	*Bsr*I	ACTGG	5’	19	19	**18**	**19**
54	*Tsp*GWI	ACGGA	5’	19	19	19	19
55	*Bsm*I	GAATGC	5’	**21**	**20**	20	20
56	*Ags*I	TTSAA	5’	**22**	**23**	**22**	**23**
57	*Fai*I	YATR	3’	23	23	**23**	**24**
58	*Ple*I	GAGTC	3’	**23**	**24**	**23**	**24**
59	*Tsp*DTI	ATGAA	blunt	**25**	**24**	**25**	**24**
60	*Tsp*EI	AATT	5’	**34**	**35**	**32**	**35**
61	*Tfi*I	GAWTC	3’	**53**	**52**	**53**	**51**
62	*Taq*I	TCGA	blunt	**66**	**65**	**67**	**66**
63	*Hinf*I	GANTC	5’	76	76	**76**	**75**

The germline samples for remaining two twin pairs (MZT2 and 3) were not available. The differences in restriction site frequency are highlighted in bold.

**Figure 2:**
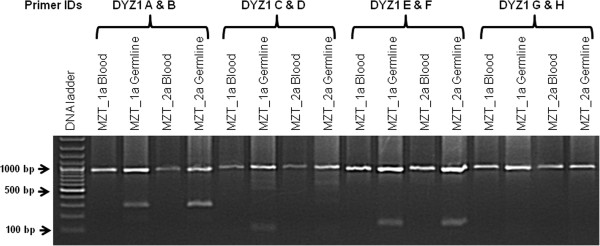
**A representative gel picture showing Restriction Fragment Length Polymorphism (RFLP) in monozygotic males: *Dpn*I is used for restriction digestion.** The DYZ1 fragments generated using primers DYZ1 A & B, C & D and E & F show presence of restriction site in germline samples compared to that in blood. However, DYZ1 fragment amplified by primers DYZ1 G & H does not contain any *Dpn*I site both in blood and germline DNA.

The real restriction mapping experiments did not always correlate with the virtual restriction mapping data because in case of virtual restriction mapping, we dealt with a single array sequence while in case of real restriction mapping, we dealt with a pool of DYZ1 array sequences and average of all of them may be lot more different than that of a single array sequence. Out of several restriction enzymes like *Rsa*I, *Bst*XI, *Dpn*I, *Eco*RI, *Mbo*I, *Mbo*II, *Xmn*I, *Tat*I, *Mse*I, *Apo*I, *Mfe*I, *Bse*MII, *Nla*III and *Dde*I; *Dpn*I restriction pattern showed variation between blood and germline DNA (Figure 2). Taken together, the blood genomic DNA does not contain *Dpn*I site while germline DNA does in MZT1.

### DYZ1 copy number variation

**Figure 3:**
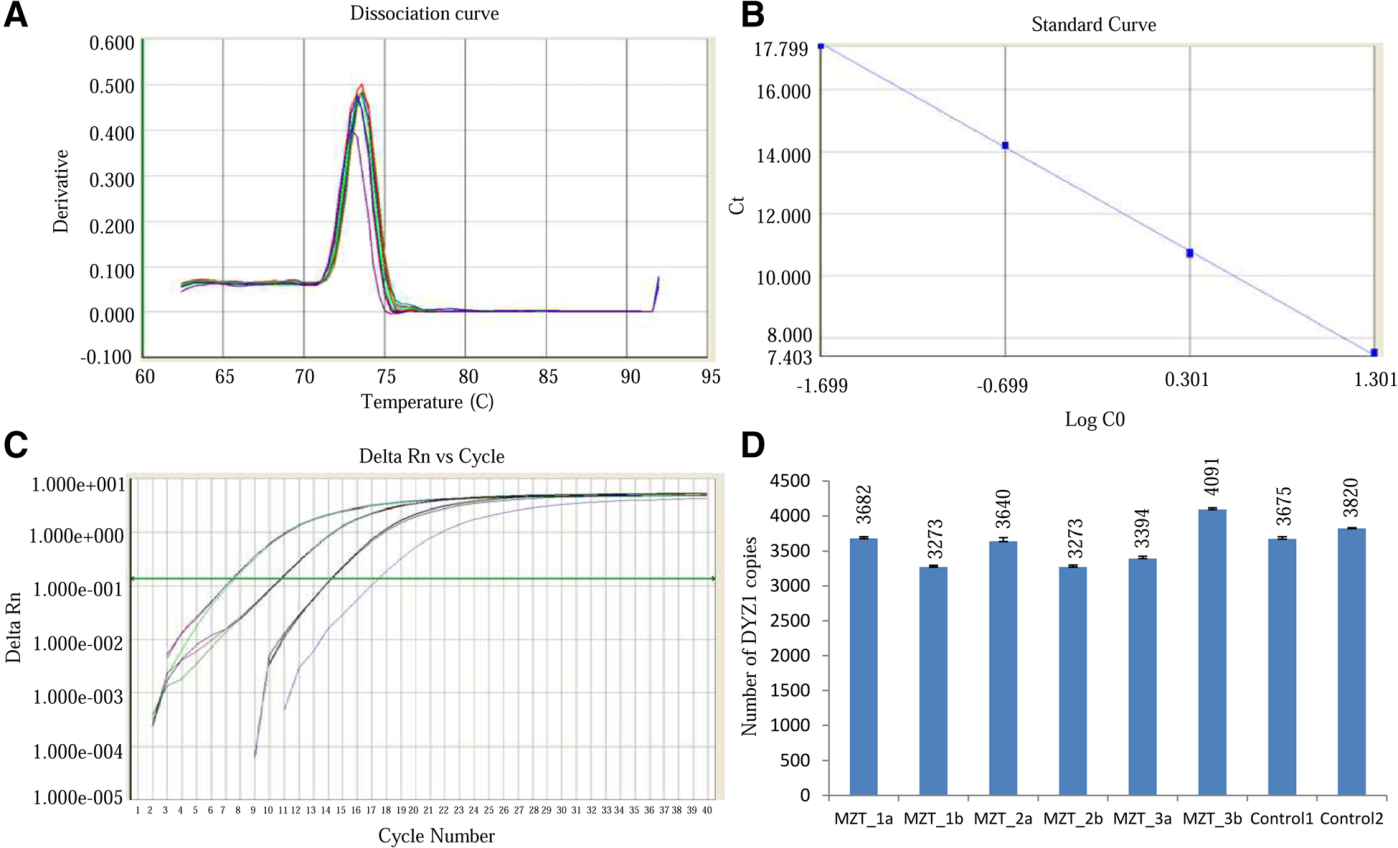
**Copy number estimation of DYZ1 in Monozygotic twin pairs. (A)** represents the dissociation curve, **(B)** the standard plot **(C)** the amplification plot and **(D)** shows the number of DYZ1 copies in all three twin pairs along with two control samples.

DYZ1 copy number was calculated using absolute quantitative PCR following SYBR green chemistry and a standard curve of cloned DYZ1 plasmid using ten-fold dilutions. The dissociation curve, standard curve and amplification plot are given in Figures 3A,B and C, respectively. The respective copy number values for all twin pairs and controls are shown in figures 3D. Twin pair sets 1, 2 and 3 showed differences of 409, 367 and 697 of DYZ1 copies, respectively.

### Localization of DYZ1 on metaphases/nuclei using Fluorescence *in situ* Hybridization

**Figure 4:**
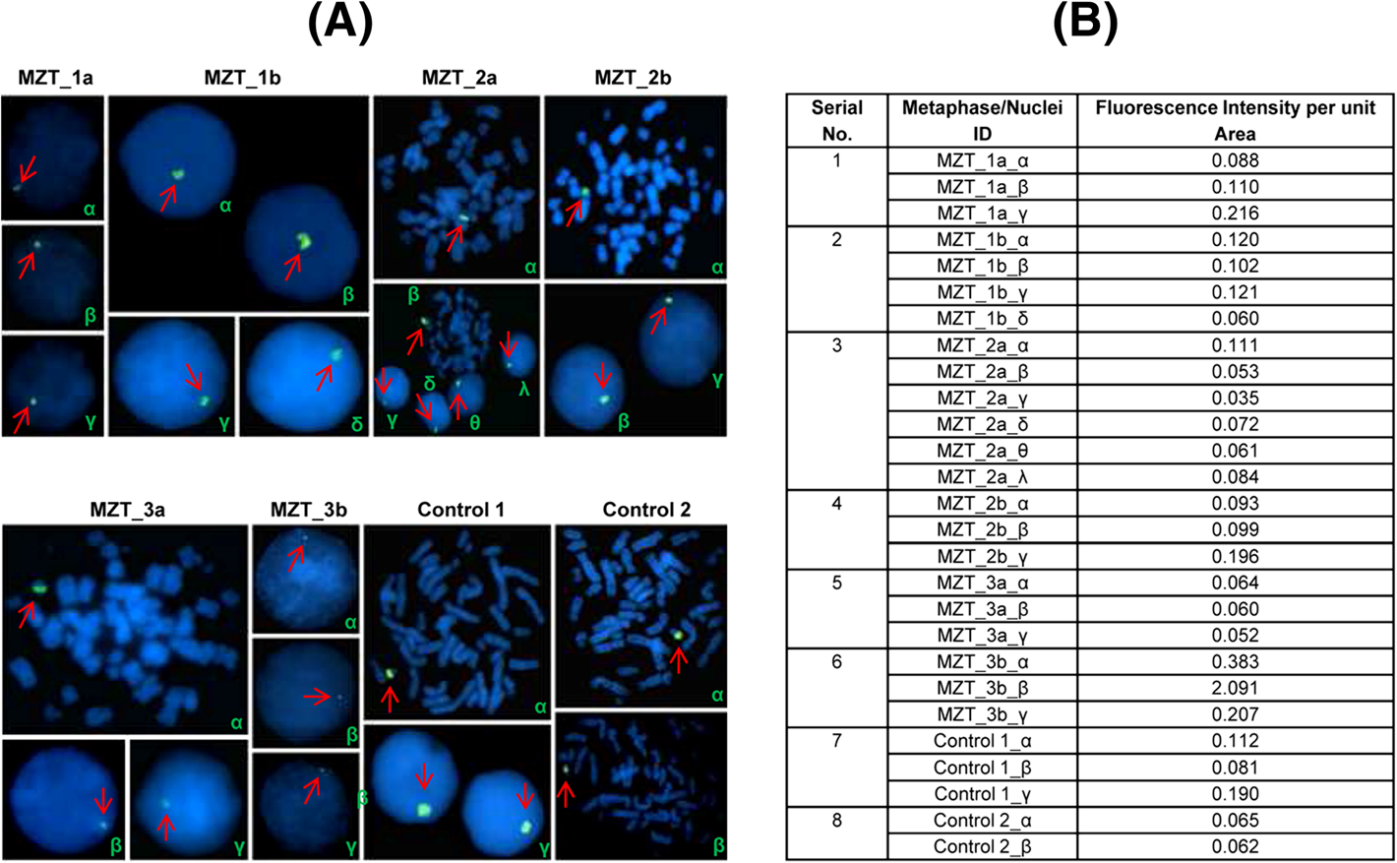
**Localization of DYZ1 using FISH. (A)** DAPI (4′, 6-diamidino-2-phenylindole) stained metaphases and interphase nuclei are shown having green signal of DYZ1 probe by red arrows. **(B)** The table shows fluorescence intensity per unit area values for each DYZ1 probe signal spot. Note the variation in the DYZ1 probe’s signal intensities across nuclei reflecting copy number variation and somatic mosaicism.

We screened approximately 400 nuclei and metaphases. To rule out the possibility of experimental error, two positive controls (metaphases prepared from normal human blood) were used with the same probe preparation. Following FISH, the nuclei and metaphases showed DYZ1 signal of varying intensity which is due to the varying number of its copies. The representative FISH pictures are shown in Figure 4.

## Discussion

The genome that we are born with is not the one that we die with [1]. This is true for all the cells in our body. So, as we age, environmentally triggered genomic changes accumulate in our DNA more in the repeat regions. Accordingly then, the difference between the identical twins increases as they age. Twins can also begin their lives with some major differences.

MSY region of the human Y chromosome does not take part in the crossing over, so the DNA comprising MSY is faithfully passed on from father to son. However, MSY may accumulate mutations during the life time of an individual. In case of DYZ1, point mutations generate derivatives of “TTCCA” while insertions and deletions shift the “TTCCA” frame. Genome tries to neutralize or minimize these changes. In the process, insertion at one point may lead to the deletion at another point and vice versa (Additional file 2). Despite these changes in the number of “TTCCA” and its derivatives, the overall length of the array remains almost unchanged. Independent mutational events may also lead to gain or loss of restriction enzyme sites in DYZ1 array which is evident from the present study.

In addition to these, DYZ1 arrays showed copy number variation between MZT as uncovered by real time PCR. However, fluorescence signal intensity (Figure 4) of DYZ1 probe is not always correlated with its copy number variation. This is because every cell does not contain equal number of DYZ1 copies. Similarly, DNA used for quantitative Real Time PCR does not contain homogeneous population of DYZ1 sequences. Thus, DYZ1 copies calculated using absolute quantification is the average of the DYZ1 arrays present in the pool of DNA from all the cells.

Analysis of DYZ1 has been pursued in our laboratory in the context with Sex Chromosome Related Anomalies (SCRA) [22], males exposed to Natural Background Radiation (NBR) [21], Arsenic Poisoning [23], Prostate Cancer cell lines [18] and Infertility [24]. Significantly, DYZ1 was found to show much reduced copies in all these cases. Thus, indeed there exists a correlation between the reduced copies of the DYZ1 and these abnormal conditions. DYZ1 does not unequivocally differentiate between monozygotic twins but the effect of nature vs. nurture on twins can be studied with respect to DYZ1 arrays. This is because at the timing of twining, the copies in both the males are expected to be identical. Any variation noticed either in the copies of the arrays or within is ascribable to the environmental conditions. Thus, present study has relevance in the context of changes brought about in the DYZ1 arrays between two males of monozygotic origin.

Taken together, the study mainly supports the argument that, the monozygotic twins are not really identical as evident from this study. Extrapolation of this study in a large number of samples may lead to the discovery of sufficient genetic variation in the DYZ1 arrays from across the samples. This in turn would augment the already existing approaches useful for the discrimination of identical twins in the context of forensic cases.

## Conclusions

DYZ1 arrays have shown variations between the monozygotic males. Similarly, sequence variations were also established between germline and blood DNA samples of the same individual for one twin pair. This approach is envisaged to be of relevance in biology, medicine and forensic cases if sufficiently large number of samples both from blood and germline are analysed.

## Methods

### Sample collection and DNA isolation

Present study was approved by the Institutional Human Ethical Committee of the National Institute of Immunology, New Delhi. Peripheral blood lymphocytes (PBLs) were collected from three pairs of male monozygotic twins, with their informed consent. Genomic DNA from blood was isolated using DNeasy Blood and Tissue kit from Qiagen, Germany (Cat no. 29504) and Germline DNA of MZT1 was isolated following standard protocol [21]. Quality of isolated DNA was checked by electrophoresis using 1% agarose gel. DNA concentration was measured spectrophotometrically.

### End point PCR

PCR primers used to amplify the full 3.56 bp *Hae*III fragment of DYZ1 are listed in Table 5 and illustrated graphically in Figure 1. The end point PCR reactions were carried out in 20 μl volume containing Pfu DNA polymerase (Biotools, Spain), 5X reaction buffer (Promega, Madison, USA), 200 μM dNTPs (Bio. Basic Inc. Toronto, Canada) and 100 ng template DNA. The amplified products were resolved on 1.0% agarose gels, stained with Ethidium bromide and visualized under Ultraviolet.

**Table 5:** List of primers used for PCR amplification of DYZ1 array

**Serial no.**	**Primer ID**	**Primer sequence**	**Length (bp)**	**Location**	**Orientation**
**1**	DYZ1 **A**	CCATTCGAGACCGTAGCAATT	21	35-16 (5’ upstream to HaeIII site)	5’-3’
**3**	DYZ1 **B**	ATTTGATGCCATCCCATGAC	20	763-782	5’-3’
**5**	DYZ1 **C**	TCCTTTGCCTTCCATTCG	18	1668-1685	5’-3’
**6**	DYZ1 **D**	TGCAGTCTTTTCCCTTCGAG	20	2564-2583	5’-3’
**7**	DYZ1 **E**	ATTGGATGGGATTGGAATGA	20	861-880	3’-5’
**9**	DYZ1 **F**	TCGAATGGAAGGCAAAGG	18	1669-1686	3’-5’
**10**	DYZ1 **G**	CGACTGGTACGGACTCCAT	20	2637-2656	3’-5’
**12**	DYZ1 **H**	TGGACAGCCTGGAATAAAGTG	21	3586-3606	3’-5’

### Cloning and DNA sequencing

End point PCR amplified DYZ1 array fragments resolved on 1.0% agarose gel were extracted using a kit (Fermentas, Thermo Fischer Scientific). Purified fragments of DNA were cloned in blunt end cloning vectors (CloneJet, Fermentas, Thermo Fischer Scientific). Four recombinant clones, each representing positive ones were selected after conducting colony PCR using vector specific forward and reverse primers. Recombinant clones were further confirmed by restriction digestion. Four purified recombinant clones were sequenced on Applied Biosystems 3130xl genetic analyzer using ABI ABIPRISM® BigDye® terminator v3.1 cycle sequencing kits (Life technologies, California, USA). PCR conditions were set as 96°C for 1 minute, followed by 25 cycles each consisting of 96°C for 10 seconds, 50°C for 5 seconds, and 60°C for 4 minutes. After cycle sequencing, extension products were purified to remove any unincorporated dye-labelled terminators using ethanol–sodium acetate precipitation method followed by washing in 70% ethanol. Hi-Di™ Formamide (Life technologies, California, USA) was added, samples were heat denatured, chilled on ice and loaded onto the 3130xl genetic analyzer, ABI. The data was collected using 3130xl Data Collection Software v3.0. Sequences were analyzed using Sequence Scanner software version 1.0 and gene runner software version 3.05.

### Restriction mapping

The DYZ1 sequences were subjected to virtual restriction mapping using Restriction Mapper Software Version 3.0 (Tables 3 and 4). To support the virtual restriction mapping data, we conducted real restriction mapping on PCR amplified product using several restriction enzymes. The reaction digestions were carried out in 20 μl reaction mixture using 1 μg of template DNA and 2 units of enzyme following standard protocols (NEB, UK). Digested samples were resolved on 2.0% agarose gel and visualized under UV illumination to record the resultant bands.

### Copy number estimation

Number of DYZ1 copies was calculated based on absolute quantification method using quantitative PCR (qPCR). DNA was used as template and SYBR green (Life Technologies, California, USA) as detection dye. The qPCR reactions were performed on Sequence Detection System 7500 (Life Technologies, California, USA) following 10 fold dilutions of recombinant plasmid containing ~3.4 Kb *Hae*III fragment of DYZ1 array starting with 2 × 10^8^ copies and standard curve were generated. All the reactions were carried out in triplicates using three different concentrations of the template DNA. The standard curve has a slope of -3.32 and R^2^ value of >0.99. Copies of the DYZ1 array were calculated by extrapolation of the standard curve obtained with known copies of the recombinant plasmid. To show the reproducibility of qPCR results, error bars are shown on top of the graph bars.

### Florescence *in-situ* Hybridization (FISH)

Peripheral blood cells cultured in PB-MAX™ Karyotyping Medium (Gibco®, USA) were used for metaphase chromosome preparation. The cells were grown for 70 hours in 5% CO_2_ environment at 37°C and then treated with colcemid (3 μg/ml). Treated cells were again incubated for 2 hours in 5% CO_2_ environment at 37°C. After 72 hours, cells were centrifuged at 1800 rpm for 10 minutes at room temperature (RT). Harvested cells were resuspended in 0.075 M KCl and incubated at RT for 20 minutes in 5% CO_2_ environment at 37°C. Then added 1 drop of fixative solution (3:1, methanol: glacial acetic acid) and centrifuged at 1800 rpm for 10 minutes at RT. Discarded the supernatant, resuspended the cell pellet in 10 ml fresh fixative solution and incubated for 20 minutes at 37°C. Then centrifuged cells at 1800 rpm for 10 minutes at RT. Repeated the washing step 2 times. Finally, cells were resuspended in fresh 1 ml fixative and stored at -20°C until used.

20 μl of nuclei suspension in fixative was spread on the fixative dipped glass slides. Before proceeding further, slides were kept for 1 week at 37°C for ageing. Slides were then incubated in 70% glacial acetic acid for 2 minutes followed by dehydration in 70%, 90% and 100% ethanol for 2 minutes each at RT. Slides were air dried and incubated in a solution containing 0.1 mg/ml and 0.01 N HCl for 20 minutes. Fixed the metaphase preparation in 4% paraformaldehyde (prepared in 1X PBS, pH 7.4) for 5 minutes at RT. Slides were washed 2 times in PBS followed by once in water. Further, slides were dehydrated in 70%, 90% and 100% ethanol sequentially. Air dried slides were then used for hybridization. FISH was conducted with a labelled clone containing 3.56 kb sequence of DYZ1 array. Labelling was done with biotin-dUTP using a Nick translation kit from Vysis (Illinois, USA). Hybridization, washing, counterstaining and mounting of the slides were conducted following standard protocols [25]. The slides were screened under the Olympus fluorescence microscope (BX 51) fitted with vertical fluorescence illuminator U-LH100HG UV, excitation and barrier filters and images were captured with a charge-coupled device (CCD) camera. Captured images were analysed using CytoVision software version 3.93 from Applied Imaging Systems.

## Abbreviations

MZT: Monozygotic twin; NRY: Non recombining region of Y; PAR: Pseudo autosomal region; MSY: Male specific region of Y; DNA: Deoxyribonucleic acid; PBLs: Peripheral blood lymphocytes; PCR: Polymerase chain reaction; RFLP: Restriction fragment length polymorphism; UV: Ultraviolet; qPCR: Quantitative polymerase chain reaction; FISH: Fluorescence *in situ* hybridization; MSA: Multiple sequence alignment.

## Competing interests

The authors declare no competing interests.

## Authors’ contributions

SKY and SA conceived the study. SKY and AK carried out the experiments and did *in-silico* analysis. SKY and SA interpreted the data and wrote the manuscript. SJ provided the twin sample MZT2. MZT1 and 3 were arranged by SKY. All the authors read and approved the final version of the manuscript.

## Supplementary Material

Additional file 1 **3.56 Kb sequence of DYZ1 array from all three twin pairs, in adjusted frame of “TTCCA”.** (A) MZT_1a (Blood), MZT_1a (Germline), MZT_1b (Blood) and MZT_1b (Germline); (B) MZT_2a (Blood) and MZT_2b (Blood) and (C) MZT_3a (blood) and MZT_3b (Blood). Click here for file

Additional file 2 **Multiple sequence alignment (MSA) of 3.56 Kb sequence of DYZ1 array from twin pairs.** The regions of nucleotide variations are highlighted in yellow. (A) MZT1, (B) MZT2 and (C) MZT3. Click here for file

Additional file 3 **Multiple Sequence Alignment (MSA) of 3.56 Kb sequence of DYZ1 array from Blood DNA with Germline DNA of MZT1.** The insertions, deletions and point mutations are highlighted in yellow. (A) MZT1a and (B) MZT1b. Click here for file
