# Supplementary material for: DYZ1 arrays show sequence variation between the monozygotic males
Source: BMC Genet. 2014 Feb 4;15:19. doi: 10.1186/1471-2156-15-19 (PMC3925983; doi:10.1186/1471-2156-15-19)
Supplement: Additional file 1 — 3.56 Kb sequence of DYZ1 array from all three twin pairs, in adjusted frame of “TTCCA”. (A) MZT_1a (Blood), MZT_1a (Germline), MZT_1b (Blood) and MZT_1b (Germline); (B) MZT_2a (Blood) and MZT_2b (Blood) and (C) MZT_3a (blood) and MZT_3b (Blood). [file 1471-2156-15-19-S1.docx]

**Additional file 1**: **3.56 Kb sequence of DYZ1arrayfrom all three twin pairs, in adjusted frame of “TTCCA”.** (A) MZT1a (Blood), MZT1a (Germline), MZT1b (Blood) and MZT1b (Germline); (B) MZT2a (Blood) and MZT2b (Blood) and (C) MZT3a (blood) and MZT3b (Blood).

**(A)**

**>MZT1a (Blood)**

--CCT GTCCA TTACA CTACA TTCCC TTCCA TTCCA ATGAA TTCCA TTCCA TTCCA ATCCA TTCCT TTCCT 68

TTCGC TTGCA TTCCA TTCTA TTCCC TTCTA CTGCA TACAA TTTCA CTCCA TTCGT TCCCA TTCCA TTCAA 138

TTCCA TTCCA TTCAA TTCCA TTCCA TTTGT TTCCA TTCTC TTCGA TTCCA TTTCT TTATA TTCCA TGCCA 208

TTCGA TTCCA TTCTA TTGGA TTGCA TTACA TTCGT GTTCA TTCCA TTCCA GACCA TTCCA TTTGA CTCCA 278

TTCCT TTCGA GCCCT TTCAA TTTGA GTCCA TTCCT TTCCA GTCCA TTTCA CTCCA GTCCA TTACT ATCCA 348

TTCCA TACCA TTCCA TCCCA TTCCA TTCCA TTCCA TTCCA TTCCA TTCCA TTCCA TTCCA TTGCA TTCCA 418

TTCCA TTCCA TTCCA TTGCA CTGCA CTCCA TTCCA TTACA TTCTA CTCTA TCTGA GTCGA TTTTA TTGCA 488

TTAGA TTCTA TTCCA TTGGA TTACT TTCCA TTCGA TTACA TTCCA TTCAT GTACA TTCCA TTCCA GTCAA 558

TTACA TTCGA GTTCA TTACA TTACA TTCCA GTATA TTCCA TTGTA TTCGA TCCCA TTCCT TTCAA TTCCA 628

TTTCA TTCGA CTCCA TTATA TTCGA TTCCA TTCCA CTCGA ATCCA TTCCA TTAGA GGACA TTCCA TTCCA 698

ATGCA TTCCA TTCCA TTCCA TAGCA TTCCA TTGCA TTCGA TTCCA TTCCA TTTGA TGCCA TTCCA TTTGA 768

TGCCA TTCCA TGACA TTCCA TTCCA TTCGA GTCCA TTCCG TTCCA ATTCA TTCCA TTCCG TTTCA TGAAA 838

TTCGA GTCCT TTCCA GTACA TTTCA TTCCA ATCCC ATCCA ATCCC ATCTA CTCCA TTCAA TTCCT TTCCA 908

TTCCA TTTGA TTTGA TTCCA TTGAT TTG-A TTCCA TTCAG TTTGA TTCCA TTCCG TGAAA TTTCG TTCCA 977

TTCTA TTCCA TTGCA TTACT TTCCA TTCAA TTCCA TTCCA TTTCA TTTCA GTCCA TTCGC TTCCT TTCCT 1047

TTCGA TTCAA TTCCA TTTGA TTCCA CTCCA TTCTA TGCGA TTTCA TTCCA ATCGA TTCAA TTCCA TTCGA 1117

TGACA TTCCT TTCGT TTCCA TTCCA TTCGA GTCCA TTTAA TTTGA --GCA TTCGT GTCCA TTCTA TTCGA 1185 GTCCA TTCCA TTACA GTCTA TTCTA TTCCC TTCCA TTCCT GTTGA TTCAA TTTCA TTCCC TTCCA TTCGA 1255

TTCCT TTCCA TTCGA TTCCA TTCCT TTCCA TTCCA TTCCA TTCGT TCCCA TTCCA TGTGA TTTCA TTCCA 1325

TTCCA GTCCA TTATA TTCGA GTCCA CTCCA CTCCA TTCTA TTACA TTCAA TTCCT TTTGA GTCCG TTCCA 1395

TAACA CTCCA TTCAT TTCGA TTCCA TTTCT TGCCA GTTTT ---C- TTCCA TTTTA TTCCA TTCCG TTCGA 1461

TTCCA TTCCA TTCGA TTGCA TTCCA TTCGA ATCCT TTCCA TTCCA TTTCA TTCCA TTCCT TTCTA TTCCA 1531

TTCCA TTTCA TTCGA TTTGA TTCCA TTCTG TTCTA TTCCA TTCAA TTCTT TTTCA TTCCA TTCGA ATCCT 1601

TTCTA TTGCA GTCCA TTCCA TTCGA GTCCA TTCCA ATCCC TTCCA TTCCA TTCCA TTACA GTCCA TTCCA 1671

ATAGA TTCCA TTCCT TTGCC TTCCA TTCGA ATCCA TTCCA TTCTA GTCCA TTCCA TTTGA GTCAA TTCCA 1741

TTCCA TTCCA TTCTA TTCCT TTCCA ATCCA TTCGA TTCCA TTCGA TTCAA TTCCA TTTGA TTCTC TTTCA 1811

TTCTA TTTTA TTCCA TGCCA TTTGA TTGCA TTGCA TTCCA TTCCG TTTGA TTCCA GTCCA TTCAA GAAAG 1881

TTCCA TTCCA GTCCA TTGCT TTCCA GTCCA TTCCA TTCCA CTCTA GTCTA TTCCA CTCCA TTCCT TTCCA 1951

TTCCA TTCCA TACTA TTCCA TTCCA TTCCT TTGCA TTCCG T---- TTCCA ATCTA TTCGA GTCCA TTGCA 2017

TTCCA GTCCA ATCCA TTCGA TTACA TTCCT TTTGA TTCCC TGCCA GTCGA TTGCA TTGCA TACTA GACCA 2087

TTCCA AAGGA GTTCA TTCCA TTCTA TCTCC ACACT TTCCA TTCCA CTCTG TTTGA GTCCA TTCCA TTCCA 2157

GTCCA TTTAA TTCAA GGGCA TTCCA TTCCA TTCCA TTCCA TTCCA TTTCA TATTA TTCCA TTCCA TTCAA 2227

TTCCA TTCCA GATGA TTCCA TTCCA TTCTA TACCA TTGCT CTCTG TTCCA TTCCA TTCCA TCTGT CTCCA 2297

TTCCT TTCGT TTCGA TTCCT TTCCA TTCCA TTCCA TTACA TTTGA TCCTA TTTTA TTAAA TTGCA TTCTA 2367

TTCGA GTGAT TTCCA TTCGA GTCCT TTCCA TTCGA TTCCA TTCCA TTCTA TTCCA TTCCT TTGGA TTCCA 2437

TTCCA TTCCG TTCCG TTCAC ATCAA TTCCT TGCGA TTCCA TTACA TTCGA TTTCT TGCCA TTCGA TTCCA 2507

TTCCT TTTGA CTCCA TTTCA TTCGA TTCCA TTCCA TTCCA TTAAT TTCCA TTCCA TTCGA GACCT TTCCA 2577

TTGCA GTCTT TTCCC TTCGA GTCCA TTCCG TTCGA TTCCC TTCCA TTCGA TTCCA TTCCA TTGGA GTCCG 2647

TACCA GTCGA GTCCA TTCTA TTCCA GTCCA TTAGT TTCGA CTCCA TTGCA TTCGA GTGCA TTCCA TTCCG 2717

TGGCT GTCCA TTCCA TTCCG TTTGA TGCCA TTCCA TACGA TTCCA TTCAA TTCGA GACCA TTCTA TTCCT 2787

GTCCA TTCCT TGTGG TTCGA TTCCA TTTCA CTCTA GTCCA TTCCA TTCCA TTCAA TTCCA TTCGA CTCTA 2857

TTCCG TTCCA TTCAA TTCCA TTCCA TTCGA TTCCA TTTTT TTCGA GAACC TTCCA TTACA CTCCC TTCCA 2927

TTCCA GTGCA TTCCA TTCCA GTCTC TTCAG TTCGA TTCCA TTCCA TTCGT TTCGA TTCCT TTCCA TTCCA 2997

GCCCA TTCCA TTCCA TTCCA TTCCT TTCCT TTCCG TTTCA TTAGA TTCCA TTGCA TTCGA TTCCA TTCAA 3067

TTCAA TTCCG TGCTA TTCAA TTTGA TTCAT TTCCA TTTAA TTCCA TTCCA TTAGA TTCCA TTCCG TACGA 3137

TTCCA TTCCT TTTGA ATCCA TTCCA TTGGA GTCCA TTCAC TTCCA GAACA TTCCA TTCCA GTCGA ATCCA 3207

TTCGA GTACA TTCCA TTAAA GTTCA TTACA TTCTA ATACA TTCCA TTCCA TTGCA TTCCA TTCCA TTCCA 3277

TTCGA TGCCA TTCGA TTCCA TTCCA TGCCA AATCA TTGCA TTCCT TTCCA TTCCG TTCCT ATCAA TTCCA 3347

TTCCA TTCGA TTTAG TTCGA TTCTA TTCAC TTCCA TTCCA TTCGA TTCCA TTCCA TTGGA GTCAA TTCCT 3417

TTCGA CACCC AGCCT TTCCA GTCAA TGATT TTGGA TTCCA TTTTT TTGCA TTCCA TTACA TTCTA TGACA 3487

TTCGA TTCCG TTTCA TTGCA TTCCA TTCCA TACAT TTTTA TTCCA TTCGA GACCG TAGCA TTCCA CTTTA 3557

TTCCA GG--- 3564

**>MZT1a (Germline)**

--CCT GTCCA TTACA CTACA TTCCC TTCCA TTCCA ATGAA TTCCA TTCCATTCCA ATCCA TTCCT TTCCT 68

TTCGC TTGCA TTCCA TTCTA TTCCC TTCTA CTGCA TACAA TTTCA CTCCA TTCGT TCCCA TTCCA TTCAA 138

TTCCA TTCCA TTCAA TTCCA TTCCA TTTGT TTCCA TTCTC TTCGA ATCCA TTTCT TTATA TTCCA TGCCA 208

TTCGA TTCCA TTCTA TTGGA TTGCA TTACA TTCGT GTTCA TTCCA TTCCA GACCA TTCCA TTTGA CTCCA 278

TTCCT TTCGA GCCCT TTCAA TTTGA GTCCA TTCCT TTCCA GTCCA TTTCA CTCCA GTCCA TTACT ATCCA 348

TTCCA TACCA TTCCA TCCCA TTCCA TTCCA TTCCA TTCCA TTCCA TTCCA TTCCA TTCCA TTCAA TTCCA 418

TTCCA TTCCA TTGCA CTGCA CTCCA TTCCA TTACA TTCTA CTCTA TCTGA GTCGG TTTTA TTGCA TTAGA 488

TTCTA TTCCA TTGGA TTACT TTCCA TTCGA TTACC TTCCA TTCAT GTACA TTCCA TTCCA GTCAA TTACA 558

TTCGA GTTCA TTACA TTACA TTCCA GTATA TTCCA TTGTA TTCGA TCCCA TTCCT TTCAA TTCCA TTTCA 628

TTCGA CTCCA TTATA TTCGA TTCCA TTCCA CTCGA ATCCA TTCCA TTAGA GGACA TTCCA TTCCA ATGCA 698

TTCCA TTCCA TTCCA TAGCA TTCCA TTGCA TTCGA TTCCA TTCCA TTTGA TGCCA TTCCA TTTGA TGCCA 768

TTCCA TGACA TTCCA TTCCA TTCGA GTCCA TTCCG TTCCA ATTCA TTCCA TTCCG TTTCA TGAAA TTCGA 838

GTCCT TTCCA GTACA TTTCA TTCCA ATCCC ATCCA ATCCC ATCTA CTCCA TTCAA TTCCT TTCCA TTCCA 908

TTTGA TTTGA TTCCA TTGAT TTG-A TTCCA TTCAG TTTGA TTCCA TTCCG TGAAA TTTCG TTCCA TTCTA 977

TTCCA TTGCA TTACT TTCCA TTCAA TTCCA TTCCA TTTCA TTTCA GTCCA TTCGC TTCCT TTCCT TTCGA 1047

TTCAA TTCCA TTTGA TTCCA CTCCA TTCTA TGCGA TTTCA TTCCA ATCGA TTCAA TTCCA TTCGA TGACA 1117

TTCCT TTCGT TTCCA TTCCA TTCGA GTCCA TTTAA TTTGA --GCA TTCGT GTCCA TTCTA TTCGA GTCCA 1185

TTCCA TTACA GTCTA TTCTA TTCCC TTCCA TTCGT GTTGA TTCAA TTTCA TTCCC TTCCA TTCGA TTCCT 1255

TTCCA TTCGA TTCCA TTCCT TTCCA TTCCA TTCCA TTCGT TCCCA TTCCA TGTGA TTTCA TTCCA TTCCA 1325

GTCCA TTATA TTCGA GTCCA CTCCA CTCCA TTCTA TTACA TTCAA TTCCT TTTGA GTCCG TTCCA TAACA 1395

CTCCA TTCAT TTCGA TTCCA TTTCT TGCCA GTTTT ---C- TTCCA TTTTA TTCCA TTCCG TTCGA TTCCA 1461

TTCCA TTCGA TTGCA TTCCA TTCGA ATCCT TTCCA TTCCA TTTCA TTCCA TTCCT TTCTA TTCCA TTCCA 1531

TTTCA TTCGA TTTGA TTCCA TTCTG TTCTA TTCCA TTCAA TTCTT TTTCA TTCCA TTCGA ATCCT TTCTA 1601

TTGCA GTCCA TTCCA TTCGA GTCCA TTCCA ATCCC TTCCA TTCCA TTCCA TTACA GTCCA TTCCA ATAGA 1671

TTCCA TTCCT TTGCC TTCCA TTCGA ATCCA TTCCA TTCTA GTCCA TTCCA TTTGA GTCAA TTCCA TTCCA 1741

TTCCA TTCTA TTCCT TTCCA ATCCA TTCGA TTCCA TTCGA TTCAA TTCCA TTTGA TTCTC TTTCA TTCTA 1811

TTTTA TTCCA TGCCA TTTGA TTGCA TTGCA TTCCA TTCCG TTTGA TTCCA GTCCA TTCAA GAAAG TTCCA 1881

TTCCA GTCCA TTGCT TTCCA GTCCA TTCCA TTCCA CTCTA GTCTA TTCCA CTCCA TTCCT TTCCA TTCCA 1951

TTCCA TACTA TTCCA TTCCA TTCCT TTGCA TTCCG T---- TTCCA ATCTA TTCGA GTCCA TTGCA TTCCA 2017

GTCCA ATCCA TTCGA TTACA TTCCT TTTGA TTCCC TGCCA GTCGA TTGCA TTGCA TACTA GACCA TTCCA 2087

AAGGA GTCCA TTCCA TTCTA TCTCA ACACT TTCCA TTCCA CTCTG TTCGA GTCCA TTCCA TTCCA GTCCA 2157

TTTAA TTCAA GGGCA TTCCA TTCCA TTCCA TTCCA TTCCA TTTCA TATTA TTCCA TTCCA TTCAA TTCCA 2227

TTCCA GATGA TTCCA TTCCA TTCTA TACCA TTGCT CTCTG TTCCA TTCCA TTCCA TCTGT CTCCA TTCCT 2297

TTCGT TTCGA TTCCT TTCCA TTCCA TTCCA TTACA TTTGA TCCTA TTTTA TTAAA TTGCA TTCTA TTCGA 2367

GTGAT TTCCA TTCGA GTCCT TTCCA TTCGA TTCCA TTCCA TTCTA TTCCA TTCCT TTGGA TTCCA TTCCA 2437

TTCCG TTCCG TTCAC ATCAA TTCCT TGCGA TTCCA TTACA TTCGA TTTCT TGCCA TTCGA TTCCA TTCCT 2507

TTTGA CTCCA TTTCA TTCGA TTCCA TTCCA TTCCA TTAAT TTCCA TTCCA TTCGA GACCT TTCCA TTGCA 2577

GTCTT TTCCC TTCGA GTCCA TTCCG TTCGA TTCCC TTCCA TTCGA TTCCA TTCCA TTGGA GTCCG TACCA 2647

GTCGA GTCCA TTCTA TTCCA GTCCA TTAGT TTCGA CTCCA TTGCA TTCGA GTGCA TTCCA TTCCG TGGCT 2717

GTCCA TTCCA TTCCG TTTGA TGCCA TTCCA TACGA TTCCA TTCAA TTCGA GACCA TTCTA TTCCT GTCCA 2787

TTCCT TGTGG TTCGA TTCCA TTTCA CTCTA GTCCA TTCCA TTCCA TTCAA TTCCA TTCGA CTCTA TTCCG 2857

TTCCA TTCAA TTCCA TTCCA TTCGA TTCCA TTTTT TTCGA GAACC TTCCA TTACA CTCCC TTCCA TTCCA 2927

GTGCA TTCCA TTCCA GTCTC TTCAG TTCGA TTCCA TTCCA TTCGT TTCGA TTCCT TTCCA TTCCA GCCCA 2997

TTCCA TTCCA TTCCA TTCCT TTCCT TTCCG TTTCA TTAGA TTCCA TTGCA TTCCA TTCCA TTCAA TTCAA 3067

TTCCG TGCTA TTCAA TTTGA TTCAT TTCCA TTTAA TTCCA TTCCA TTAGA TTCCA TTCCG TACGA TTCCA 3137

TTCCT TTTGA ATCCA TTCCA TTGGA GTCCA TTCAC TTCCA GAACA TTCCA TTCCA GTCGA ATCCA TTCGA 3207

GTACA TTCCA TTAAA GTTCA TTACA TTCTA ATACA TTCCA TTCCA TTGCA TTCCA TTCCA TTCCA TTCGA 3277

TGCCA TTCGA TTCCA TTCCA TGCCA AATCA TTGCA TTCCT TTCCA TTCCG TTCCT ATCAA TTCCA TTCCA 3347

TTCGA TTTAG TTCGA TTCTA TTCAC TTCCA TTCCA TTCGA TTCCA TTCCA TTGGA GTCAA TTCCT TTCGA 3417

CACCC AGCCT TTCCA GTCAA TGATT TTGGA TTCCA TTTTG TTGCA TTCCA TTACA TTCTA TGACA TTCGA 3487

TTCCG TTTCA TTGCA TTCCA TTCCA TACAT TTTTA TTCCA TTCGA GACCG TAGCA TTCCA CTTTA TTCCA 3557

GG--- 3559

**>MZT1b (Blood)**

--CCT GTCCA TTACA CTACA TTCCC TTCCA TTCCA ATGAA TTCCA TTCCA TTCCA ATCCA TTCCT TTCCT 70

TTCGC TTGCA TTCCA TTCTA TTCTC TTCTA CTGCA TACAA TTTCA CTCCA TTCGT TCCCA TTCCA TTCAA 138

TTCCA TTCCA TTCAA TTCCA TTCCA TTTGT TTCCA TTCTC TTCGA TTCCA TTTCT TTATA TTCCA TGCCA 208

TTCGA TTCCA TTCTA TTGGA TTGCA TTACA TTCGT GTTCA TTCCA TTCCA GACCA TTCCA TTTGA CTCCA 278

TTCCT TTCGA GCCCT TTCAA TTTGA GTCCA TTCCT TTCCA GTCCA TTTCA CTCCA GTCCA TTACT ATCCA 348

TTCCA TACCA TTCCA TCCCA TTCCA TTCCA TTCCA TTCCA TTGCA TTCCA TTCCA TTCCA TTCCA TTGCA 418

CTGCA CTCCA TTCCA TTACA TTCTA CTCTA TCTGA GTCGA TTTTA TTGCA TTAGA TTCTA TTCCA TTGGA 488

TTACT TTCCA TTCGA TTACA TTCCA TTCAT GTACA TTCCA TTCCA GTCAA TTACA TTCGA GTTCA TTACA 558

TTACA TTCCA GTATA TTCCA TTGTA TTCGA TCCCA TTCCT TTCAA TTCCA TTTCA TTCGA CTCCA TTATA 628

TTCGA TTCCA TTCCA CTCGA ATCCA TTCCA TTAGA GGACA TTCCA TTCCA ATGCA TTCCA TTCCA TTCCA 698

TAGCA TTCCA TTGCA TTCGA TTCCA TTCCA TTTGA TGCCA TTCCA TTTGA TGCCA TTCCA TGACA TTCCA 768

TTCCA TTCGA GTCCA TTCCG TTCCA ATTCA TTCCA TTCCG TTTCA TGAAA TTCGA GTCCT TTCCA GTACA 838

TTTCA TTCCA ATCCC ATCCA ATCCC ATCTA CTCCA TTCAA TTCCT TTCCA TTCCA TTTGA TTTGA TTCCA 908

TTGAT TT-GA TTCCA TTCAG TTTGA TTCCA TTCCG TGAAA TTTCG TTCCA TTCTA TTCCA TTGCA TTACT 977

TTCCA TTCAA TTCCA TTCCA TTTCA TTTCA GTCCA TTCGC TTCCT TTCCT TTCGA TTCAA TTCCA TTTGA 1047

TTCCA CTCCA TTCTA TGCGA TTTCA TTCCA ATCGA TTCAA TTCCA TTCGA TGACA TTCCT TTCGT TTCCA 1117

TTCCA TTCGA GTCCA TTTAA TTTGA –-GCA TTCGT GTCCA TTCTA TTCGA GTCCA TTCCA TTACA GTCTA 1185

TTCTA TTCCC TTCCA TTCGT GTTGA TTCAA TTTCA TTCCC TTCCA TTCGA TTCCT TTCCA TTCGA TTCCA 1255

TTCCT TTCCA TTCCA TTCCA TTCGT TCCCA TTCCA TGTGA TTTCA TTCCA TTCCA GTCCA TTATA TTCGA 1325

GTCCA CTCCA CTCCA TTCTA TTACA TTCAA TTCCT TTTGA GTCCG TTCCA TAACA CTCCA TTCAT TTCGA 1395

TTCCA TTTCT TGCCA GTTTT ---C- TTCCA TTTTA TTCCA TTCCG TTCGA TTCCA TTCCA TTCGA TTGCA 1461

TTCCA TTCGA ATCCT TTCCA TTCCA TTTCA TTCCA TTCCT TTCTA TTCCA TTCCA TTTCA TTCGA TTTGA 1531

TTCCA TTCTG TTCTA TTCCA TTCAA TTCTT TTTCA TTCCA TTCGA ATCCT TTCTA TTGCA GTCCA TTCCA 1601

TTCGA GTCCA TTCCA ATCCC TTCCA TTCCA TTCCA TTACA GTCCA TTCCA ATAGA TTCCA TTCCT TTGCC 1671

TTCCA TTCGA ATCCA TTCCA TTCTA GTCCA TTCCA TTCCA TTCCA TTCTA TTCCT TTCCA ATCCA TTCGA 1741

TTCCA TTCGA TTCAA TTCCA TTTGA TTCTC TTTCA TTCTA TTTTA TTCCA TGCCA TTTGA TTGCA TTGCA 1811

TTCCA TTCCG TTTGA TTCCA GTCCA TTCAA GAAAG TTCCA TTCCA GTCCA TTGCT TTCCA GTCCA TTCCA 1881

TTCCA CTCTA GTCTA TTCCA CTCCA TTCCT TTCCA TTCCA TTCCA TACTA TTCCA TTCCA TTCCT TTGCA 1951

TTCCG TTTGC AATCT ATTCG AGTCC ATTTC ----A TTCCA GTCCA ATCCA TTCGA TTACA TTCCT TTTGA 2017

TTCCC TGCCT GTCGA TTGCA TTGCA TACTA GACCA TTCCA AACGA GTCCA TTCCA TTCTA TCTCA ACACT 2087

TTCCA TTCCA CTCTG TTCGA GTCCA TTCCA TTCCA GTCCA TTTAA TTCAA GGGCA TTCCA TTCCA TTCCA 2157

TTCCA TTCCA TTTCA TATTA TTCCA TTCCA TTCAA TTCCA TTCCA GATGA TTCCT TTCCA TTCTA TACCA 2227

TTGCT CTCTG TTCTA TTCCA TTCCA TCTGT CTCCA TTCCT TTCGT TTCGA TTCCT TTCCA TTCCA TTCCA 2297

TTACA TTTGA TCCTA TTTTA TTAAG TTGCA TTCTA TTCGA GTGAT TTCCA TTCGA GTCCT TTCCA TTCGA 2367

TTCCA TTCCA TTCTA TTCCA TTCCT TTGGA TTCCA TTCCA TTCCG TTCCG TTCAC ATCAA TTCCT TGCGA 2437

TTCCA TTACA TTCGA TTTCT TGCCA TTCGA TTCCA TTCCT TTTGA CTCCA TTTCA TTCGA TTCCA TTCCA 2507

TTCCA TTAAT TTCCA TTCCA TTCGA GACCT TTCCA TTGCA GTCTT TTCCC TTCGA GTCCA TTCCG TTCGA 2577

TTCCC TTCCA TTCGA TTCCC TTCCA TTGGA GTCCG TACCA GTCGA GTCCA TTCTA TTCCA GTCCA TTAGT 2647

TTCGA CTCCA TTGCA TTCGA GTGCA TTCCA TTCCG TGGCT GTCCA TTCCA TTCCG TTTGA TGCCA TTCCA 2717

TACGA TTCCA TTCAA TTCGA GACCA TTCTA TTCCT GTCCA TTCCT TGTGG TTCGA TTCCA TTTCA CTCTA 2787

GTCCA TTCCA TTCCA TTCAA TTCCA TTCGA CTCTA TTCCG TTCCA TTCAA TTCCA TTCCA TTCGA TTCCA 2857

TTTTT TTCGA GAACC TTCCA TTACA CTCCC TTCCA TTCCA GTGCA TTCCA TTCCA GTCTC TTCAG TTCGA 2927

TTCCA TTCCA TTCGT TTCGA TTCCT TTCCA TTCCA GCCCA TTCCA TTCCA TTCCA TTCCT TTCCT TTCCG 2997

TTTCA TTAGA TTCCA TTGCA TTCGA TTCCA TTCAA TTCAA TTCCG TGCTA TTCAA TTTGA TTCAT TTCCA 3067

TTTAA TTCCA TTCCA TTAGA TTCCA TTCCG TACGA TTCCA TTCCT TTTGA ATCCA TTCCA TTGGA GTCCA 3137

TTCAC TTCCA GAACA TTCCA TTCCA GTCGA ATCCA TTCGA GTACA TTCCA TTAAA GTTCA TTACA TTCTA 3207

ATACA TTCCA TTCCA TTGCA TTCCA TTCCA TTCCA TTCGA TGCCA TTCGA TTCCA TTCCA TGCCA AATCA 3277

TTGCA TTCCT TTCCA TTCCG TTCCT ATCAA TTCCA TTCCA TTCGA TTTAG TTCGA TTCTA TTCAC TTCCA 3347

TTCCA TTCGA TTCCA TTCCA TTGGA GTCAA TTCCT TTCGA CACCC AGCCT TTCCA GTCAA TGATT TTGGA 3417

TTCCA TTTTT TTGCA TTCCA TTACA TTCTA TGACA TTCGA TTCCG TTTCA TTGCA TTCCA TTCCA TACAT 3487

TTTTA TTCCA TTCGA GACCG TAGCA TTCCA CTTTA TTCCA GG--- 3529

**>MZT1b (Germline)**

--CCT GTCCA TTACA CTACA TTCCC TTCCA TTCCA ATGAA TTCCA TTCCA TTCCA ATCCA TTCCT TTCCT 68

TTCGC TTGCA TTCCA TTCTA TTCTC TTCTA CTGCA TACAA TTTCA CTCCA TTCGT TCCCA TTCCA TTCAA 138 TTCCA TTCCA TTCAA TTCCA TTCCA TTTGT TTCCA TTCTC TTCGA TTCCA TTTCT TTATA TTCCA TGCCA 208

TTCGA TTCCA TTCTA TTGGG TTGCA TTACA TTCGT GTTCA TTCCA TTCCA GACCA TTCCA TTTGA CTCCA 278

TTCCT TTCGA GCCCT TTCAA TTTGA GTCCA TTCCT TTCCA GTCCA TTTCA CTCCA GTCCA TTACT ATCCA 348

TTCCA TACCA TTCCA TCCCA TTCCA TTCCA TTCCA TTCCA TTCCA TTCCA TTCCA TTGCA TTCCA TTCCA 418

TTCCA TTCCA TTGCA CTGCA CTCCA TTCCA TTACA TTCTA CTCTA TCTGA GTCGA TTTTA TTGCA TTAGA 488

TTCTA TTCCA TTGGA TTACT TTCCA TTCGA TTACA TTCCA TTCAT GTACA TTCCA TTCCA GTCAA TTACA 558

TTCGA GTTCA TTACA TTACA TTCCA GTATA TTCCA TTGTA TTCGA TCCCA TTCCT TTCAA TTCCA TTTCA 628

TTCGA CTCCA TTATA TTCGA TTCCA TTCCA CTCGA ATCCA TTCCA TTAGA GGACA TTCCA TTCCA ATGCA 698

TTCCA TTCCA TTCCA TAGCA TTCCA TTGCA TTCGA TTCCA TTCCA TTTGA TGCCA TTCCA TTTGA TGCCA 768

TTCCA TGACA TTCCA TTCCA TTCGA GTCCA TTCCG TTCCA ATTCA TTCCA TTCCG TTTCA TGAAA TTCGA 838

GTCC- TTCCA GTACA TTTCA TTCCA ATCCC ATCCA ATCCC ATCTA CTCCA TTCAA TTCCT TTCCA TTCCA 907

TTTGA TTTGA TTCCA TTGAT TTG-A TTCCA TTCAG TTTGA TTCCA TTCCG TGAAA TTTCG TTCCA TTCTA 976

TTCCA TTGCA TTACT TTCCA TTCAA TTCCA TTCCA TTTCA TTTCA GTCCA TTCGC TTCCT TTCCT TTCGA 1046

TTCAA TTCCA TTTGA TTCCA CTCCA TTCTA TGCGA TTTCA TTCCA ATCGA TTCAA TTCCA TTCGA TGACA 1116

TTCCT TTCGT TTCCA TTCCA TTCGA GTCCA TTTAA TTTGA –-GCA TTCGT GTCCA TTCTA TTCGA GTCCA 1184

TTCCA TTACA GTCTA TTCTA TTCCC TTCCA TTCGT GTTGA TTCAA TTTCA TTCCC TTCCA TTCGA TTCCT 1254

TTCCA TTCGA TTCCA TTCCT TTCCA TTCCA TTCCA TTCGT TCCCA TTCCA TGTGA TTTCA TTCCA TTCCA 1324

GTCCA TTATA TTCGA GTCCA CTCCA CTCCA TTCTA TTACA TTCAA TTCCT TTTGA GTCCG TTCCA TAACA 1394

CTCCA TTCAT TTCGA TTCCA TTTCT TGCCA GTTTT ---C- TTCCA TTTTA TTCCA TTCCG TTCGA TTCCA 1460

TTCCA TTCGA TTGCA TTCCA TTCGA ATCCT TTCCA TTCCA TTTCA TTCCA TTCCT TTCTA TTCCA TTCCA 1530

TTTCA TTCGA TTTGA TTCCA TTCTG TTCTA TTCCA TTCAA TTCTT TTTCA TTCCA TTCGA ATCCT TTCTA 1600

TTGCA GTCCA TTCCA TTCGA GTCCA TTCCA ATCCC TTCCA TTCCA TTCCA TTACA GTCCA TTCCA ATAGA 1670

TTCCA TTCCT TTGCC TTCCA TTCGA ATCCA TTCCA TTCTA GTCCA TTCCA TTTGA GTCAA TTCCA TTCCA 1740

TTCCA TTCTA TTCCT TTCCA ATCCA TTCGA TTCCA TTCGA TTCAA TTCCA TTTGA TTCTC TTTCA TTCTA 1810

TTTTA TTCCA TGCCA TTTGA TTGCA TTGCA TTCCA TTCCG TTTGA TTCCA GTCCA TTCAA GAAAG TTCCA 1880

TTCCA GTCCA TTGCT TTCCA GTCCA TTCCA TTCCA CTCTA GTCTA TTCCA CTCCA TTCCT TTCCA TTCCA 1950

TTCCA TACTA TTCCA TTCCA TTCCT TTGCA TTCCG T---- TTCCA ATCTA TTCGA GTCCA TTGCA TTCCA 2016

GTCCA ATCCA TTCGA TTACA TTCCT TTTGA TTCCC TGCCA GTCGA TTGCA TTGCA TACTA CACCA TTCCA 2086

AAGGA GTCCA TTCCA TTCTA TCTCA ACACT TTCCA TTCCA CTCTG TTCGA GTCCA TTCCA TTCCA GTCCA 2156

TTTAA TTCAA GGGCA TTCCA TTCCA TTCCA TTCCA TTCCA TTTCA TATTA TTCCA TTCCA TTCAA TTCCA 2226

TTCCA GATGA TTCCA TTCCA TTCTA TACCA TTGCT CTCTG TTCCA TTCCA TTCCA TCTGT CTCCA TTCCT 2296

TTCGT TTCGA TTCCT TTCCA TTCCA TTCCA TTACA TTTGA TCCTA TTTTA TTAAA TTGCA TTCTA TTCGA 2366

GTGAT TTCCA TTCGA GTCCT TTCCA TTCGA TTCCA TTCCA TTCTA TTCCA TTCCT TTGGA TTCCA TTCCA 2436

TTCCG TTCCG TTCAC ATCAA TTCCT TGCGA TTCCA TTACA TTCGA TTTCT TGCCA TTCAA TTCCA TTCCT 2506

TTTGA CTCCA TTTCA TTCGA TTCCA TTCCA TTCCA TTAAT TTCCA TTCCA TTCGA GACCT TTCCA TTGCA 2576

GTCTT TTCCC TTCGA GTCCA TTCCG TTCGA TTCCC TTCCA TTCGA TTCCA TTCCA TTGGA GTCCG TACCA 2646

GTCGA GTCCA TTCTA GTCCA TTAGT TTCGA CTCCA TTGCA TTCGA GTGCA TTCCA TTCCA TTCCG TGGCT 2716

GTCCA TTCCA TTCCG TTTGA TGCCA TTCCA TACGA TTCCA TTCAA TTCGA GACCA TTCTA TTCCT GTCCA 2786

TTCCT TGTGG TTCGA TTCCA TTTCA CTCTA GTCCA TTCCA TTCCA TTCAA TTCCA TTCGA CTCTA TTCCG 2856

TTCCA TTCAA TTCCA TTCCA TTCGA TTCCA TTTTT TTCGA GAACC TTCCA TTACA CTCCC TTCCA TTCCA 2926

GTGCA TTCCA TTCCA GTCTC TTCAG TTCGA TTCCA TTCCA TTCGT TTCGA TTCCT TTCCA TTCCA GCCCA 2996

TTCCA TTCCA TTCCA TTCCT TTCCT TTCCG TTTCA TTAGA TTCCA TTGCA TTCGA TTCCA TTCAA TTCAA 3066

TTCCG TGCTA TTCAA TTTGA TTCAT TTCCA TTTAA TTCCA TTCCA TTAGA TTCCA TTCCG TACGA TTCCA 3136

TTCCT TTTGA ATCCA TTCCA TTGGA GTCCA TTCAC TTCCA GAACA TTCCA TTCCA GTCGA ATCCA TTCGA 3206

GTACA TTCCA TTAAT GTTCA TTACA TTCTA ATACA TTCCA TTCCA TTGCA TTCCA TTCCA TTCCA TTCGA 3276

TGCCA TTCGA TTCCA TTCCA TGCCA AATCA TTGCA TTCCT TTCCA TTCCG TTCCT ATCAA TTCCA TTCCA 3346

TTCGA TTTAG TTCGA TTCTA TTCAC TTCCA TTCCA TTCGA TTCCA TACCA TTGGA GTCAA TTCCT TTCGA 3416

CGCCC AGCCT TTCCA GTCAA TGATT TTGGA TTCCA TTTTT TTGCA TTCCA TTACA TTCTA TGACA TTCGA 3486

TCCGT TTCAT T-GCA TTCCA TTCCA TACAT TTT-A TTCCA TTCGA GACCG TAGCA T-CCA CTTTA TTCCA 3553

GG--- 3555

**(B)**

**>MZT2a (Blood)**

--CCT GTCCA TTACA CTACA TTCCC TTCCA TTCCC ATGAA TTCCA TTCCA TTCCA ATCCA TTCCT TTCCT 68

TTCGC TTGCA TTCCA CTCTT TTCTC TTCTA CTGCA TACAA TTTCA CTACA TTCGT TCCCA TTCCA TTCAA 138

TTCCA TTCCA TTCAA TTCCA TTCCG TTTGT TTCCA TTCTC TTCGA TTCCA TTTCT TTATA TTCCA TGCCA 208

TTCGA TTCCA TTCTA TTGGA TTGCA TTACA TTCGT GTTCA TTCCA TTCCA GACCA TTCCA TTTGA CTCCA 278

TTCCT TTCGA GCCCT TTCAA TTTGA GTCCA TTCCT TTCCA GTCCA TTTCC CTCCA GTCCA TTACT ATCCA 348

TTCCA TACCA TTCCA TCCCA TTCCA TTCCA TTCCA TTCCA TTCCA TTGCA TTCCA TTCCA TTCCA TTCCA 418

TTGCA CTGCA CTCCA TTCCA TTACA TTCTA CTCTA TTTGA GTCGA TTTTA TTGCA TTAGA TTCTA TTCCA 488

TTGGA TTGCT TTCCA TTCGA TTACA TTCCA TTCAT GTACA TTCCA TTCCA GTCAA TTACA TTCGA GTTCA 558

TTACA TTACA TTCCA GTATA TTCCA TTGTA TTCGG TCCCA TTCCT TTCAA TTCCC TTTGA TTAGA CTCCA 628

TTATA TTCGA TTCCA TTCCA CTCGA ATACA TTCCA TTAGA GGACA TTCCA TTCCA ATGCA TTCCA TTCCA 698

TTCCA TAGCA TTCCA TCGCA TTCGA TTCCA TTCCA TTTGA TGCCA TTCCA TGACA TTCCA TTCCA TTCGA 768

GTCCA TTCCG TTCTA ATTCA TTCCT TTCCG TTTCA TGAAA TTCGA GTCCT TTCCA GTACA TTTCA TTCCA 838

ATCCC ATCCA ATCCC ATCTA CTCCA TTCAA TTCCT TTCCA TTCCA TTTGA TTTGA TTCCA TTGAT TTG-A 907

TTCCA TTCAG TTTGA TTCCG TTCCG TGAAA TTTCG TTCCA TTCTA TTCCA TTGCA TTACT TTCCA TTCAA 977

TTCCA TTCCA TTTCA TTTCA GTCCA TTCGC TTCCT TTCCT TTCGA TTCAA TTCCA TTTGA TTCCA CTCCA 1047

TTCTA TGCGA TTTCA TTCCA ATCGA TTCAA TTCCA TTCGA TAACA TTCCT TTCGT TTCCA TTCAA TTCGA 1117

GTCCA TTTAA TTTGA –-GCA TTCGT GTCCA TTCTA TCCGA GTCCA TTCCA TTACC GTCTA TTCTA TTCCC 1185

TTCCA TTCCT GTTGA TTCAA TTTCA TTCCC TTCCA TTCGA TTCCT TTCCA TTGGA TTCCA TTCCT TTCCA 1255

TTCCA TTCCA TTCGT TCCCA TTCCA TGTGA TTTCA TTCCA TTCCA GTCCA TTATA TTCGA GTCCA CTCCA 1325

CTCAA TTCTA TTACA TTCAA TTCCT TTTGA GTCCG TTCCA TAACA CTCCA TTCAT TTCGA TTCCA TTTCT 1395

TGCCA GTTTT --C-- TTCCA TTTTA TTCCA TTCCG TTCGA TTCCA TTCCA TTCGA TTGCA TTCCA TTCGA 1461

ATCCT TTCCA TTCCA TTTCA TTCCA TTCCT TTCTA TTCCA TTCCA TTTCA TTCGA TTTGA TTCCA TTCTG 1531

TTCTA TTCCA TTCAA TTCTT TTTCA TTCCA TTCGA ATCCT TTCTA TTGCA GTCCA TTCCA TTCGA GTCCA 1601

TTCCA ATCCC TTCCA TTCCA TTCCA TTACA GTCCA TTCCA ATAGA TTCCA TTCCT TTGCC TTCCA TTCGA 1671

ATCCA TTCCA TTCTA GTCCA TTCCA TTTGA GTCAA TTCCA TTCCA TTCCA TTCTA TTCCT TTCCA ATCCA 1741

TTCGA TTCCA TTCGA TTCAA TTCCA TTTGA TTCTC TTTCA TTCTA TTTTA TTCCA TGCCA TTTGA TTGCA 1811

TTGCA TTCCA TTCCG TTTGA TTCCA GTCCA TTCAA GAAAG TTCCA TTCCA GTCCA TTGCT TTCCA GTCCA 1881

TTCCA TTCCA CTCTA GTCTA TTCCA CTCCA TTCCT TTCCA TTCCA TTCCA TACTA TTCCA TTCCA TTCCT 1951

TTGCA TTCCG T---- TTGCA ATCTA TTCGA GTCCA TTGCA TTCCA GTCCA ATCCA TTCGA TTACA TTCCT 2017

TTTGA TTCCC TGCCA GTCGA TTGCA TTGCA TACTA GACCA TTCCA AAGGA GTCCA TTCCA TTATA TTTCA 2087

ACACT TTCCA TTCCA CTCTG TTCGA GTCCA TTCCA TTCCA GTCCA TTTAA TTCAA GGGCA TTCCA TTCCA 2157

TTCCA TTCCA TTTCA TGTTA TTCCA TTCCA TTCAA TTCCA TTCCA GATGA TTCCA TTCCA TTCTA TACCA 2227

TTGCT CTCTG TTCCA TTCCA TTCCA TCTGT CTCCA TTCCT TTCGT TTCGA TTCCT TTCCA TTCCA TTCCA 2297

TTACA TTTGA TCCTA TTTTA TTAAA TTGCA TTCTA TTCGA GTGAT TTCCA TTCGA GTCCT TTCCA TTCGA 2367

TTCCA TTCCA TTCTA TTCCA TTCCT TTGGA TTCCA TTCCA TTCCG TTCCG TTCAC ATCAA TTCCT TGCGA 2437

TTCCA TTACA TTTGA TTTCT TGCCA TTCGA TTCCA TTCCT TTTGA CTCCA TTTCA TTCGA TTCCA ATCCA 2507

TTCCA TTAAT TTCCA TTCCA TTCGA GACCT TTCCA TTGCA GTCTT TTCCC TTCGA GTCCA TTCCG TTCGA 2577

TTCCC TTGCA TTCGA TTCCA TTCCA TTGGA GTCCG TACCA GTCGA GTCCA TTCTA TTCCA GTCCA TTAGT 2647

TTCGA CTCCA TTGCA TTCGA GTGCA TTCCA TTCCG TGGCT GTCCA TTCCA TTCCG TTTGA TGCCA TTCCA 2717

TACGA TTCCA TTCAA TTCGA GACCA TTCTA TTCCT GTCCA TTCCT TGTGG TTCGA TTCCA TTTCA CTCTA 2787

GTCCA TTCCA TTCCA TTCAA TTCCA TTCGA CTCTA TTCCG TTCCA TTCAA TTCCA TTCCA TTCGA TTCCA 2857

TTTTT TTCGA GAACC TTCCA TTACA CTCCC TTCCA TTCCA GTGCA TTCCA TTCCA GTCTC TTCAG TTCGA 2927

TTCCA TTCCA TTCGT TTCGA TTCCT TTCCA TTCCA GCCCA TTCCA TTCCA TTCCA TTCCT TTCCT TTCCG 2997

TTTCA TTAGA TTCCA TTGCA TTCCA TTCCA TTCAA TTCAA TTCCG TGCTA TTCAA TTTGA TTCAT TTCCA 3067

TTTAA TTCCA TTCCA TTAGA TTCCA TTCCG TACGA TTCCA TTCCT TTTGA ATCCA TTCCA TTGGA GTCCA 3137

TTCAC TTCCA GAACA TTCCA TTCCA GTCGA ATCCA TTCGA GTACA TTCCA TTAAA GTTCA TTACA TTCTA 3207

ATACA TTCCA TTCCA TTGCA TTCCA TTCCA TTCCA TTCGA TGCCA TTCGA TTCCA TTCCA TGCCA AATCA 3277

TTGCA TTCCT TTCCA TTCCG TTCCT ATCAA TTCCA TTCCA TTCGA TTTAG TTCGA TTCTA TTCAC TTCCA 3347

TTCCA TTCGA TTCCA TTCCA TTGGA GTCAA TTCCT TTCGA CACCC AGCCT TTCCA GTCAA TGATT TTGGA 3417

TTCCA TTTTT TTGCA TTCCA TTACA TTCTA TGACA TTCGA TTCCG TTTCA TTGCA TTCCA TTCCA TACAT 3487

TTTTA TTCCA TTCGA GACCG TAGCA TTCCA CTTTA TTCCA GG--- 3529

**>MZT2b (Blood)**

--CCT GTCCA TTACA CTACA TTCCC TTCCA TTCCA ATGAA TTCCA TTCCA TTCCA ATCCA TTCCT TTCCT 68

TTCGC TTGCA TTCCA TTCTA TTCTC TTCTA CTGCA TACAA TTTCC CTCCA TTCGT TCCCA TTCCA TTCAA 138

TTCCA TTCCA TTCAA TTCCA TTCCA TTTGT TTCCA TTCTC TTCGA TTCCA TTTCT TTATA TTCCA TGCCA 208

TTCGA TTCCA TTCTA TTGGG TTGCA TTACA TTCGT GTTCA TTCCA TTCCA GACCA TTCCA TTTGA CTCCA 278

TTCCT TTCGA GCCCT TTCAA TTTGA GTCCA TTCCT TTCCA GTCCA TTTCA CTCCA GTCCA TTACT ATCCA 348

TTCCA TACCA TTCCA TCCCA TTCCA TTCCA TTCCA TTCCA TTCCA TTCCA TTCCA TTCCA TTGCA TTCCA 418

TTCCA TTCCC ----A TTCCA TTGCA CTGCA CTCCA TTCCA TTACA TTCTA CTCTA TCTGA GTCGA TTTTA 484

TTGCA TTAGA TTCTA TTCCA TTGGA TTACT TTCCA TTCGA TTACA TTCCA TTCAT GTACA TTCCA TTCCA 554

GTCAA TTACA TTCGA GTTCA TTACA TTACA TTCCA GTATA TTCCA TTGTA TTCGA TCCCA CTCCT TTCAA 624

TTCCA TTTCT TTCGA CTCCA TTATA TTCGA TTCCA TTCCA CTCGA ATCCA TTCCA TTAGA GGACA TTCCA 694

TTCCA ATGCA TTCCA TTCCA TTCCA TAGCA TTCCA TTGCA TTCGA TTCCA TTCCA TTTGA TGCCA TTCCA 764

TTTGA TGCCA TTCCA TGACA TTCCA TTCCA TTCGA GTCCA TTCCG TTCCA ATTCA TTCCA TTCCG TTTCA 834

TGAAA TTCGA GTCCT T-CCA GTACA TTTCA TTCCA ATCCC ATCCA ATCCC ATCTA CTCCA TTCAA TTCCT 903

TTCCA TTCCA TTTGA TTTGA TTCCA TTGAT TTG-A TTCCA TTCAG TTTGA TTCCA TTCCG TGAAA TTTCG 972

TTCCA TTCTA TTCCA TTGCA TTACT TTCCA TTCAA TTCCA TTCCA TTTCA TTTCA GTCCA TTCGC TTCCT 1042

TTCCT TTCGA TTCAA TTCCA TTTGA TTCCA CTCCA TTCTA TGCGA TTTCA TTCCA ATCGA TTCAA TTCCA 1112

TTCGA TGACA TTCCT TTCGT TTCCA TTCCA TTCGA GTCCA TTTAA TTTGA –-GCA TTCGT GTCCA TTCTA 1180

TTCGA GTCCA TTCCA TTACA GTCTA TTCTA TTCCC TTCCA TTCCT GTTGA TTCAA TTTCA TTCCC TTCCA 1250

TTCGA TTCCT TTCCA TTCGA TTCCA TTCCT TTCCA TTCCA TTCCA TTCGT TCCCA TTCCA TGTGA TTTCA 1320

TTCCA TTCCA GTCCA TTATA TTCGA GTCCA CTCCA CTCCA TTCTA TTACA TTCAA TTCCT TTTGA TTCCG 1390

TTCCA TAACA CTCCA TTCAT TTCGA TTCCA TTTCT TGCCA GTTTT –--C- TTCCA TTTTA TTCCA TTCCG 1456

TTCGA TTCCA TTCCA TTCGA TTGCA TTCCA TTCGA ATCCT TTCCA TTCCA TTTCA TTCCA TTCCT TTCTA 1526

TTCCA TTCCA TTTCA TTCGA TTTGA TTCCA TTCTG TTCTA TTCCA TTCAA TTCTT TTTCA TTCCA TTCGA 1596

ATCCT TTCTA TTGCA GTCCA TTCCA TTCGA GTCCA TTCCA ATCCC TTCCA TTCCA TTCCA TTACA GTCCA 1666

TTCCA ATAGA TTCCA TTCCT TTGCC TTCCA TTCGA ATCCA TTCCA TTCTA GTCCA TTCCA TTTGA GTCAA 1736

TTCCA TTCCA TTCCA TTCTA TTCCT TTCCA ATCCA TTCGA TTCCA TTCGA TTCAA TTCCA TTTGA TTCTC 1806

TTTCA TTCTA TTTTA TTCCA TGACA TTTGA TTGCA TTGCA TTCCA TTCCG TTTGA TTCCA GTCCA TTCAA 1876

GAAAC TTCCA TTCCA GTCCA TTGCT TTCGA GTCCA TTCCA TTCCA CTCTA GTCTA TTCCA CTCCA TTCCT 1946

TTGCA TTCCA TTCCA TACTA TTCCA TTCCA TTCCT TTGCT TTCCG T---- TTCCA ATCTA TTCGA GTCCA 2012

TTGCA TTCCA GTCCA ATCCA TTCGA TTACA TTCCT TTTGA TTCCC TGCCA GTTGA TTGCA TTGCA TACTA 2082

GACCA TTCCA AACGA GTCCA TTCCA TTCTA TTTCA ACACT TTCCA TTCCA CTCTG TTCGA GTCCA TTGCA 2152

TTCCA GTCCA TTTAA TTCAA GGGCA TTCCA TTCCA TTCCA TTCCA TTTCA TATTA TTCCA TTCCA TTCAA 2222

TTCCA TTCCA GATGA TTCCA TTCCA TTCTA TACCA TTGCT CTCTG TTCCA TTCCA TTCCA TCTGT CTCCA 2292

TTCCT TTCGT TTCGA TTCCT TTCCA TTCCA TTCCA TTACA TTTCA TCGTA TTTTA TTCAA TTGCA TTCTA 2362

TTCGA GTGAT TTCCA TTCGA GTTCT TTCCA TTCGA TTCCA TTCCA TTCTA TTCCA TTTCT TTGGA TTCCA 2432

TTCCA TTCCG TTCCG TTCAC ATCAA TTCCT TGCGA TTCCA TTACA TTCGA TTTCT TGCCA TTCGA TTCCA 2502

TTCCT TTTGA CTCCA TTTCA TTCGA TTCCA TTCCA TTCCA TTAAT TTCCA TTCCA TTCGA GACCT TTCCA 2572

TTGCA GTCTT TTCCC TTCGA GTCCA TTCCG TTCGA TTCCC TTCCA TTCGA TTCCA TTCCA TTGGA GTCCG 2642

TACCA GTCGA GTCCA TTCTA TTCCA GTCCA TTAGT TTCGA CTCCA TTGCA TTCGA GTGCA TTCCA TTCCG 2712

TGGCT GTCCA TTCCA TTCCG TTTGA TGCCA TTCCA TACGA TTCCA TTCAA TTCGA GACCA TTCTA TTCCT 2782

GTCCA TTCCT TGTGG TTCGA TTCCA TTTCA CTCTA GTCCA TTCCA TTCCA TTCAA TTCCA TTCGA CTCTA 2852

TTCCG TTCCA TTCAA TTGCA TTCCA TTCGA TTCCA TTTTT TTCGA GAACC TTCCA TTACA CTCCC TTCCA 2922

TTCCA GTGCA TTCCA TTCCA GTCTC TTCAG TTCGA TTCCA TTCCA TTCGT TTCGA TTCCT TTCCA TTCCA 2992

GTCCA TTCCA TTCCA TTCCA TTCCT TTCCT TTCCG TTTCA TTAGA TTCCA TTGCA TTCGA TTCCA TTCAA 3062

TTCAA TTCCG TGCTA TTCAA TTTGA TTCAT TTCCA TTTAA TTCCA TTCCA TTAGA TTCCA TTCCG TACGA 3132

TTCCA TTCCT TTTGA ATCCA TTCCA TTGGA GTCCA TTCAC TTCCA GAACA TTCCA TTCCA GTCGA ATCCA 3202

TTCGA GTACA TTCCA TTAAA GTTCA TTACA TTCTA ATACA TTCCA TTCCA TTGCA TTCCA TTCCA TTCCA 3272

TTCGA TGCCA TTCGA TTCCA TTCCA TGCCA AATCA TTGCA ATCCT TTCCA TTCCG TTCCT ATCAA TTCCA 3342

TTCCA TTCGA TTTAG TTCGA TTCTA TTCAC TTCCA TTCCA TTCGA TTCCA TTCCA TTGGA GTCAA TTCCT 3412

TTCGA CACCC AGCCT TTCCA TTCAA TGATT TTGGA TTCCA TTTTT TTGCA TTCCA TTACA TTCTA TGACA 3482

TTCGA TTCCG TTTCA TTGCA TTCCA TTCCA TACAT TTTTA TTCCA TTCGA GACCG TAGCA TTCCA CTTTA 3552

TTCCA GG--- 3559

**(C)**

**>MZT3a (Blood)**

--CCT GTCCA TTACA CTACA TTCCC TTCCA TTCCA ATGAA TTCCA TTCCA TTCCA ATCCA TTCCT TTCCT 68

TTCGC TTGCT TTACA TTCTA TTCCC TTCTA CTGCA TACAA TTTCA CTCCA TTCGT TCCCA TTCCA TTCAA 138

TTCCA TTCCA TTCAA TTCCA TTCCA TTTGT TTCCA TTCTC TTCGA TTCCA TTTCT TTATA TTCCA TGCCA 208

TTCGA TTCCA TTCTA TTGGA TTGCA TTACA TTCGT GTTCA TTCCA ATCCC ATCCA ATCCC ATCTA CTCCA 278

TTCAA TTCCT TTCCA TTCCA TTTGA TTTGA TTCCA TTGAT TTG-A TTCCA TTCAG TTTGA TTCCA TTCCG 347

TGAAA TTTCG TTCCA TTCTA TTCCA TTGCA TTACT TTCCA TTCAA TTCCA TTCCA TTTCA TTTCA GTCCA 417

TTCGC TTCCT TTCCT TTCGA TTCAA TTCCA TTTGA TTCCA CTCCA TTCTA TGCGA TTTCA TTCCA ATCGA 487

TTCAA TTCCA TTCGG TGACA TTCCT TTCGT TTCCA TTCCA TTCGA GTCCA TTTAA TTTGA --GCA TGCGT 555

GTCCA TTCTA TTCGA GTCCA TTCCA TTACC GTCTA TTCTA TTCCC TTCCA TTCCT GTTGA TTCAA TTTCA 625

TTCCC TTCCA TTCGA TTCCT TTCCA TTCGA TTCCA TTCCT TTCCA TTCCA TTCCA TTCGT TCCCA TTCCA 695

TGTGA TTTCA TTCCA TTCCA GTCCA TTATA TTCGA GTCCA CTCCA CTCCA TTCTA TTACA TTCAA TTCCT 765

TTTGA GTCCG TTCCA TAACA CTCCA TTCAT TTCGA TTCCA TTTCT TGCCA GTTTT --C-- TTCCA TTTTA 831

TTCCA TTCCG TTCGA TTCCA TTCCA TTCGA TTGCA TTCCA TTCGA ATCCT TTCCA TTCCA TTTCA TTCCA 901

TTCCT TTCTA TTCCA TTCCA TTTCA TTCGA TTTGA TTCCA TTCTG TTCTA TTCCA TTCAA TTCTT TTTCA 971

TTCCA TTCGA ATCCT TTCTA TTGCA GTCCA TTCCA TTCGA GTCCA TTCCA ATCCC TTCCA TTCCA TTCCA 1041

TTACA GTCCA TTCCA ATAGA TTCCA TTTCC TTGCC TTCCA TTCGA ATCCA TTCCA TTCTA GTCCA TTCCA 1111

TTTGA GTCAA TTCCA TTCCA TTCCA TTCTA TTCCT TTCCA ATCCA TTCGA TTCCA TTCGA TTCAA TTCCA 1181

TTTGA TTCTC TTTCA TTCTA TTTTA TTCCA TGCCA TTTGA TTGCA TTGCA TTCCA TTCCG TTTGA TTCCA 1251

GTCCA TTCAA GAAAG TTCCA TTACA GTCCA TTGCT TTCGA GTCCA TTCCA TTCCA CTCTA GTCTA TTCCA 1321

CTCCA TTCCT TTCAA TTCCA TTCCA TACTA TTCCA TTCCA TTCCT TTGCA TTCCG T---- TTCCA ATCTA 1387

TTCGA GTCCA TTGCA TTCCA GTCCA ATCCA TTCCA TTACA TTCCT TTTGA TTCCC TGCCA GTCGA TTGCA 1457

TTGCA TACTA CACCA TTCCA AAGGA GTCCA TTCCA TTCTA TTTCA ACACT TTCCA TTCCA CTCTG TTCGA 1527

GTCCA TTCCA TTCCA GTCCA TTTAA TTCAA GGGCA TTCCA TTCCA TTCCA TTCCA TTCCA TTTCA TATTA 1597

TTCCA TTCCA TTCAA TTCCA TTCCA GATGA TTCCA TTCCA TTCTA TACCA TTGCT CTCTG TTCCA TTCCA 1667

-TCCA TCTGT CTCCA TTCCT TTCGT TTCGA TTCCT TTCCA TTCCA TTCAA TTACA TTTGA TCCTA TTTTA 1736

TTAAA TTTCA TTCTA TTCGA GTGAT TTCCA TTCGA GTCCT TTCCA TTCGA TTCCA TTCCA TTCTA TTCCA 1806

TTCCT TTGGA TTCCA TTCCA TTCCG TTCCG TTCAC ATCAA TTCCT TGCGA TTCCA TTACA TTCGA TTTCT 1876

TGCCA TTCGA TTCCA TTCCT TTTGA CTCCA TTTCA TTCGA TTCCA TTCCA TTCCA TTAAT TTCCA TTCCA 1946

TTCGA GACCT TTCCA TTGCA GTCTT TTCCC TTCGA GTCCA TTCCG TTCGA TTCCC TTCCA TTTGA TTCCA 2016

TTCCA TTGGA GTCCG TACCA GTCGA GTCCA TTCTA TTCCA GTCCA TTAGT TTCGA CTCCA TTGCA TTCGA 2086

GTGCA TTCCA TTCCG TGGCT GTCCA TTCCA TTCCG TTTGA TGCCA TTCCA TACGA TTCCA TTCAA TTCGA 2156

GACCA TTCTA TTCCT GTCCA TTCCT TGTGG TTCGA TTCCA TTTCA CTCTA GTCCA TTCCA TTCCA TTCAA 2226

TTCCA TTCGA CTCTA TTCCG TTCCA TTCAA TTCCA TTCCA TTCGA TTCCA TTTTT TTCGA GAACC TTCCA 2296

TTACA CTCCC TTCCA TTCCA GTGCA TTCCA TTCCA GTCTC TTCAG TTCGA TTCCA TTCCA TTCGT TTCGA 2366

TTCCT TTCCA TTCCA GCCCA TTCCA TTCCA TTCCA TTCCT TTCCT TTCCG TTTCA TTAGA TTCCA TTGCA 2436

TTCGA TTCCA TTCAA TTCAA TTCCG TTCTA TTCAA TTTGA TTCAT TTCCA TTTAA TTCCA TTCCA TTAGA 2506

TTCCA TTCCT TTTGA ATCCA TTCCA TTGGA GTCCA TTCAC TTCCA GAACA TTCCA TTCCA GTCGA ATCCA 2576

TTCGA GTACA TTCCA TTAAA GTTCA TTACA TTCTA ATACA TTCCA TTCCA TTGCA TTCCA TTCCA TTCCA 2646

TTCGA TGCCA TTCGA TTCCA TTCCA TGCCA AATCA TTGCA TTCCT TTCCA TTCCG TTCCT ATCAA TTCCA 2716

TTCCA TTCGA TTTAG TTCGA TTCTA TTCAC TTCCA TTCCA TTCGA TTCCA TTCCA TTGGA GTCAA TTCCT 2786

TTCGA CACCC AGCCT TTCCA GTCAA TGATT TTGGA TTCCA TTTTG TTGCA TTCCA TTACA TTCTA TGACA 2856

TTCGA TTCCG TTTCA TTGAA TTCCA TTCCA TACAT TTTTA TTCCA TTCGA GACCG TAGCA TTCCA CTTTA 2926

TTCCA GG--- 2933

**>MZT3b (Blood)**

--CCT GTCCA TTACA CTACA TTCCC TTCCA TTCCA ATGAA TTCCA TTCCA TTCCA ATCCA TTCCT TTCCT 68

TTCGC TTGCA TTCCA TTCTA TTCTC TTCTA CTGCA TACAA TTTCA CTCCA TTCGT TCCCA TTCCA TTCAA 138

TTCCA TTCCA TTCAA TTCCA TTCCA TTTGT TTCCA TTCTC TTCGA TTCCA TTTCT TTATA TTCCA TGCCA 208

TTCGA TTCCA TTCTA TTGGA TTGCA TTACA TTCGT GTTCA TTCCA ATCCC ATCCA ATCCC ATCTA CTCCA 278

TTCAA TTCCT TTCCA TTCCA TTTGA TTTGA TTCCA TTGAT TTG-A TTCCA TTCAG TTTGA TTCCA TTCCG 347

TGAAA TTTCG TTCCA TTCTA TTCCA TTGCA TTACT TTCCA TTCAA TTCCA TTCCA TTTCA TTTCA GTCCA 417

TTTGC TTCCT TTCCT TTCGA TTCAA TTCCA TTTGA TTCCA CTCCA TTCTA TGCGA TTTCA TTCCA ATCGA 487

TTCAA TTCCA TTCGA TGACA TTCCT TTCGT TTCCA TTCCA TTCGA GTCCA TTTAA TTTGA –-GCA TTCGT 555

GTCCA TTCTA TTCGA GTCCA TTCCA TTACC GTCTA TTCTA TTCCC TTCCA TTCGT GTTGA TTCAA TTTCA 625

TTCCC TTCCA TTCGA TTCCT TTCCA TTCGA TTCCA TTCCT TTCCA TTCCA TTCCA TTCGT TCCCA TTCCA 695

TGTGA TTTCA TTCCA TTCCA GTCCA TTATA TTCGA GTCCA CTCCA CTCCA TTCTA TTACA TTCAA TTCCT 765

TTTGA GTCCG TTCCA TAACA CTCCA TTCAT TTCGA TTCCA TTTCT TGCCA GTTTT –-C-- TTCCA TTTTA 831

TTCCA TTCCG TTCGA TTCCA TTCCA TTCGA TTGCA TTCCA TTCGA ATCCT TTCCA TTCCA TTTCA TTCCA 901

TTCCT TTCTA TTCCA TTACA TTTCA TTCGA TTTGA TTCCA TTCTG TTCTA TTCCA TTCAA TTCTT TTTCA 971

TTCCA TTCGA ATCCT TTCTA TTGCA GTCCA TTCCA TTCGA GTCCA TTCCA ATCCC TTCCA TTCCA TTCCA 1041

TTACA GTCCA TTCCA ATAGA TTCCA TCCTT TGCC- TTCCA TTCGA ATCCA TTCCA TTCTA GTCCA TTCCA 1110

TTTGA GTCAA TTCCA TTCCA TTCCA TTCTA TTCCT TTCCA ATCCA TTCGA TTCCA TTCGA TTCAA TTCCA 1180

TTTGA TTCTC TTTCA TTCTA TTTTA TTCCA TGCCA TTTGA TTGCA TTGCA TTCCA TTCCG TTTGA TTCCA 1250

GTCCA TTCAA GAAAG TTCCA TTCCA GTCCA TTGCT TTCCA GTCCA TTCCA TTCCA CTCTA GTCTA TTCCA 1320

CTCCA TTCCT TTCCA TTCCA TTCCA TACTA TTCCA TTCCA TTCCT TTGCA TTCCG T---- TTCCA ATCTA 1386

TTCGA GTCCA TTGCA TTCCA GTCCA ATCCA TTCGA TTACA TTCGT TTTGA TTCCC TGCCA GTCGA TTGCA 1456

TTGCA TACTA GACCA TTCCA AAGGA GTCCA TTCCA TTCTA TCTCA ACACT TTCCA TTCCA CTCTG TTCGA 1526

GTCCA TTCCA TTCCA GTCCA TTTAA TTCAA GGGCA TTCCA TTCCA TTCCA TTCCA TTTCA TATTA TTCCA 1596

TTCCA TTCAA TTCCA TTCCA GATGA TTCCA TTCCA TTCTA TACCA TTGCT CTCTG TTCCA TTCCA TTCCA 1666

TCTGT CTCCA TTCCT TTCGT TTCGA TTCCT TTCCA TTCCA TTCCA TTACA TTTGA TCCTA TTTTA TTAAA 1736

TTGCA TTCTA TTCGA GTGAT TTCCA TTCGA GTCCT TTCCA TTCGA TTCCA TTCCA TTCTA TTCCA TTCCT 1806

TTGGA TTCCA TTCCA TTCCG TTCCG TTCAC ATCAA TTCCT TGCGA TTCCA TTACA TTCGA TTTCT TGCCA 1876

TTCGA TTCCA TTCCT TTTGA CTCCA TTTCA TTCGA TTCCA TTCCA TTCCA TTAAT TTCCA TTCCA TTCGA 1946

GACCA TTCCA TCGCA GTCTT TTCCC TTCGA GTCCA TTCCG TTCGA ATCCC TTCCA TTCGA TTCCA TTCCA 2016

TTGGA GTCCG TACCA GTCGA GTCCA TTCTA TTCCA GTCCA TTAGT TTCGA CTCCA TTGCA TTCGA GTGCA 2086

TTCCA TTCCG TGGCT GTCCA TTCCA TTCCG TTTGA TGCCA TTCCA TACGA TTCCA TTCAA TTCGA GACCA 2156

TTCTA TTCCT GTCCA TTCCT TGTGG TTCGA TTCCA TTTCA CTCTA GTCCA TTCCA TTCCA TTCAA TTCCA 2226

TTCGA CTCTA TTCCG TTCCA TTCAA TTCCA TTCCA TTCGA TTCCA TTTCT TTCGA GAACC TTCCA TTACA 2296

CTCCC TTCCA TTCCA GTGCA TTCCA TTCCA GTCTC TTCAG TTCGA TTCCA TTCCA TTCGT TTCGA TTCCT 2366

TTCCA TTCCA GCCCA TTCCA TTCCA TTCCA TTCCT TTCCT TTCCG TTTCA TTAGA TTCCA TTGCA TTCCA 2436

TTCCA TTCAA TTCAA TTCCG TGCTA TTCAA TTTGA TTCAT TTCCA TTTAA TTCCA TTCCA TTAGA TTCCA 2506

TTCCG TACGA TTCCA TTCCT TTTGA ATCCA TTCCA TTGGA GTCCA TTCAC TTCCA GAACA TTCCA TTCCA 2576

GTCGA ATCCA TTCGA GTACA TTCCA TTAAA GTTCA TTACA TTCTA ATACA TTCCA TTCCA TTGCA TTCCA 2646

TTCCA TTCCA TTCGA TGCCA TTCGA TTCCA TTCCA TGCCA AATCA TTGCA TTCCT TTCCA TTCCG TTCCT 2716

ATCAA TTCCA TTCCA TTCGA TTTAG TTCGA TTCTA TTCAC TTCCA TTCCA TTCGA TTCCA TTCCA TTGGA 2786

GTCAA TTCCT TTCGA CACCC AGCCT TTCCA GTCAA TGATA TTGGA TTCCA TTTTG TTGCA TTCCA TTACA 2856

TTCTA TGACA TTCGA TTCCA TTTCA TTGCA TTCCA TTCCA TACAT TTTTA TTCCA TTCGA GACCG TAGCA 2926

TTCCA CTTTA TTCCA GG--- 2943
