# Supplementary material for: DYZ1 arrays show sequence variation between the monozygotic males
Source: BMC Genet. 2014 Feb 4;15:19. doi: 10.1186/1471-2156-15-19 (PMC3925983; doi:10.1186/1471-2156-15-19)
Supplement: Additional file 2 — Multiple sequence alignment (MSA) of 3.56 Kb sequence of DYZ1 array from twin pairs. The regions of nucleotide variations are highlighted in yellow. (A) MZT1, (B) MZT2 and (C) MZT3. [file 1471-2156-15-19-S2.docx]

**Additional file 2:** Multiple sequence alignment (MSA) of 3.56 Kb sequence of DYZ1 array from twin pairs. The regions of nucleotide variations are highlighted in yellow. (A) MZT1, (B) MZT2 and (C) MZT3.

**(A)**

MZT1a CCTGTCCATTACACTACATTCCCTTCCATTCCAATGAATTCCATTCCATTCCAATCCATT 60

MZT1b CCTGTCCATTACACTACATTCCCTTCCATTCCAATGAATTCCATTCCATTCCAATCCATT 60

MZT1a CCTTTCCTTTCGCTTGCATTCCATTCTATTCCCTTCTACTGCATACAATTTCACTCCATT 120

MZT1b CCTTTCCTTTCGCTTGCATTCCATTCTATTCTCTTCTACTGCATACAATTTCACTCCATT 120

MZT1a CGTTCCCATTCCATTCAATTCCATTCCATTCAATTCCATTCCATTTGTTTCCATTCTCTT 180

MZT1b CGTTCCCATTCCATTCAATTCCATTCCATTCAATTCCATTCCATTTGTTTCCATTCTCTT 180

MZT1a CGATTCCATTTCTTTATATTCCATGCCATTCGATTCCATTCTATTGGATTGCATTACATT 240

MZT1b CGATTCCATTTCTTTATATTCCATGCCATTCGATTCCATTCTATTGGATTGCATTACATT 240

MZT1a CGTGTTCATTCCATTCCAGACCATTCCATTTGACTCCATTCCTTTCGAGCCCTTTCAATT 300

MZT1b CGTGTTCATTCCATTCCAGACCATTCCATTTGACTCCATTCCTTTCGAGCCCTTTCAATT 300

MZT1a TGAGTCCATTCCTTTCCAGTCCATTTCACTCCAGTCCATTACTATCCATTCCATACCATT 360

MZT1b TGAGTCCATTCCTTTCCAGTCCATTTCACTCCAGTCCATTACTATCCATTCCATACCATT 360

MZT1a CCATCCCATTCCATTCCATTCCATTCCATTCCATTCCATTCCATTCCATTGCATTCCATT 420

MZT1b CCATCCCATTCCATTCCATTCCATTCCATT--------------------GCATTCCATT 400

MZT1a CCATTCCATTCCATTGCACTGCACTCCATTCCATTACATTCTACTCTATCTGAGTCGATT 480

MZT1b CCATTCCATTCCATTGCACTGCACTCCATTCCATTACATTCTACTCTATCTGAGTCGATT 460

MZT1a TTATTGCATTAGATTCTATTCCATTGGATTACTTTCCATTCGATTACATTCCATTCATGT 540

MZT1b TTATTGCATTAGATTCTATTCCATTGGATTACTTTCCATTCGATTACATTCCATTCATGT 520

MZT1a ACATTCCATTCCAGTCAATTACATTCGAGTTCATTACATTACATTCCAGTATATTCCATT 600

MZT1b ACATTCCATTCCAGTCAATTACATTCGAGTTCATTACATTACATTCCAGTATATTCCATT 580

MZT1a GTATTCGATCCCATTCCTTTCAATTCCATTTCATTCGACTCCATTATATTCGATTCCATT 660

MZT1b GTATTCGATCCCATTCCTTTCAATTCCATTTCATTCGACTCCATTATATTCGATTCCATT 640

MZT1a CCACTCGAATCCATTCCATTAGAGGACATTCCATTCCAATGCATTCCATTCCATTCCATA 720

MZT1b CCACTCGAATCCATTCCATTAGAGGACATTCCATTCCAATGCATTCCATTCCATTCCATA 700

MZT1a GCATTCCATTGCATTCGATTCCATTCCATTTGATGCCATTCCATTTGATGCCATTCCATG 780

MZT1b GCATTCCATTGCATTCGATTCCATTCCATTTGATGCCATTCCATTTGATGCCATTCCATG 760

MZT1a ACATTCCATTCCATTCGAGTCCATTCCGTTCCAATTCATTCCATTCCGTTTCATGAAATT 840

MZT1b ACATTCCATTCCATTCGAGTCCATTCCGTTCCAATTCATTCCATTCCGTTTCATGAAATT 820

MZT1a CGAGTCCTTTCCAGTACATTTCATTCCAATCCCATCCAATCCCATCTACTCCATTCAATT 900

MZT1b CGAGTCCTTTCCAGTACATTTCATTCCAATCCCATCCAATCCCATCTACTCCATTCAATT 880

MZT1a CCTTTCCATTCCATTTGATTTGATTCCATTGATTTGATTCCATTCAGTTTGATTCCATTC 960

MZT1b CCTTTCCATTCCATTTGATTTGATTCCATTGATTTGATTCCATTCAGTTTGATTCCATTC 940

MZT1a CGTGAAATTTCGTTCCATTCTATTCCATTGCATTACTTTCCATTCAATTCCATTCCATTT 1020

MZT1b CGTGAAATTTCGTTCCATTCTATTCCATTGCATTACTTTCCATTCAATTCCATTCCATTT 1000

MZT1a CATTTCAGTCCATTCGCTTCCTTTCCTTTCGATTCAATTCCATTTGATTCCACTCCATTC 1080

MZT1b CATTTCAGTCCATTCGCTTCCTTTCCTTTCGATTCAATTCCATTTGATTCCACTCCATTC 1060

MZT1a TATGCGATTTCATTCCAATCGATTCAATTCCATTCGATGACATTCCTTTCGTTTCCATTC 1140

MZT1b TATGCGATTTCATTCCAATCGATTCAATTCCATTCGATGACATTCCTTTCGTTTCCATTC 1120

MZT1a CATTCGAGTCCATTTAATTTGAGCATTCGTGTCCATTCTATTCGAGTCCATTCCATTACA 1200

MZT1b CATTCGAGTCCATTTAATTTGAGCATTCGTGTCCATTCTATTCGAGTCCATTCCATTACA 1180

MZT1a GTCTATTCTATTCCCTTCCATTCCTGTTGATTCAATTTCATTCCCTTCCATTCGATTCCT 1260

MZT1b GTCTATTCTATTCCCTTCCATTCGTGTTGATTCAATTTCATTCCCTTCCATTCGATTCCT 1240

MZT1a TTCCATTCGATTCCATTCCTTTCCATTCCATTCCATTCGTTCCCATTCCATGTGATTTCA 1320

MZT1b TTCCATTCGATTCCATTCCTTTCCATTCCATTCCATTCGTTCCCATTCCATGTGATTTCA 1300

MZT1a TTCCATTCCAGTCCATTATATTCGAGTCCACTCCACTCCATTCTATTACATTCAATTCCT 1380

MZT1b TTCCATTCCAGTCCATTATATTCGAGTCCACTCCACTCCATTCTATTACATTCAATTCCT 1360

MZT1a TTTGAGTCCGTTCCATAACACTCCATTCATTTCGATTCCATTTCTTGCCAGTTTTCTTCC 1440

MZT1b TTTGAGTCCGTTCCATAACACTCCATTCATTTCGATTCCATTTCTTGCCAGTTTTCTTCC 1420

MZT1a ATTTTATTCCATTCCGTTCGATTCCATTCCATTCGATTGCATTCCATTCGAATCCTTTCC 1500

MZT1b ATTTTATTCCATTCCGTTCGATTCCATTCCATTCGATTGCATTCCATTCGAATCCTTTCC 1480

MZT1a ATTCCATTTCATTCCATTCCTTTCTATTCCATTCCATTTCATTCGATTTGATTCCATTCT 1560

MZT1b ATTCCATTTCATTCCATTCCTTTCTATTCCATTCCATTTCATTCGATTTGATTCCATTCT 1540

MZT1a GTTCTATTCCATTCAATTCTTTTTCATTCCATTCGAATCCTTTCTATTGCAGTCCATTCC 1620

MZT1b GTTCTATTCCATTCAATTCTTTTTCATTCCATTCGAATCCTTTCTATTGCAGTCCATTCC 1600

MZT1a ATTCGAGTCCATTCCAATCCCTTCCATTCCATTCCATTACAGTCCATTCCAATAGATTCC 1680

MZT1b ATTCGAGTCCATTCCAATCCCTTCCATTCCATTCCATTACAGTCCATTCCAATAGATTCC 1660

MZT1a ATTCCTTTGCCTTCCATTCGAATCCATTCCATTCTAGTCCATTCCATTTGAGTCAATTCC 1740

MZT1b ATTCCTTTGCCTTCCATTCGAATCCATTCCATTCTAGTCCATTCCATT------------ 1708

MZT1a ATTCCATTCCATTCTATTCCTTTCCAATCCATTCGATTCCATTCGATTCAATTCCATTTG 1800

MZT1b ---CCATTCCATTCTATTCCTTTCCAATCCATTCGATTCCATTCGATTCAATTCCATTTG 1765

MZT1a ATTCTCTTTCATTCTATTTTATTCCATGCCATTTGATTGCATTGCATTCCATTCCGTTTG 1860

MZT1b ATTCTCTTTCATTCTATTTTATTCCATGCCATTTGATTGCATTGCATTCCATTCCGTTTG 1825

MZT1a ATTCCAGTCCATTCAAGAAAGTTCCATTCCAGTCCATTGCTTTCCAGTCCATTCCATTCC 1920

MZT1b ATTCCAGTCCATTCAAGAAAGTTCCATTCCAGTCCATTGCTTTCCAGTCCATTCCATTCC 1885

MZT1a ACTCTAGTCTATTCCACTCCATTCCTTTCCATTCCATTCCATACTATTCCATTCCATTCC 1980

MZT1b ACTCTAGTCTATTCCACTCCATTCCTTTCCATTCCATTCCATACTATTCCATTCCATTCC 1945

MZT1a TTTGCATTCCGTTTCCAATCTATTCGAGTCCATTGCATTCCAGTCCAATCCATTCGATTA 2040

MZT1b TTTGCATTCCGTTTGCAATCTATTCGAGTCCATTTCATTCCAGTCCAATCCATTCGATTA 2005

MZT1a CATTCCTTTTGATTCCCTGCCAGTCGATTGCATTGCATACTAGACCATTCCAAAGGAGTT 2100

MZT1b CATTCCTTTTGATTCCCTGCCTGTCGATTGCATTGCATACTAGACCATTCCAAACGAGTC 2065

MZT1a CATTCCATTCTATCTCCACACTTTCCATTCCACTCTGTTTGAGTCCATTCCATTCCAGTC 2160

MZT1b CATTCCATTCTATCTCAACACTTTCCATTCCACTCTGTTCGAGTCCATTCCATTCCAGTC 2125

MZT1a CATTTAATTCAAGGGCATTCCATTCCATTCCATTCCATTCCATTTCATATTATTCCATTC 2220

MZT1b CATTTAATTCAAGGGCATTCCATTCCATTCCATTCCATTCCATTTCATATTATTCCATTC 2185

MZT1a CATTCAATTCCATTCCAGATGATTCCATTCCATTCTATACCATTGCTCTCTGTTCCATTC 2280

MZT1b CATTCAATTCCATTCCAGATGATTCCTTTCCATTCTATACCATTGCTCTCTGTTCTATTC 2245

MZT1a CATTCCATCTGTCTCCATTCCTTTCGTTTCGATTCCTTTCCATTCCATTCCATTACATTT 2340

MZT1b CATTCCATCTGTCTCCATTCCTTTCGTTTCGATTCCTTTCCATTCCATTCCATTACATTT 2305

MZT1a GATCCTATTTTATTAAATTGCATTCTATTCGAGTGATTTCCATTCGAGTCCTTTCCATTC 2400

MZT1b GATCCTATTTTATTAAGTTGCATTCTATTCGAGTGATTTCCATTCGAGTCCTTTCCATTC 2365

MZT1a GATTCCATTCCATTCTATTCCATTCCTTTGGATTCCATTCCATTCCGTTCCGTTCACATC 2460

MZT1b GATTCCATTCCATTCTATTCCATTCCTTTGGATTCCATTCCATTCCGTTCCGTTCACATC 2425

MZT1a AATTCCTTGCGATTCCATTACATTCGATTTCTTGCCATTCGATTCCATTCCTTTTGACTC 2520

MZT1b AATTCCTTGCGATTCCATTACATTCGATTTCTTGCCATTCGATTCCATTCCTTTTGACTC 2485

MZT1a CATTTCATTCGATTCCATTCCATTCCATTAATTTCCATTCCATTCGAGACCTTTCCATTG 2580

MZT1b CATTTCATTCGATTCCATTCCATTCCATTAATTTCCATTCCATTCGAGACCTTTCCATTG 2545

MZT1a CAGTCTTTTCCCTTCGAGTCCATTCCGTTCGATTCCCTTCCATTCGATTCCATTCCATTG 2640

MZT1b CAGTCTTTTCCCTTCGAGTCCATTCCGTTCGATTCCCTTCCATTCGATTCCCTTCCATTG 2605

MZT1a GAGTCCGTACCAGTCGAGTCCATTCTATTCCAGTCCATTAGTTTCGACTCCATTGCATTC 2700

MZT1b GAGTCCGTACCAGTCGAGTCCATTCTATTCCAGTCCATTAGTTTCGACTCCATTGCATTC 2665

MZT1a GAGTGCATTCCATTCCGTGGCTGTCCATTCCATTCCGTTTGATGCCATTCCATACGATTC 2760

MZT1b GAGTGCATTCCATTCCGTGGCTGTCCATTCCATTCCGTTTGATGCCATTCCATACGATTC 2725

MZT1a CATTCAATTCGAGACCATTCTATTCCTGTCCATTCCTTGTGGTTCGATTCCATTTCACTC 2820

MZT1b CATTCAATTCGAGACCATTCTATTCCTGTCCATTCCTTGTGGTTCGATTCCATTTCACTC 2785

MZT1a TAGTCCATTCCATTCCATTCAATTCCATTCGACTCTATTCCGTTCCATTCAATTCCATTC 2880

MZT1b TAGTCCATTCCATTCCATTCAATTCCATTCGACTCTATTCCGTTCCATTCAATTCCATTC 2845

MZT1a CATTCGATTCCATTTTTTTCGAGAACCTTCCATTACACTCCCTTCCATTCCAGTGCATTC 2940

MZT1b CATTCGATTCCATTTTTTTCGAGAACCTTCCATTACACTCCCTTCCATTCCAGTGCATTC 2905

MZT1a CATTCCAGTCTCTTCAGTTCGATTCCATTCCATTCGTTTCGATTCCTTTCCATTCCAGCC 3000

MZT1b CATTCCAGTCTCTTCAGTTCGATTCCATTCCATTCGTTTCGATTCCTTTCCATTCCAGCC 2965

MZT1a CATTCCATTCCATTCCATTCCTTTCCTTTCCGTTTCATTAGATTCCATTGCATTCGATTC 3060

MZT1b CATTCCATTCCATTCCATTCCTTTCCTTTCCGTTTCATTAGATTCCATTGCATTCGATTC 3025

MZT1a CATTCAATTCAATTCCGTGCTATTCAATTTGATTCATTTCCATTTAATTCCATTCCATTA 3120

MZT1b CATTCAATTCAATTCCGTGCTATTCAATTTGATTCATTTCCATTTAATTCCATTCCATTA 3085

MZT1a GATTCCATTCCGTACGATTCCATTCCTTTTGAATCCATTCCATTGGAGTCCATTCACTTC 3180

MZT1b GATTCCATTCCGTACGATTCCATTCCTTTTGAATCCATTCCATTGGAGTCCATTCACTTC 3145

MZT1a CAGAACATTCCATTCCAGTCGAATCCATTCGAGTACATTCCATTAAAGTTCATTACATTC 3240

MZT1b CAGAACATTCCATTCCAGTCGAATCCATTCGAGTACATTCCATTAAAGTTCATTACATTC 3205

MZT1a TAATACATTCCATTCCATTGCATTCCATTCCATTCCATTCGATGCCATTCGATTCCATTC 3300

MZT1b TAATACATTCCATTCCATTGCATTCCATTCCATTCCATTCGATGCCATTCGATTCCATTC 3265

MZT1a CATGCCAAATCATTGCATTCCTTTCCATTCCGTTCCTATCAATTCCATTCCATTCGATTT 3360

MZT1b CATGCCAAATCATTGCATTCCTTTCCATTCCGTTCCTATCAATTCCATTCCATTCGATTT 3325

MZT1a AGTTCGATTCTATTCACTTCCATTCCATTCGATTCCATTCCATTGGAGTCAATTCCTTTC 3420

MZT1b AGTTCGATTCTATTCACTTCCATTCCATTCGATTCCATTCCATTGGAGTCAATTCCTTTC 3385

MZT1a GACACCCAGCCTTTCCAGTCAATGATTTTGGATTCCATTTTTTTGCATTCCATTACATTC 3480

MZT1b GACACCCAGCCTTTCCAGTCAATGATTTTGGATTCCATTTTTTTGCATTCCATTACATTC 3445

MZT1a TATGACATTCGATTCCGTTTCATTGCATTCCATTCCATACATTTTTATTCCATTCGAGAC 3540

MZT1b TATGACATTCGATTCCGTTTCATTGCATTCCATTCCATACATTTTTATTCCATTCGAGAC 3505

MZT1a CGTAGCATTCCACTTTATTCCAGG 3564

MZT1b CGTAGCATTCCACTTTATTCCAGG 3529

**(B)**

MZT2a CCTGTCCATTACACTACATTCCCTTCCATTCCCATGAATTCCATTCCATTCCAATCCATT 60

MZT2b CCTGTCCATTACACTACATTCCCTTCCATTCCAATGAATTCCATTCCATTCCAATCCATT 60

MZT2a CCTTTCCTTTCGCTTGCATTCCACTCTTTTCTCTTCTACTGCATACAATTTCACTACATT 120

MZT2b CCTTTCCTTTCGCTTGCATTCCATTCTATTCTCTTCTACTGCATACAATTTCCCTCCATT 120

MZT2a CGTTCCCATTCCATTCAATTCCATTCCATTCAATTCCATTCCGTTTGTTTCCATTCTCTT 180

MZT2b CGTTCCCATTCCATTCAATTCCATTCCATTCAATTCCATTCCATTTGTTTCCATTCTCTT 180

MZT2a CGATTCCATTTCTTTATATTCCATGCCATTCGATTCCATTCTATTGGATTGCATTACATT 240

MZT2b CGATTCCATTTCTTTATATTCCATGCCATTCGATTCCATTCTATTGGGTTGCATTACATT 240

MZT2a CGTGTTCATTCCATTCCAGACCATTCCATTTGACTCCATTCCTTTCGAGCCCTTTCAATT 300

MZT2b CGTGTTCATTCCATTCCAGACCATTCCATTTGACTCCATTCCTTTCGAGCCCTTTCAATT 300

MZT2a TGAGTCCATTCCTTTCCAGTCCATTTCCCTCCAGTCCATTACTATCCATTCCATACCATT 360

MZT2b TGAGTCCATTCCTTTCCAGTCCATTTCACTCCAGTCCATTACTATCCATTCCATACCATT 360

MZT2a CCATCCCATTCCATTCCATTCCATTCCATTCCATTGCATTCCATTCCATT---------- 410

MZT2b CCATCCCATTCCATTCCATTCCATTCCATTCCATTCCATTCCATTCCATTGCATTCCATT 420

MZT2a ------CCATTCCATTGCACTGCACTCCATTCCATTACATTCTACTCTATTTGAGTCGAT 464

MZT2b CCATTCCCATTCCATTGCACTGCACTCCATTCCATTACATTCTACTCTATCTGAGTCGAT 480

MZT2a TTTATTGCATTAGATTCTATTCCATTGGATTGCTTTCCATTCGATTACATTCCATTCATG 524

MZT2b TTTATTGCATTAGATTCTATTCCATTGGATTACTTTCCATTCGATTACATTCCATTCATG 540

MZT2a TACATTCCATTCCAGTCAATTACATTCGAGTTCATTACATTACATTCCAGTATATTCCAT 584

MZT2b TACATTCCATTCCAGTCAATTACATTCGAGTTCATTACATTACATTCCAGTATATTCCAT 600

MZT2a TGTATTCGGTCCCATTCCTTTCAATTCCCTTTGATTAGACTCCATTATATTCGATTCCAT 644

MZT2b TGTATTCGATCCCACTCCTTTCAATTCCATTTCTTTCGACTCCATTATATTCGATTCCAT 660

MZT2a TCCACTCGAATACATTCCATTAGAGGACATTCCATTCCAATGCATTCCATTCCATTCCAT 704

MZT2b TCCACTCGAATCCATTCCATTAGAGGACATTCCATTCCAATGCATTCCATTCCATTCCAT 720

MZT2a AGCATTCCATCGCATTCGATTCCATTCCATTTGATGCCATTCCAT--------------- 749

MZT2b AGCATTCCATTGCATTCGATTCCATTCCATTTGATGCCATTCCATTTGATGCCATTCCAT 780

MZT2a GACATTCCATTCCATTCGAGTCCATTCCGTTCTAATTCATTCCTTTCCGTTTCATGAAAT 809

MZT2b GACATTCCATTCCATTCGAGTCCATTCCGTTCCAATTCATTCCATTCCGTTTCATGAAAT 840

MZT2a TCGAGTCCTTTCCAGTACATTTCATTCCAATCCCATCCAATCCCATCTACTCCATTCAAT 869

MZT2b TCGAGTCCTT-CCAGTACATTTCATTCCAATCCCATCCAATCCCATCTACTCCATTCAAT 899

MZT2a TCCTTTCCATTCCATTTGATTTGATTCCATTGATTTGATTCCATTCAGTTTGATTCCGTT 929

MZT2b TCCTTTCCATTCCATTTGATTTGATTCCATTGATTTGATTCCATTCAGTTTGATTCCATT 959

MZT2a CCGTGAAATTTCGTTCCATTCTATTCCATTGCATTACTTTCCATTCAATTCCATTCCATT 989

MZT2b CCGTGAAATTTCGTTCCATTCTATTCCATTGCATTACTTTCCATTCAATTCCATTCCATT 1019

MZT2a TCATTTCAGTCCATTCGCTTCCTTTCCTTTCGATTCAATTCCATTTGATTCCACTCCATT 1049

MZT2b TCATTTCAGTCCATTCGCTTCCTTTCCTTTCGATTCAATTCCATTTGATTCCACTCCATT 1079

MZT2a CTATGCGATTTCATTCCAATCGATTCAATTCCATTCGATAACATTCCTTTCGTTTCCATT 1109

MZT2b CTATGCGATTTCATTCCAATCGATTCAATTCCATTCGATGACATTCCTTTCGTTTCCATT 1139

MZT2a CAATTCGAGTCCATTTAATTTGAGCATTCGTGTCCATTCTATCCGAGTCCATTCCATTAC 1169

MZT2b CCATTCGAGTCCATTTAATTTGAGCATTCGTGTCCATTCTATTCGAGTCCATTCCATTAC 1199

MZT2a CGTCTATTCTATTCCCTTCCATTCCTGTTGATTCAATTTCATTCCCTTCCATTCGATTCC 1229

MZT2b AGTCTATTCTATTCCCTTCCATTCCTGTTGATTCAATTTCATTCCCTTCCATTCGATTCC 1259

MZT2a TTTCCATTGGATTCCATTCCTTTCCATTCCATTCCATTCGTTCCCATTCCATGTGATTTC 1289

MZT2b TTTCCATTCGATTCCATTCCTTTCCATTCCATTCCATTCGTTCCCATTCCATGTGATTTC 1319

MZT2a ATTCCATTCCAGTCCATTATATTCGAGTCCACTCCACTCAATTCTATTACATTCAATTCC 1349

MZT2b ATTCCATTCCAGTCCATTATATTCGAGTCCACTCCACTCCATTCTATTACATTCAATTCC 1379

MZT2a TTTTGAGTCCGTTCCATAACACTCCATTCATTTCGATTCCATTTCTTGCCAGTTTTCTTC 1409

MZT2b TTTTGATTCCGTTCCATAACACTCCATTCATTTCGATTCCATTTCTTGCCAGTTTTCTTC 1439

MZT2a CATTTTATTCCATTCCGTTCGATTCCATTCCATTCGATTGCATTCCATTCGAATCCTTTC 1469

MZT2b CATTTTATTCCATTCCGTTCGATTCCATTCCATTCGATTGCATTCCATTCGAATCCTTTC 1499

MZT2a CATTCCATTTCATTCCATTCCTTTCTATTCCATTCCATTTCATTCGATTTGATTCCATTC 1529

MZT2b CATTCCATTTCATTCCATTCCTTTCTATTCCATTCCATTTCATTCGATTTGATTCCATTC 1559

MZT2a TGTTCTATTCCATTCAATTCTTTTTCATTCCATTCGAATCCTTTCTATTGCAGTCCATTC 1589

MZT2b TGTTCTATTCCATTCAATTCTTTTTCATTCCATTCGAATCCTTTCTATTGCAGTCCATTC 1619

MZT2a CATTCGAGTCCATTCCAATCCCTTCCATTCCATTCCATTACAGTCCATTCCAATAGATTC 1649

MZT2b CATTCGAGTCCATTCCAATCCCTTCCATTCCATTCCATTACAGTCCATTCCAATAGATTC 1679

MZT2a CATTCCTTTGCCTTCCATTCGAATCCATTCCATTCTAGTCCATTCCATTTGAGTCAATTC 1709

MZT2b CATTCCTTTGCCTTCCATTCGAATCCATTCCATTCTAGTCCATTCCATTTGAGTCAATTC 1739

MZT2a CATTCCATTCCATTCTATTCCTTTCCAATCCATTCGATTCCATTCGATTCAATTCCATTT 1769

MZT2b CATTCCATTCCATTCTATTCCTTTCCAATCCATTCGATTCCATTCGATTCAATTCCATTT 1799

MZT2a GATTCTCTTTCATTCTATTTTATTCCATGCCATTTGATTGCATTGCATTCCATTCCGTTT 1829

MZT2b GATTCTCTTTCATTCTATTTTATTCCATGACATTTGATTGCATTGCATTCCATTCCGTTT 1859

MZT2a GATTCCAGTCCATTCAAGAAAGTTCCATTCCAGTCCATTGCTTTCCAGTCCATTCCATTC 1889

MZT2b GATTCCAGTCCATTCAAGAAACTTCCATTCCAGTCCATTGCTTTCGAGTCCATTCCATTC 1919

MZT2a CACTCTAGTCTATTCCACTCCATTCCTTTCCATTCCATTCCATACTATTCCATTCCATTC 1949

MZT2b CACTCTAGTCTATTCCACTCCATTCCTTTGCATTCCATTCCATACTATTCCATTCCATTC 1979

MZT2a CTTTGCATTCCGTTTGCAATCTATTCGAGTCCATTGCATTCCAGTCCAATCCATTCGATT 2009

MZT2b CTTTGCTTTCCGTTTCCAATCTATTCGAGTCCATTGCATTCCAGTCCAATCCATTCGATT 2039

MZT2a ACATTCCTTTTGATTCCCTGCCAGTCGATTGCATTGCATACTAGACCATTCCAAAGGAGT 2069

MZT2b ACATTCCTTTTGATTCCCTGCCAGTTGATTGCATTGCATACTAGACCATTCCAAACGAGT 2099

MZT2a CCATTCCATTATATTTCAACACTTTCCATTCCACTCTGTTCGAGTCCATTCCATTCCAGT 2129

MZT2b CCATTCCATTCTATTTCAACACTTTCCATTCCACTCTGTTCGAGTCCATTGCATTCCAGT 2159

MZT2a CCATTTAATTCAAGGGCATTCCATTCCATTCCATTCCATTTCATGTTATTCCATTCCATT 2189

MZT2b CCATTTAATTCAAGGGCATTCCATTCCATTCCATTCCATTTCATATTATTCCATTCCATT 2219

MZT2a CAATTCCATTCCAGATGATTCCATTCCATTCTATACCATTGCTCTCTGTTCCATTCCATT 2249

MZT2b CAATTCCATTCCAGATGATTCCATTCCATTCTATACCATTGCTCTCTGTTCCATTCCATT 2279

MZT2a CCATCTGTCTCCATTCCTTTCGTTTCGATTCCTTTCCATTCCATTCCATTACATTTGATC 2309

MZT2b CCATCTGTCTCCATTCCTTTCGTTTCGATTCCTTTCCATTCCATTCCATTACATTTCATC 2339

MZT2a CTATTTTATTAAATTGCATTCTATTCGAGTGATTTCCATTCGAGTCCTTTCCATTCGATT 2369

MZT2b GTATTTTATTCAATTGCATTCTATTCGAGTGATTTCCATTCGAGTTCTTTCCATTCGATT 2399

MZT2a CCATTCCATTCTATTCCATTCCTTTGGATTCCATTCCATTCCGTTCCGTTCACATCAATT 2429

MZT2b CCATTCCATTCTATTCCATTTCTTTGGATTCCATTCCATTCCGTTCCGTTCACATCAATT 2459

MZT2a CCTTGCGATTCCATTACATTTGATTTCTTGCCATTCGATTCCATTCCTTTTGACTCCATT 2489

MZT2b CCTTGCGATTCCATTACATTCGATTTCTTGCCATTCGATTCCATTCCTTTTGACTCCATT 2519

MZT2a TCATTCGATTCCAATCCATTCCATTAATTTCCATTCCATTCGAGACCTTTCCATTGCAGT 2549

MZT2b TCATTCGATTCCATTCCATTCCATTAATTTCCATTCCATTCGAGACCTTTCCATTGCAGT 2579

MZT2a CTTTTCCCTTCGAGTCCATTCCGTTCGATTCCCTTGCATTCGATTCCATTCCATTGGAGT 2609

MZT2b CTTTTCCCTTCGAGTCCATTCCGTTCGATTCCCTTCCATTCGATTCCATTCCATTGGAGT 2639

MZT2a CCGTACCAGTCGAGTCCATTCTATTCCAGTCCATTAGTTTCGACTCCATTGCATTCGAGT 2669

MZT2b CCGTACCAGTCGAGTCCATTCTATTCCAGTCCATTAGTTTCGACTCCATTGCATTCGAGT 2699

MZT2a GCATTCCATTCCGTGGCTGTCCATTCCATTCCGTTTGATGCCATTCCATACGATTCCATT 2729

MZT2b GCATTCCATTCCGTGGCTGTCCATTCCATTCCGTTTGATGCCATTCCATACGATTCCATT 2759

MZT2a CAATTCGAGACCATTCTATTCCTGTCCATTCCTTGTGGTTCGATTCCATTTCACTCTAGT 2789

MZT2b CAATTCGAGACCATTCTATTCCTGTCCATTCCTTGTGGTTCGATTCCATTTCACTCTAGT 2819

MZT2a CCATTCCATTCCATTCAATTCCATTCGACTCTATTCCGTTCCATTCAATTCCATTCCATT 2849

MZT2b CCATTCCATTCCATTCAATTCCATTCGACTCTATTCCGTTCCATTCAATTGCATTCCATT 2879

MZT2a CGATTCCATTTTTTTCGAGAACCTTCCATTACACTCCCTTCCATTCCAGTGCATTCCATT 2909

MZT2b CGATTCCATTTTTTTCGAGAACCTTCCATTACACTCCCTTCCATTCCAGTGCATTCCATT 2939

MZT2a CCAGTCTCTTCAGTTCGATTCCATTCCATTCGTTTCGATTCCTTTCCATTCCAGCCCATT 2969

MZT2b CCAGTCTCTTCAGTTCGATTCCATTCCATTCGTTTCGATTCCTTTCCATTCCAGTCCATT 2999

MZT2a CCATTCCATTCCATTCCTTTCCTTTCCGTTTCATTAGATTCCATTGCATTCCATTCCATT 3029

MZT2b CCATTCCATTCCATTCCTTTCCTTTCCGTTTCATTAGATTCCATTGCATTCGATTCCATT 3059

MZT2a CAATTCAATTCCGTGCTATTCAATTTGATTCATTTCCATTTAATTCCATTCCATTAGATT 3089

MZT2b CAATTCAATTCCGTGCTATTCAATTTGATTCATTTCCATTTAATTCCATTCCATTAGATT 3119

MZT2a CCATTCCGTACGATTCCATTCCTTTTGAATCCATTCCATTGGAGTCCATTCACTTCCAGA 3149

MZT2b CCATTCCGTACGATTCCATTCCTTTTGAATCCATTCCATTGGAGTCCATTCACTTCCAGA 3179

MZT2a ACATTCCATTCCAGTCGAATCCATTCGAGTACATTCCATTAAAGTTCATTACATTCTAAT 3209

MZT2b ACATTCCATTCCAGTCGAATCCATTCGAGTACATTCCATTAAAGTTCATTACATTCTAAT 3239

MZT2a ACATTCCATTCCATTGCATTCCATTCCATTCCATTCGATGCCATTCGATTCCATTCCATG 3269

MZT2b ACATTCCATTCCATTGCATTCCATTCCATTCCATTCGATGCCATTCGATTCCATTCCATG 3299

MZT2a CCAAATCATTGCATTCCTTTCCATTCCGTTCCTATCAATTCCATTCCATTCGATTTAGTT 3329

MZT2b CCAAATCATTGCAATCCTTTCCATTCCGTTCCTATCAATTCCATTCCATTCGATTTAGTT 3359

MZT2a CGATTCTATTCACTTCCATTCCATTCGATTCCATTCCATTGGAGTCAATTCCTTTCGACA 3389

MZT2b CGATTCTATTCACTTCCATTCCATTCGATTCCATTCCATTGGAGTCAATTCCTTTCGACA 3419

MZT2a CCCAGCCTTTCCAGTCAATGATTTTGGATTCCATTTTTTTGCATTCCATTACATTCTATG 3449

MZT2b CCCAGCCTTTCCATTCAATGATTTTGGATTCCATTTTTTTGCATTCCATTACATTCTATG 3479

MZT2a ACATTCGATTCCGTTTCATTGCATTCCATTCCATACATTTTTATTCCATTCGAGACCGTA 3509

MZT2b ACATTCGATTCCGTTTCATTGCATTCCATTCCATACATTTTTATTCCATTCGAGACCGTA 3539

MZT2a GCATTCCACTTTATTCCAGG 3529

MZT2b GCATTCCACTTTATTCCAGG 3559

**(C)**

MZT3a CCTGTCCATTACACTACATTCCCTTCCATTCCAATGAATTCCATTCCATTCCAATCCATT 60

MZT3b CCTGTCCATTACACTACATTCCCTTCCATTCCAATGAATTCCATTCCATTCCAATCCATT 60

MZT3a CCTTTCCTTTCGCTTGCTTTACATTCTATTCCCTTCTACTGCATACAATTTCACTCCATT 120

MZT3b CCTTTCCTTTCGCTTGCATTCCATTCTATTCTCTTCTACTGCATACAATTTCACTCCATT 120

MZT3a CGTTCCCATTCCATTCAATTCCATTCCATTCAATTCCATTCCATTTGTTTCCATTCTCTT 180

MZT3b CGTTCCCATTCCATTCAATTCCATTCCATTCAATTCCATTCCATTTGTTTCCATTCTCTT 180

MZT3a CGATTCCATTTCTTTATATTCCATGCCATTCGATTCCATTCTATTGGATTGCATTACATT 240

MZT3b CGATTCCATTTCTTTATATTCCATGCCATTCGATTCCATTCTATTGGATTGCATTACATT 240

MZT3a CGTGTTCATTCCAATCCCATCCAATCCCATCTACTCCATTCAATTCCTTTCCATTCCATT 300

MZT3b CGTGTTCATTCCAATCCCATCCAATCCCATCTACTCCATTCAATTCCTTTCCATTCCATT 300

MZT3a TGATTTGATTCCATTGATTTGATTCCATTCAGTTTGATTCCATTCCGTGAAATTTCGTTC 360

MZT3b TGATTTGATTCCATTGATTTGATTCCATTCAGTTTGATTCCATTCCGTGAAATTTCGTTC 360

MZT3a CATTCTATTCCATTGCATTACTTTCCATTCAATTCCATTCCATTTCATTTCAGTCCATTC 420

MZT3b CATTCTATTCCATTGCATTACTTTCCATTCAATTCCATTCCATTTCATTTCAGTCCATTT 420

MZT3a GCTTCCTTTCCTTTCGATTCAATTCCATTTGATTCCACTCCATTCTATGCGATTTCATTC 480

MZT3b GCTTCCTTTCCTTTCGATTCAATTCCATTTGATTCCACTCCATTCTATGCGATTTCATTC 480

MZT3a CAATCGATTCAATTCCATTCGGTGACATTCCTTTCGTTTCCATTCCATTCGAGTCCATTT 540

MZT3b CAATCGATTCAATTCCATTCGATGACATTCCTTTCGTTTCCATTCCATTCGAGTCCATTT 540

MZT3a AATTTGAGCATGCGTGTCCATTCTATTCGAGTCCATTCCATTACCGTCTATTCTATTCCC 600

MZT3b AATTTGAGCATTCGTGTCCATTCTATTCGAGTCCATTCCATTACCGTCTATTCTATTCCC 600

MZT3a TTCCATTCCTGTTGATTCAATTTCATTCCCTTCCATTCGATTCCTTTCCATTCGATTCCA 660

MZT3b TTCCATTCGTGTTGATTCAATTTCATTCCCTTCCATTCGATTCCTTTCCATTCGATTCCA 660

MZT3a TTCCTTTCCATTCCATTCCATTCGTTCCCATTCCATGTGATTTCATTCCATTCCAGTCCA 720

MZT3b TTCCTTTCCATTCCATTCCATTCGTTCCCATTCCATGTGATTTCATTCCATTCCAGTCCA 720

MZT3a TTATATTCGAGTCCACTCCACTCCATTCTATTACATTCAATTCCTTTTGAGTCCGTTCCA 780

MZT3b TTATATTCGAGTCCACTCCACTCCATTCTATTACATTCAATTCCTTTTGAGTCCGTTCCA 780

MZT3a TAACACTCCATTCATTTCGATTCCATTTCTTGCCAGTTTTCTTCCATTTTATTCCATTCC 840

MZT3b TAACACTCCATTCATTTCGATTCCATTTCTTGCCAGTTTTCTTCCATTTTATTCCATTCC 840

MZT3a GTTCGATTCCATTCCATTCGATTGCATTCCATTCGAATCCTTTCCATTCCATTTCATTCC 900

MZT3b GTTCGATTCCATTCCATTCGATTGCATTCCATTCGAATCCTTTCCATTCCATTTCATTCC 900

MZT3a ATTCCTTTCTATTCCATTCCATTTCATTCGATTTGATTCCATTCTGTTCTATTCCATTCA 960

MZT3b ATTCCTTTCTATTCCATTACATTTCATTCGATTTGATTCCATTCTGTTCTATTCCATTCA 960

MZT3a ATTCTTTTTCATTCCATTCGAATCCTTTCTATTGCAGTCCATTCCATTCGAGTCCATTCC 1020

MZT3b ATTCTTTTTCATTCCATTCGAATCCTTTCTATTGCAGTCCATTCCATTCGAGTCCATTCC 1020

MZT3a AATCCCTTCCATTCCATTCCATTACAGTCCATTCCAATAGATTCCATTTCCTTGCCTTCC 1080

MZT3b AATCCCTTCCATTCCATTCCATTACAGTCCATTCCAATAGATTCCATC-CTTTGCCTTCC 1079

MZT3a ATTCGAATCCATTCCATTCTAGTCCATTCCATTTGAGTCAATTCCATTCCATTCCATTCT 1140

MZT3b ATTCGAATCCATTCCATTCTAGTCCATTCCATTTGAGTCAATTCCATTCCATTCCATTCT 1139

MZT3a ATTCCTTTCCAATCCATTCGATTCCATTCGATTCAATTCCATTTGATTCTCTTTCATTCT 1200

MZT3b ATTCCTTTCCAATCCATTCGATTCCATTCGATTCAATTCCATTTGATTCTCTTTCATTCT 1199

MZT3a ATTTTATTCCATGCCATTTGATTGCATTGCATTCCATTCCGTTTGATTCCAGTCCATTCA 1260

MZT3b ATTTTATTCCATGCCATTTGATTGCATTGCATTCCATTCCGTTTGATTCCAGTCCATTCA 1259

MZT3a AGAAAGTTCCATTACAGTCCATTGCTTTCGAGTCCATTCCATTCCACTCTAGTCTATTCC 1320

MZT3b AGAAAGTTCCATTCCAGTCCATTGCTTTCCAGTCCATTCCATTCCACTCTAGTCTATTCC 1319

MZT3a ACTCCATTCCTTTCAATTCCATTCCATACTATTCCATTCCATTCCTTTGCATTCCGTTTC 1380

MZT3b ACTCCATTCCTTTCCATTCCATTCCATACTATTCCATTCCATTCCTTTGCATTCCGTTTC 1379

MZT3a CAATCTATTCGAGTCCATTGCATTCCAGTCCAATCCATTCCATTACATTCCTTTTGATTC 1440

MZT3b CAATCTATTCGAGTCCATTGCATTCCAGTCCAATCCATTCGATTACATTCGTTTTGATTC 1439

MZT3a CCTGCCAGTCGATTGCATTGCATACTACACCATTCCAAAGGAGTCCATTCCATTCTATTT 1500

MZT3b CCTGCCAGTCGATTGCATTGCATACTAGACCATTCCAAAGGAGTCCATTCCATTCTATCT 1499

MZT3a CAACACTTTCCATTCCACTCTGTTCGAGTCCATTCCATTCCAGTCCATTTAATTCAAGGG 1560

MZT3b CAACACTTTCCATTCCACTCTGTTCGAGTCCATTCCATTCCAGTCCATTTAATTCAAGGG 1559

MZT3a CATTCCATTCCATTCCATTCCATTCCATTTCATATTATTCCATTCCATTCAATTCCATTC 1620

MZT3b CATTCCATTCCATTCCATTC-----CATTTCATATTATTCCATTCCATTCAATTCCATTC 1614

MZT3a CAGATGATTCCATTCCATTCTATACCATTGCTCTCTGTTCCATTCCAT-CCATCTGTCTC 1679

MZT3b CAGATGATTCCATTCCATTCTATACCATTGCTCTCTGTTCCATTCCATTCCATCTGTCTC 1674

MZT3a CATTCCTTTCGTTTCGATTCCTTTCCATTCCATTCAATTACATTTGATCCTATTTTATTA 1739

MZT3b CATTCCTTTCGTTTCGATTCCTTTCCATTCCATTCCATTACATTTGATCCTATTTTATTA 1734

MZT3a AATTTCATTCTATTCGAGTGATTTCCATTCGAGTCCTTTCCATTCGATTCCATTCCATTC 1799

MZT3b AATTGCATTCTATTCGAGTGATTTCCATTCGAGTCCTTTCCATTCGATTCCATTCCATTC 1794

MZT3a TATTCCATTCCTTTGGATTCCATTCCATTCCGTTCCGTTCACATCAATTCCTTGCGATTC 1859

MZT3b TATTCCATTCCTTTGGATTCCATTCCATTCCGTTCCGTTCACATCAATTCCTTGCGATTC 1854

MZT3a CATTACATTCGATTTCTTGCCATTCGATTCCATTCCTTTTGACTCCATTTCATTCGATTC 1919

MZT3b CATTACATTCGATTTCTTGCCATTCGATTCCATTCCTTTTGACTCCATTTCATTCGATTC 1914

MZT3a CATTCCATTCCATTAATTTCCATTCCATTCGAGACCTTTCCATTGCAGTCTTTTCCCTTC 1979

MZT3b CATTCCATTCCATTAATTTCCATTCCATTCGAGACCATTCCATCGCAGTCTTTTCCCTTC 1974

MZT3a GAGTCCATTCCGTTCGATTCCCTTCCATTTGATTCCATTCCATTGGAGTCCGTACCAGTC 2039

MZT3b GAGTCCATTCCGTTCGAATCCCTTCCATTCGATTCCATTCCATTGGAGTCCGTACCAGTC 2034

MZT3a GAGTCCATTCTATTCCAGTCCATTAGTTTCGACTCCATTGCATTCGAGTGCATTCCATTC 2099

MZT3b GAGTCCATTCTATTCCAGTCCATTAGTTTCGACTCCATTGCATTCGAGTGCATTCCATTC 2094

MZT3a CGTGGCTGTCCATTCCATTCCGTTTGATGCCATTCCATACGATTCCATTCAATTCGAGAC 2159

MZT3b CGTGGCTGTCCATTCCATTCCGTTTGATGCCATTCCATACGATTCCATTCAATTCGAGAC 2154

MZT3a CATTCTATTCCTGTCCATTCCTTGTGGTTCGATTCCATTTCACTCTAGTCCATTCCATTC 2219

MZT3b CATTCTATTCCTGTCCATTCCTTGTGGTTCGATTCCATTTCACTCTAGTCCATTCCATTC 2214

MZT3a CATTCAATTCCATTCGACTCTATTCCGTTCCATTCAATTCCATTCCATTCGATTCCATTT 2279

MZT3b CATTCAATTCCATTCGACTCTATTCCGTTCCATTCAATTCCATTCCATTCGATTCCATTT 2274

MZT3a TTTTCGAGAACCTTCCATTACACTCCCTTCCATTCCAGTGCATTCCATTCCAGTCTCTTC 2339

MZT3b CTTTCGAGAACCTTCCATTACACTCCCTTCCATTCCAGTGCATTCCATTCCAGTCTCTTC 2334

MZT3a AGTTCGATTCCATTCCATTCGTTTCGATTCCTTTCCATTCCAGCCCATTCCATTCCATTC 2399

MZT3b AGTTCGATTCCATTCCATTCGTTTCGATTCCTTTCCATTCCAGCCCATTCCATTCCATTC 2394

MZT3a CATTCCTTTCCTTTCCGTTTCATTAGATTCCATTGCATTCGATTCCATTCAATTCAATTC 2459

MZT3b CATTCCTTTCCTTTCCGTTTCATTAGATTCCATTGCATTCCATTCCATTCAATTCAATTC 2454

MZT3a CGTTCTATTCAATTTGATTCATTTCCATTTAATTCCATTCCATTAGATTCCATTCC---- 2515

MZT3b CGTGCTATTCAATTTGATTCATTTCCATTTAATTCCATTCCATTAGATTCCATTCCGTAC 2514

MZT3a -----------TTTTGAATCCATTCCATTGGAGTCCATTCACTTCCAGAACATTCCATTC 2564

MZT3b GATTCCATTCCTTTTGAATCCATTCCATTGGAGTCCATTCACTTCCAGAACATTCCATTC 2574

MZT3a CAGTCGAATCCATTCGAGTACATTCCATTAAAGTTCATTACATTCTAATACATTCCATTC 2624

MZT3b CAGTCGAATCCATTCGAGTACATTCCATTAAAGTTCATTACATTCTAATACATTCCATTC 2634

MZT3a CATTGCATTCCATTCCATTCCATTCGATGCCATTCGATTCCATTCCATGCCAAATCATTG 2684

MZT3b CATTGCATTCCATTCCATTCCATTCGATGCCATTCGATTCCATTCCATGCCAAATCATTG 2694

MZT3a CATTCCTTTCCATTCCGTTCCTATCAATTCCATTCCATTCGATTTAGTTCGATTCTATTC 2744

MZT3b CATTCCTTTCCATTCCGTTCCTATCAATTCCATTCCATTCGATTTAGTTCGATTCTATTC 2754

MZT3a ACTTCCATTCCATTCGATTCCATTCCATTGGAGTCAATTCCTTTCGACACCCAGCCTTTC 2804

MZT3b ACTTCCATTCCATTCGATTCCATTCCATTGGAGTCAATTCCTTTCGACACCCAGCCTTTC 2814

MZT3a CAGTCAATGATTTTGGATTCCATTTTGTTGCATTCCATTACATTCTATGACATTCGATTC 2864

MZT3b CAGTCAATGATATTGGATTCCATTTTGTTGCATTCCATTACATTCTATGACATTCGATTC 2874

MZT3a CGTTTCATTGAATTCCATTCCATACATTTTTATTCCATTCGAGACCGTAGCATTCCACTT 2924

MZT3b CATTTCATTGCATTCCATTCCATACATTTTTATTCCATTCGAGACCGTAGCATTCCACTT 2934

MZT3a TATTCCAGG 2933

MZT3b TATTCCAGG 2943
