# Supplementary material for: DYZ1 arrays show sequence variation between the monozygotic males
Source: BMC Genet. 2014 Feb 4;15:19. doi: 10.1186/1471-2156-15-19 (PMC3925983; doi:10.1186/1471-2156-15-19)
Supplement: Additional file 3 — Multiple Sequence Alignment (MSA) of 3.56 Kb sequence of DYZ1 array from Blood DNA with Germline DNA of MZT1. The insertions, deletions and point mutations are highlighted in yellow. (A) MZT1a and (B) MZT1b. [file 1471-2156-15-19-S3.docx]

**Additional file 3**: **Multiple Sequence Alignment (MSA) of 3.56Kb sequence of DYZ1array from Blood DNA with Germline DNA of MZT1**. The insertions, deletions and point mutations are highlighted in yellow. (A) MZT1a and (B) MZT1b.

**(A)**

MZT1a ((Blood)) CCTGTCCATTACACTACATTCCCTTCCATTCCAATGAATTCCATTCCATTCCAATCCATT 60

MZT1a(Germline) CCTGTCCATTACACTACATTCCCTTCCATTCCAATGAATTCCATTCCATTCCAATCCATT 60

MZT1a(Blood) CCTTTCCTTTCGCTTGCATTCCATTCTATTCCCTTCTACTGCATACAATTTCACTCCATT 120

MZT1a(Germline) CCTTTCCTTTCGCTTGCATTCCATTCTATTCCCTTCTACTGCATACAATTTCACTCCATT 120

MZT1a(Blood) CGTTCCCATTCCATTCAATTCCATTCCATTCAATTCCATTCCATTTGTTTCCATTCTCTT 180

MZT1a(Germline) CGTTCCCATTCCATTCAATTCCATTCCATTCAATTCCATTCCATTTGTTTCCATTCTCTT 180

MZT1a(Blood) CGATTCCATTTCTTTATATTCCATGCCATTCGATTCCATTCTATTGGATTGCATTACATT 240

MZT1a(Germline) CGAATCCATTTCTTTATATTCCATGCCATTCGATTCCATTCTATTGGATTGCATTACATT 240

MZT1a(Blood) CGTGTTCATTCCATTCCAGACCATTCCATTTGACTCCATTCCTTTCGAGCCCTTTCAATT 300

MZT1a(Germline) CGTGTTCATTCCATTCCAGACCATTCCATTTGACTCCATTCCTTTCGAGCCCTTTCAATT 300

MZT1a(Blood) TGAGTCCATTCCTTTCCAGTCCATTTCACTCCAGTCCATTACTATCCATTCCATACCATT 360

MZT1a(Germline) TGAGTCCATTCCTTTCCAGTCCATTTCACTCCAGTCCATTACTATCCATTCCATACCATT 360

MZT1a(Blood) CCATCCCATTCCATTCCATTCCATTCCATTCCATTCCATTCCATTCCATTGCATTCCATT 420

MZT1a(Germline) CCATCCCATTCCATTCCATTCCATTCCATTCCATTCCATTCCATTCCATT-----CAATT 415

MZT1a(Blood) CCATTCCATTCCATTGCACTGCACTCCATTCCATTACATTCTACTCTATCTGAGTCGATT 480

MZT1a(Germline) CCATTCCATTCCATTGCACTGCACTCCATTCCATTACATTCTACTCTATCTGAGTCGGTT 475

MZT1a(Blood) TTATTGCATTAGATTCTATTCCATTGGATTACTTTCCATTCGATTACATTCCATTCATGT 540

MZT1a(Germline) TTATTGCATTAGATTCTATTCCATTGGATTACTTTCCATTCGATTACCTTCCATTCATGT 535

MZT1a(Blood) ACATTCCATTCCAGTCAATTACATTCGAGTTCATTACATTACATTCCAGTATATTCCATT 600

MZT1a(Germline) ACATTCCATTCCAGTCAATTACATTCGAGTTCATTACATTACATTCCAGTATATTCCATT 595

MZT1a(Blood) GTATTCGATCCCATTCCTTTCAATTCCATTTCATTCGACTCCATTATATTCGATTCCATT 660

MZT1a(Germline) GTATTCGATCCCATTCCTTTCAATTCCATTTCATTCGACTCCATTATATTCGATTCCATT 655

MZT1a(Blood) CCACTCGAATCCATTCCATTAGAGGACATTCCATTCCAATGCATTCCATTCCATTCCATA 720

MZT1a(Germline) CCACTCGAATCCATTCCATTAGAGGACATTCCATTCCAATGCATTCCATTCCATTCCATA 715

MZT1a(Blood) GCATTCCATTGCATTCGATTCCATTCCATTTGATGCCATTCCATTTGATGCCATTCCATG 780

MZT1a(Germline) GCATTCCATTGCATTCGATTCCATTCCATTTGATGCCATTCCATTTGATGCCATTCCATG 775

MZT1a(Blood) ACATTCCATTCCATTCGAGTCCATTCCGTTCCAATTCATTCCATTCCGTTTCATGAAATT 840

MZT1a(Germline) ACATTCCATTCCATTCGAGTCCATTCCGTTCCAATTCATTCCATTCCGTTTCATGAAATT 835

MZT1a(Blood) CGAGTCCTTTCCAGTACATTTCATTCCAATCCCATCCAATCCCATCTACTCCATTCAATT 900

MZT1a(Germline) CGAGTCCTTTCCAGTACATTTCATTCCAATCCCATCCAATCCCATCTACTCCATTCAATT 895

MZT1a(Blood) CCTTTCCATTCCATTTGATTTGATTCCATTGATTTGATTCCATTCAGTTTGATTCCATTC 960

MZT1a(Germline) CCTTTCCATTCCATTTGATTTGATTCCATTGATTTGATTCCATTCAGTTTGATTCCATTC 955

MZT1a(Blood) CGTGAAATTTCGTTCCATTCTATTCCATTGCATTACTTTCCATTCAATTCCATTCCATTT 1020

MZT1a(Germline) CGTGAAATTTCGTTCCATTCTATTCCATTGCATTACTTTCCATTCAATTCCATTCCATTT 1015

MZT1a(Blood) CATTTCAGTCCATTCGCTTCCTTTCCTTTCGATTCAATTCCATTTGATTCCACTCCATTC 1080

MZT1a(Germline) CATTTCAGTCCATTCGCTTCCTTTCCTTTCGATTCAATTCCATTTGATTCCACTCCATTC 1075

MZT1a(Blood) TATGCGATTTCATTCCAATCGATTCAATTCCATTCGATGACATTCCTTTCGTTTCCATTC 1140

MZT1a(Germline) TATGCGATTTCATTCCAATCGATTCAATTCCATTCGATGACATTCCTTTCGTTTCCATTC 1135

MZT1a(Blood) CATTCGAGTCCATTTAATTTGAGCATTCGTGTCCATTCTATTCGAGTCCATTCCATTACA 1200

MZT1a(Germline) CATTCGAGTCCATTTAATTTGAGCATTCGTGTCCATTCTATTCGAGTCCATTCCATTACA 1195

MZT1a(Blood) GTCTATTCTATTCCCTTCCATTCCTGTTGATTCAATTTCATTCCCTTCCATTCGATTCCT 1260

MZT1a(Germline) GTCTATTCTATTCCCTTCCATTCGTGTTGATTCAATTTCATTCCCTTCCATTCGATTCCT 1255

MZT1a(Blood) TTCCATTCGATTCCATTCCTTTCCATTCCATTCCATTCGTTCCCATTCCATGTGATTTCA 1320

MZT1a(Germline) TTCCATTCGATTCCATTCCTTTCCATTCCATTCCATTCGTTCCCATTCCATGTGATTTCA 1315

MZT1a(Blood) TTCCATTCCAGTCCATTATATTCGAGTCCACTCCACTCCATTCTATTACATTCAATTCCT 1380

MZT1a(Germline) TTCCATTCCAGTCCATTATATTCGAGTCCACTCCACTCCATTCTATTACATTCAATTCCT 1375

MZT1a(Blood) TTTGAGTCCGTTCCATAACACTCCATTCATTTCGATTCCATTTCTTGCCAGTTTTCTTCC 1440

MZT1a(Germline) TTTGAGTCCGTTCCATAACACTCCATTCATTTCGATTCCATTTCTTGCCAGTTTTCTTCC 1435

MZT1a(Blood) ATTTTATTCCATTCCGTTCGATTCCATTCCATTCGATTGCATTCCATTCGAATCCTTTCC 1500

MZT1a(Germline) ATTTTATTCCATTCCGTTCGATTCCATTCCATTCGATTGCATTCCATTCGAATCCTTTCC 1495

MZT1a(Blood) ATTCCATTTCATTCCATTCCTTTCTATTCCATTCCATTTCATTCGATTTGATTCCATTCT 1560

MZT1a(Germline) ATTCCATTTCATTCCATTCCTTTCTATTCCATTCCATTTCATTCGATTTGATTCCATTCT 1555

MZT1a(Blood) GTTCTATTCCATTCAATTCTTTTTCATTCCATTCGAATCCTTTCTATTGCAGTCCATTCC 1620

MZT1a(Germline) GTTCTATTCCATTCAATTCTTTTTCATTCCATTCGAATCCTTTCTATTGCAGTCCATTCC 1615

MZT1a(Blood) ATTCGAGTCCATTCCAATCCCTTCCATTCCATTCCATTACAGTCCATTCCAATAGATTCC 1680

MZT1a(Germline) ATTCGAGTCCATTCCAATCCCTTCCATTCCATTCCATTACAGTCCATTCCAATAGATTCC 1675

MZT1a(Blood) ATTCCTTTGCCTTCCATTCGAATCCATTCCATTCTAGTCCATTCCATTTGAGTCAATTCC 1740

MZT1a(Germline) ATTCCTTTGCCTTCCATTCGAATCCATTCCATTCTAGTCCATTCCATTTGAGTCAATTCC 1735

MZT1a(Blood) ATTCCATTCCATTCTATTCCTTTCCAATCCATTCGATTCCATTCGATTCAATTCCATTTG 1800

MZT1a(Germline) ATTCCATTCCATTCTATTCCTTTCCAATCCATTCGATTCCATTCGATTCAATTCCATTTG 1795

MZT1a(Blood) ATTCTCTTTCATTCTATTTTATTCCATGCCATTTGATTGCATTGCATTCCATTCCGTTTG 1860

MZT1a(Germline) ATTCTCTTTCATTCTATTTTATTCCATGCCATTTGATTGCATTGCATTCCATTCCGTTTG 1855

MZT1a(Blood) ATTCCAGTCCATTCAAGAAAGTTCCATTCCAGTCCATTGCTTTCCAGTCCATTCCATTCC 1920

MZT1a(Germline) ATTCCAGTCCATTCAAGAAAGTTCCATTCCAGTCCATTGCTTTCCAGTCCATTCCATTCC 1915

MZT1a(Blood) ACTCTAGTCTATTCCACTCCATTCCTTTCCATTCCATTCCATACTATTCCATTCCATTCC 1980

MZT1a(Germline) ACTCTAGTCTATTCCACTCCATTCCTTTCCATTCCATTCCATACTATTCCATTCCATTCC 1975

MZT1a(Blood) TTTGCATTCCGTTTCCAATCTATTCGAGTCCATTGCATTCCAGTCCAATCCATTCGATTA 2040

MZT1a(Germline) TTTGCATTCCGTTTCCAATCTATTCGAGTCCATTGCATTCCAGTCCAATCCATTCGATTA 2035

MZT1a(Blood) CATTCCTTTTGATTCCCTGCCAGTCGATTGCATTGCATACTAGACCATTCCAAAGGAGTT 2100

MZT1a(Germline) CATTCCTTTTGATTCCCTGCCAGTCGATTGCATTGCATACTAGACCATTCCAAAGGAGTC 2095

MZT1a(Blood) CATTCCATTCTATCTCCACACTTTCCATTCCACTCTGTTTGAGTCCATTCCATTCCAGTC 2160

MZT1a(Germline) CATTCCATTCTATCTCAACACTTTCCATTCCACTCTGTTCGAGTCCATTCCATTCCAGTC 2155

MZT1a(Blood) CATTTAATTCAAGGGCATTCCATTCCATTCCATTCCATTCCATTTCATATTATTCCATTC 2220

MZT1a(Germline) CATTTAATTCAAGGGCATTCCATTCCATTCCATTCCATTCCATTTCATATTATTCCATTC 2215

MZT1a(Blood) CATTCAATTCCATTCCAGATGATTCCATTCCATTCTATACCATTGCTCTCTGTTCCATTC 2280

MZT1a(Germline) CATTCAATTCCATTCCAGATGATTCCATTCCATTCTATACCATTGCTCTCTGTTCCATTC 2275

MZT1a(Blood) CATTCCATCTGTCTCCATTCCTTTCGTTTCGATTCCTTTCCATTCCATTCCATTACATTT 2340

MZT1a(Germline) CATTCCATCTGTCTCCATTCCTTTCGTTTCGATTCCTTTCCATTCCATTCCATTACATTT 2335

MZT1a(Blood) GATCCTATTTTATTAAATTGCATTCTATTCGAGTGATTTCCATTCGAGTCCTTTCCATTC 2400

MZT1a(Germline) GATCCTATTTTATTAAATTGCATTCTATTCGAGTGATTTCCATTCGAGTCCTTTCCATTC 2395

MZT1a(Blood) GATTCCATTCCATTCTATTCCATTCCTTTGGATTCCATTCCATTCCGTTCCGTTCACATC 2460

MZT1a(Germline) GATTCCATTCCATTCTATTCCATTCCTTTGGATTCCATTCCATTCCGTTCCGTTCACATC 2455

MZT1a(Blood) AATTCCTTGCGATTCCATTACATTCGATTTCTTGCCATTCGATTCCATTCCTTTTGACTC 2520

MZT1a(Germline) AATTCCTTGCGATTCCATTACATTCGATTTCTTGCCATTCGATTCCATTCCTTTTGACTC 2515

MZT1a(Blood) CATTTCATTCGATTCCATTCCATTCCATTAATTTCCATTCCATTCGAGACCTTTCCATTG 2580

MZT1a(Germline) CATTTCATTCGATTCCATTCCATTCCATTAATTTCCATTCCATTCGAGACCTTTCCATTG 2575

MZT1a(Blood) CAGTCTTTTCCCTTCGAGTCCATTCCGTTCGATTCCCTTCCATTCGATTCCATTCCATTG 2640

MZT1a(Germline) CAGTCTTTTCCCTTCGAGTCCATTCCGTTCGATTCCCTTCCATTCGATTCCATTCCATTG 2635

MZT1a(Blood) GAGTCCGTACCAGTCGAGTCCATTCTATTCCAGTCCATTAGTTTCGACTCCATTGCATTC 2700

MZT1a(Germline) GAGTCCGTACCAGTCGAGTCCATTCTATTCCAGTCCATTAGTTTCGACTCCATTGCATTC 2695

MZT1a(Blood) GAGTGCATTCCATTCCGTGGCTGTCCATTCCATTCCGTTTGATGCCATTCCATACGATTC 2760

MZT1a(Germline) GAGTGCATTCCATTCCGTGGCTGTCCATTCCATTCCGTTTGATGCCATTCCATACGATTC 2755

MZT1a(Blood) CATTCAATTCGAGACCATTCTATTCCTGTCCATTCCTTGTGGTTCGATTCCATTTCACTC 2820

MZT1a(Germline) CATTCAATTCGAGACCATTCTATTCCTGTCCATTCCTTGTGGTTCGATTCCATTTCACTC 2815

MZT1a(Blood) TAGTCCATTCCATTCCATTCAATTCCATTCGACTCTATTCCGTTCCATTCAATTCCATTC 2880

MZT1a(Germline) TAGTCCATTCCATTCCATTCAATTCCATTCGACTCTATTCCGTTCCATTCAATTCCATTC 2875

MZT1a(Blood) CATTCGATTCCATTTTTTTCGAGAACCTTCCATTACACTCCCTTCCATTCCAGTGCATTC 2940

MZT1a(Germline) CATTCGATTCCATTTTTTTCGAGAACCTTCCATTACACTCCCTTCCATTCCAGTGCATTC 2935

MZT1a(Blood) CATTCCAGTCTCTTCAGTTCGATTCCATTCCATTCGTTTCGATTCCTTTCCATTCCAGCC 3000

MZT1a(Germline) CATTCCAGTCTCTTCAGTTCGATTCCATTCCATTCGTTTCGATTCCTTTCCATTCCAGCC 2995

MZT1a(Blood) CATTCCATTCCATTCCATTCCTTTCCTTTCCGTTTCATTAGATTCCATTGCATTCGATTC 3060

MZT1a(Germline) CATTCCATTCCATTCCATTCCTTTCCTTTCCGTTTCATTAGATTCCATTGCATTCCATTC 3055

MZT1a(Blood) CATTCAATTCAATTCCGTGCTATTCAATTTGATTCATTTCCATTTAATTCCATTCCATTA 3120

MZT1a(Germline) CATTCAATTCAATTCCGTGCTATTCAATTTGATTCATTTCCATTTAATTCCATTCCATTA 3115

MZT1a(Blood) GATTCCATTCCGTACGATTCCATTCCTTTTGAATCCATTCCATTGGAGTCCATTCACTTC 3180

MZT1a(Germline) GATTCCATTCCGTACGATTCCATTCCTTTTGAATCCATTCCATTGGAGTCCATTCACTTC 3175

MZT1a(Blood) CAGAACATTCCATTCCAGTCGAATCCATTCGAGTACATTCCATTAAAGTTCATTACATTC 3240

MZT1a(Germline) CAGAACATTCCATTCCAGTCGAATCCATTCGAGTACATTCCATTAAAGTTCATTACATTC 3235

MZT1a(Blood) TAATACATTCCATTCCATTGCATTCCATTCCATTCCATTCGATGCCATTCGATTCCATTC 3300

MZT1a(Germline) TAATACATTCCATTCCATTGCATTCCATTCCATTCCATTCGATGCCATTCGATTCCATTC 3295

MZT1a(Blood) CATGCCAAATCATTGCATTCCTTTCCATTCCGTTCCTATCAATTCCATTCCATTCGATTT 3360

MZT1a(Germline) CATGCCAAATCATTGCATTCCTTTCCATTCCGTTCCTATCAATTCCATTCCATTCGATTT 3355

MZT1a(Blood) AGTTCGATTCTATTCACTTCCATTCCATTCGATTCCATTCCATTGGAGTCAATTCCTTTC 3420

MZT1a(Germline) AGTTCGATTCTATTCACTTCCATTCCATTCGATTCCATTCCATTGGAGTCAATTCCTTTC 3415

MZT1a(Blood) GACACCCAGCCTTTCCAGTCAATGATTTTGGATTCCATTTTTTTGCATTCCATTACATTC 3480

MZT1a(Germline) GACACCCAGCCTTTCCAGTCAATGATTTTGGATTCCATTTTGTTGCATTCCATTACATTC 3475

MZT1a(Blood) TATGACATTCGATTCCGTTTCATTGCATTCCATTCCATACATTTTTATTCCATTCGAGAC 3540

MZT1a(Germline) TATGACATTCGATTCCGTTTCATTGCATTCCATTCCATACATTTTTATTCCATTCGAGAC 3535

MZT1a(Blood) CGTAGCATTCCACTTTATTCCAGG 3564

MZT1a(Germline) CGTAGCATTCCACTTTATTCCAGG 3559

**(B)**

MZT1b(Blood) CCTGTCCATTACACTACATTCCCTTCCATTCCAATGAATTCCATTCCATTCCAATCCATT 60

MZT1b(Germline) CCTGTCCATTACACTACATTCCCTTCCATTCCAATGAATTCCATTCCATTCCAATCCATT 60

MZT1b(Blood) CCTTTCCTTTCGCTTGCATTCCATTCTATTCTCTTCTACTGCATACAATTTCACTCCATT 120

MZT1b(Germline) CCTTTCCTTTCGCTTGCATTCCATTCTATTCTCTTCTACTGCATACAATTTCACTCCATT 120

MZT1b(Blood) CGTTCCCATTCCATTCAATTCCATTCCATTCAATTCCATTCCATTTGTTTCCATTCTCTT 180

MZT1b(Germline) CGTTCCCATTCCATTCAATTCCATTCCATTCAATTCCATTCCATTTGTTTCCATTCTCTT 180

MZT1b(Blood) CGATTCCATTTCTTTATATTCCATGCCATTCGATTCCATTCTATTGGATTGCATTACATT 240

MZT1b(Germline) CGATTCCATTTCTTTATATTCCATGCCATTCGATTCCATTCTATTGGGTTGCATTACATT 240

MZT1b(Blood) CGTGTTCATTCCATTCCAGACCATTCCATTTGACTCCATTCCTTTCGAGCCCTTTCAATT 300

MZT1b(Germline) CGTGTTCATTCCATTCCAGACCATTCCATTTGACTCCATTCCTTTCGAGCCCTTTCAATT 300

MZT1b(Blood) TGAGTCCATTCCTTTCCAGTCCATTTCACTCCAGTCCATTACTATCCATTCCATACCATT 360

MZT1b(Germline) TGAGTCCATTCCTTTCCAGTCCATTTCACTCCAGTCCATTACTATCCATTCCATACCATT 360

MZT1b(Blood) CCATCCCATTCCATTCCATTCCATTCCATT---------------GCATTCCATTCCATT 405

MZT1b(Germline) CCATCCCATTCCATTCCATTCCATTCCATTCCATTCCATTCCATTGCATTCCATTCCATT 420

MZT1b(Blood) CCATTCCATTGCACTGCACTCCATTCCATTACATTCTACTCTATCTGAGTCGATTTTATT 465

MZT1b(Germline) CCATTCCATTGCACTGCACTCCATTCCATTACATTCTACTCTATCTGAGTCGATTTTATT 480

MZT1b(Blood) GCATTAGATTCTATTCCATTGGATTACTTTCCATTCGATTACATTCCATTCATGTACATT 525

MZT1b(Germline) GCATTAGATTCTATTCCATTGGATTACTTTCCATTCGATTACATTCCATTCATGTACATT 540

MZT1b(Blood) CCATTCCAGTCAATTACATTCGAGTTCATTACATTACATTCCAGTATATTCCATTGTATT 585

MZT1b(Germline) CCATTCCAGTCAATTACATTCGAGTTCATTACATTACATTCCAGTATATTCCATTGTATT 600

MZT1b(Blood) CGATCCCATTCCTTTCAATTCCATTTCATTCGACTCCATTATATTCGATTCCATTCCACT 645

MZT1b(Germline) CGATCCCATTCCTTTCAATTCCATTTCATTCGACTCCATTATATTCGATTCCATTCCACT 660

MZT1b(Blood) CGAATCCATTCCATTAGAGGACATTCCATTCCAATGCATTCCATTCCATTCCATAGCATT 705

MZT1b(Germline) CGAATCCATTCCATTAGAGGACATTCCATTCCAATGCATTCCATTCCATTCCATAGCATT 720

MZT1b(Blood) CCATTGCATTCGATTCCATTCCATTTGATGCCATTCCATTTGATGCCATTCCATGACATT 765

MZT1b(Germline) CCATTGCATTCGATTCCATTCCATTTGATGCCATTCCATTTGATGCCATTCCATGACATT 780

MZT1b(Blood) CCATTCCATTCGAGTCCATTCCGTTCCAATTCATTCCATTCCGTTTCATGAAATTCGAGT 825

MZT1b(Germline) CCATTCCATTCGAGTCCATTCCGTTCCAATTCATTCCATTCCGTTTCATGAAATTCGAGT 840

MZT1b(Blood) CCTTTCCAGTACATTTCATTCCAATCCCATCCAATCCCATCTACTCCATTCAATTCCTTT 885

MZT1b(Germline) CCTT-CCAGTACATTTCATTCCAATCCCATCCAATCCCATCTACTCCATTCAATTCCTTT 899

MZT1b(Blood) CCATTCCATTTGATTTGATTCCATTGATTTGATTCCATTCAGTTTGATTCCATTCCGTGA 945

MZT1b(Germline) CCATTCCATTTGATTTGATTCCATTGATTTGATTCCATTCAGTTTGATTCCATTCCGTGA 959

MZT1b(Blood) AATTTCGTTCCATTCTATTCCATTGCATTACTTTCCATTCAATTCCATTCCATTTCATTT 1005

MZT1b(Germline) AATTTCGTTCCATTCTATTCCATTGCATTACTTTCCATTCAATTCCATTCCATTTCATTT 1019

MZT1b(Blood) CAGTCCATTCGCTTCCTTTCCTTTCGATTCAATTCCATTTGATTCCACTCCATTCTATGC 1065

MZT1b(Germline) CAGTCCATTCGCTTCCTTTCCTTTCGATTCAATTCCATTTGATTCCACTCCATTCTATGC 1079

MZT1b(Blood) GATTTCATTCCAATCGATTCAATTCCATTCGATGACATTCCTTTCGTTTCCATTCCATTC 1125

MZT1b(Germline) GATTTCATTCCAATCGATTCAATTCCATTCGATGACATTCCTTTCGTTTCCATTCCATTC 1139

MZT1b(Blood) GAGTCCATTTAATTTGAGCATTCGTGTCCATTCTATTCGAGTCCATTCCATTACAGTCTA 1185

MZT1b(Germline) GAGTCCATTTAATTTGAGCATTCGTGTCCATTCTATTCGAGTCCATTCCATTACAGTCTA 1199

MZT1b(Blood) TTCTATTCCCTTCCATTCGTGTTGATTCAATTTCATTCCCTTCCATTCGATTCCTTTCCA 1245

MZT1b(Germline) TTCTATTCCCTTCCATTCGTGTTGATTCAATTTCATTCCCTTCCATTCGATTCCTTTCCA 1259

MZT1b(Blood) TTCGATTCCATTCCTTTCCATTCCATTCCATTCGTTCCCATTCCATGTGATTTCATTCCA 1305

MZT1b(Germline) TTCGATTCCATTCCTTTCCATTCCATTCCATTCGTTCCCATTCCATGTGATTTCATTCCA 1319

MZT1b(Blood) TTCCAGTCCATTATATTCGAGTCCACTCCACTCCATTCTATTACATTCAATTCCTTTTGA 1365

MZT1b(Germline) TTCCAGTCCATTATATTCGAGTCCACTCCACTCCATTCTATTACATTCAATTCCTTTTGA 1379

MZT1b(Blood) GTCCGTTCCATAACACTCCATTCATTTCGATTCCATTTCTTGCCAGTTTTCTTCCATTTT 1425

MZT1b(Germline) GTCCGTTCCATAACACTCCATTCATTTCGATTCCATTTCTTGCCAGTTTTCTTCCATTTT 1439

MZT1b(Blood) ATTCCATTCCGTTCGATTCCATTCCATTCGATTGCATTCCATTCGAATCCTTTCCATTCC 1485

MZT1b(Germline) ATTCCATTCCGTTCGATTCCATTCCATTCGATTGCATTCCATTCGAATCCTTTCCATTCC 1499

MZT1b(Blood) ATTTCATTCCATTCCTTTCTATTCCATTCCATTTCATTCGATTTGATTCCATTCTGTTCT 1545

MZT1b(Germline) ATTTCATTCCATTCCTTTCTATTCCATTCCATTTCATTCGATTTGATTCCATTCTGTTCT 1559

MZT1b(Blood) ATTCCATTCAATTCTTTTTCATTCCATTCGAATCCTTTCTATTGCAGTCCATTCCATTCG 1605

MZT1b(Germline) ATTCCATTCAATTCTTTTTCATTCCATTCGAATCCTTTCTATTGCAGTCCATTCCATTCG 1619

MZT1b(Blood) AGTCCATTCCAATCCCTTCCATTCCATTCCATTACAGTCCATTCCAATAGATTCCATTCC 1665

MZT1b(Germline) AGTCCATTCCAATCCCTTCCATTCCATTCCATTACAGTCCATTCCAATAGATTCCATTCC 1679

MZT1b(Blood) TTTGCCTTCCATTCGAATCCATTCCATTCTAGTCCATTCCATT---------------CC 1710

MZT1b(Germline) TTTGCCTTCCATTCGAATCCATTCCATTCTAGTCCATTCCATTTGAGTCAATTCCATTCC 1739

MZT1b(Blood) ATTCCATTCTATTCCTTTCCAATCCATTCGATTCCATTCGATTCAATTCCATTTGATTCT 1770

MZT1b(Germline) ATTCCATTCTATTCCTTTCCAATCCATTCGATTCCATTCGATTCAATTCCATTTGATTCT 1799

MZT1b(Blood) CTTTCATTCTATTTTATTCCATGCCATTTGATTGCATTGCATTCCATTCCGTTTGATTCC 1830

MZT1b(Germline) CTTTCATTCTATTTTATTCCATGCCATTTGATTGCATTGCATTCCATTCCGTTTGATTCC 1859

MZT1b(Blood) AGTCCATTCAAGAAAGTTCCATTCCAGTCCATTGCTTTCCAGTCCATTCCATTCCACTCT 1890

MZT1b(Germline) AGTCCATTCAAGAAAGTTCCATTCCAGTCCATTGCTTTCCAGTCCATTCCATTCCACTCT 1919

MZT1b(Blood) AGTCTATTCCACTCCATTCCTTTCCATTCCATTCCATACTATTCCATTCCATTCCTTTGC 1950

MZT1b(Germline) AGTCTATTCCACTCCATTCCTTTCCATTCCATTCCATACTATTCCATTCCATTCCTTTGC 1979

MZT1b(Blood) ATTCCGTTTGCAATCTATTCGAGTCCATTTCATTCCAGTCCAATCCATTCGATTACATTC 2010

MZT1b(Germline) ATTCCGTTTCCAATCTATTCGAGTCCATTGCATTCCAGTCCAATCCATTCGATTACATTC 2039

MZT1b(Blood) CTTTTGATTCCCTGCCTGTCGATTGCATTGCATACTAGACCATTCCAAACGAGTCCATTC 2070

MZT1b(Germline) CTTTTGATTCCCTGCCAGTCGATTGCATTGCATACTACACCATTCCAAAGGAGTCCATTC 2099

MZT1b(Blood) CATTCTATCTCAACACTTTCCATTCCACTCTGTTCGAGTCCATTCCATTCCAGTCCATTT 2130

MZT1b(Germline) CATTCTATCTCAACACTTTCCATTCCACTCTGTTCGAGTCCATTCCATTCCAGTCCATTT 2159

MZT1b(Blood) AATTCAAGGGCATTCCATTCCATTCCATTCCATTCCATTTCATATTATTCCATTCCATTC 2190

MZT1b(Germline) AATTCAAGGGCATTCCATTCCATTCCATTCCATTCCATTTCATATTATTCCATTCCATTC 2219

MZT1b(Blood) AATTCCATTCCAGATGATTCCTTTCCATTCTATACCATTGCTCTCTGTTCTATTCCATTC 2250

MZT1b(Germline) AATTCCATTCCAGATGATTCCATTCCATTCTATACCATTGCTCTCTGTTCCATTCCATTC 2279

MZT1b(Blood) CATCTGTCTCCATTCCTTTCGTTTCGATTCCTTTCCATTCCATTCCATTACATTTGATCC 2310

MZT1b(Germline) CATCTGTCTCCATTCCTTTCGTTTCGATTCCTTTCCATTCCATTCCATTACATTTGATCC 2339

MZT1b(Blood) TATTTTATTAAGTTGCATTCTATTCGAGTGATTTCCATTCGAGTCCTTTCCATTCGATTC 2370

MZT1b(Germline) TATTTTATTAAATTGCATTCTATTCGAGTGATTTCCATTCGAGTCCTTTCCATTCGATTC 2399

MZT1b(Blood) CATTCCATTCTATTCCATTCCTTTGGATTCCATTCCATTCCGTTCCGTTCACATCAATTC 2430

MZT1b(Germline) CATTCCATTCTATTCCATTCCTTTGGATTCCATTCCATTCCGTTCCGTTCACATCAATTC 2459

MZT1b(Blood) CTTGCGATTCCATTACATTCGATTTCTTGCCATTCGATTCCATTCCTTTTGACTCCATTT 2490

MZT1b(Germline) CTTGCGATTCCATTACATTCGATTTCTTGCCATTCAATTCCATTCCTTTTGACTCCATTT 2519

MZT1b(Blood) CATTCGATTCCATTCCATTCCATTAATTTCCATTCCATTCGAGACCTTTCCATTGCAGTC 2550

MZT1b(Germline) CATTCGATTCCATTCCATTCCATTAATTTCCATTCCATTCGAGACCTTTCCATTGCAGTC 2579

MZT1b(Blood) TTTTCCCTTCGAGTCCATTCCGTTCGATTCCCTTCCATTCGATTCCCTTCCATTGGAGTC 2610

MZT1b(Germline) TTTTCCCTTCGAGTCCATTCCGTTCGATTCCCTTCCATTCGATTCCATTCCATTGGAGTC 2639

MZT1b(Blood) CGTACCAGTCGAGTCCATTCTATTCCAGTCCATTAGTTTCGACTCCATTGCATTCGAGTG 2670

MZT1b(Germline) CGTACCAGTCGAGTCCATTCTATTCCAGTCCATTAGTTTCGACTCCATTGCATTCGAGTG 2699

MZT1b(Blood) CATTCCATTCCGTGGCTGTCCATTCCATTCCGTTTGATGCCATTCCATACGATTCCATTC 2730

MZT1b(Germline) CATTCCATTCCGTGGCTGTCCATTCCATTCCGTTTGATGCCATTCCATACGATTCCATTC 2759

MZT1b(Blood) AATTCGAGACCATTCTATTCCTGTCCATTCCTTGTGGTTCGATTCCATTTCACTCTAGTC 2790

MZT1b(Germline) AATTCGAGACCATTCTATTCCTGTCCATTCCTTGTGGTTCGATTCCATTTCACTCTAGTC 2819

MZT1b(Blood) CATTCCATTCCATTCAATTCCATTCGACTCTATTCCGTTCCATTCAATTCCATTCCATTC 2850

MZT1b(Germline) CATTCCATTCCATTCAATTCCATTCGACTCTATTCCGTTCCATTCAATTCCATTCCATTC 2879

MZT1b(Blood) GATTCCATTTTTTTCGAGAACCTTCCATTACACTCCCTTCCATTCCAGTGCATTCCATTC 2910

MZT1b(Germline) GATTCCATTTTTTTCGAGAACCTTCCATTACACTCCCTTCCATTCCAGTGCATTCCATTC 2939

MZT1b(Blood) CAGTCTCTTCAGTTCGATTCCATTCCATTCGTTTCGATTCCTTTCCATTCCAGCCCATTC 2970

MZT1b(Germline) CAGTCTCTTCAGTTCGATTCCATTCCATTCGTTTCGATTCCTTTCCATTCCAGCCCATTC 2999

MZT1b(Blood) CATTCCATTCCATTCCTTTCCTTTCCGTTTCATTAGATTCCATTGCATTCGATTCCATTC 3030

MZT1b(Germline) CATTCCATTCCATTCCTTTCCTTTCCGTTTCATTAGATTCCATTGCATTCGATTCCATTC 3059

MZT1b(Blood) AATTCAATTCCGTGCTATTCAATTTGATTCATTTCCATTTAATTCCATTCCATTAGATTC 3090

MZT1b(Germline) AATTCAATTCCGTGCTATTCAATTTGATTCATTTCCATTTAATTCCATTCCATTAGATTC 3119

MZT1b(Blood) CATTCCGTACGATTCCATTCCTTTTGAATCCATTCCATTGGAGTCCATTCACTTCCAGAA 3150

MZT1b(Germline) CATTCCGTACGATTCCATTCCTTTTGAATCCATTCCATTGGAGTCCATTCACTTCCAGAA 3179

MZT1b(Blood) CATTCCATTCCAGTCGAATCCATTCGAGTACATTCCATTAAAGTTCATTACATTCTAATA 3210

MZT1b(Germline) CATTCCATTCCAGTCGAATCCATTCGAGTACATTCCATTAATGTTCATTACATTCTAATA 3239

MZT1b(Blood) CATTCCATTCCATTGCATTCCATTCCATTCCATTCGATGCCATTCGATTCCATTCCATGC 3270

MZT1b(Germline) CATTCCATTCCATTGCATTCCATTCCATTCCATTCGATGCCATTCGATTCCATTCCATGC 3299

MZT1b(Blood) CAAATCATTGCATTCCTTTCCATTCCGTTCCTATCAATTCCATTCCATTCGATTTAGTTC 3330

MZT1b(Germline) CAAATCATTGCATTCCTTTCCATTCCGTTCCTATCAATTCCATTCCATTCGATTTAGTTC 3359

MZT1b(Blood) GATTCTATTCACTTCCATTCCATTCGATTCCATTCCATTGGAGTCAATTCCTTTCGACAC 3390

MZT1b(Germline) GATTCTATTCACTTCCATTCCATTCGATTCCATACCATTGGAGTCAATTCCTTTCGACGC 3419

MZT1b(Blood) CCAGCCTTTCCAGTCAATGATTTTGGATTCCATTTTTTTGCATTCCATTACATTCTATGA 3450

MZT1b(Germline) CCAGCCTTTCCAGTCAATGATTTTGGATTCCATTTTTTTGCATTCCATTACATTCTATGA 3479

MZT1b(Blood) CATTCGATTCCGTTTCATTGCATTCCATTCCATACATTTTTATTCCATTCGAGACCGTAG 3510

MZT1b(Germline) CATTCGAT-CCGTTTCATTGCATTCCATTCCATACATTTT-ATTCCATTCGAGACCGTAG 3537

MZT1b(Blood) CATTCCACTTTATTCCAGG 3529

MZT1b(Germline) CAT-CCACTTTATTCCAGG 3555
